# Recommendations on maximising the clinical value of tissue in the management of patients with intrahepatic cholangiocarcinoma

**DOI:** 10.1016/j.jhepr.2024.101067

**Published:** 2024-03-12

**Authors:** Timothy Kendall, Diletta Overi, Maria Guido, Chiara Braconi, Jesus Banales, Vincenzo Cardinale, Eugenio Gaudio, Bas Groot Koerkamp, Guido Carpino

**Affiliations:** 1 University of Edinburgh Centre for Inflammation Research and Edinburgh Pathology, University of Edinburgh, Edinburgh, UK; 2 Department of Anatomical, Histological, Forensic Medicine and Orthopedics Sciences, Sapienza University of Rome, Rome, Italy; 3 Department of Medicine, DIMED, University of Padua, Padua, Italy; 4 School of Cancer Sciences, University of Glasgow, CRUK Scotland Cancer Centre, Beatson West of Scotland Cancer Centre, Glasgow, UK; 5 Department of Liver and Gastrointestinal Diseases, Biodonostia Health Research Institute, Donostia University Hospital, CIBERehd and University of the Basque Country (UPV/EHU), San Sebastian, Spain; 6 Department of Biochemistry and Genetics, School of Sciences, University of Navarra, Pamplona, Spain; 7 Department of Translational and Precision Medicine, Sapienza University of Rome, Rome, Italy; 8 Department of Surgery, Erasmus MC Cancer Institute, Rotterdam, the Netherlands

**Keywords:** Cholangiocarcinoma, Guidelines, Histopathology, Management, Molecular testing, Multidisciplinary

## Abstract

**Background & Aims:**

Patients with intrahepatic cholangiocarcinoma can now be managed with targeted therapies directed against specific molecular alterations. Consequently, tissue samples submitted to the pathology department must produce molecular information in addition to a diagnosis or, for resection specimens, staging information. The pathologist’s role when evaluating these specimens has therefore changed to accommodate such personalised approaches.

**Methods:**

We developed recommendations and guidance for pathologists by conducting a systematic review of existing guidance to generate candidate statements followed by an international Delphi process. Fifty-nine pathologists from 28 countries in six continents rated statements mapped to all elements of the specimen pathway from receipt in the pathology department to authorisation of the final written report. A separate survey of ‘end-users’ of the report including surgeons, oncologists, and gastroenterologists was undertaken to evaluate what information should be included in the written report to enable appropriate patient management.

**Results:**

Forty-eight statements reached consensus for inclusion in the guidance including 10 statements about the content of the written report that also reached consensus by end-user participants. A reporting proforma to allow easy inclusion of the recommended data points was developed.

**Conclusions:**

These guiding principles and recommendations provide a framework to allow pathologists reporting on patients with intrahepatic cholangiocarcinoma to maximise the informational yield of specimens required for personalised patient management.

**Impact and Implications:**

Biopsy or resection lesional tissue from intrahepatic cholangiocarcinoma must yield information about the molecular abnormalities within the tumour that define suitability for personalised therapies in addition to a diagnosis and staging information. Here, we have developed international consensus guidance for pathologists that report such cases using a Delphi process that sought the views of both pathologists and ‘end-users of pathology reports. The guide highlights the need to report cases in a way that preserves tissue for molecular testing and emphasises that reporting requires interpretation of histological characteristics within the broader clinical and radiological context. The guide will allow pathologists to report cases of intrahepatic cholangiocarcinoma in a uniform manner that maximises the value of the tissue received to facilitate optimal multidisciplinary patient management.

## Highlights

• A new pathological approach to intrahepatic cholangiocarcinoma can maximise the value of tissue biopsy.
• During routine reporting, tissue should be preserved for future molecular testing.
• Definitive diagnosis requires correlation with clinical and radiological features.
• Pathologists in multidisciplinary meetings support optimal patient management.

## Graphical abstract

**Figure:**
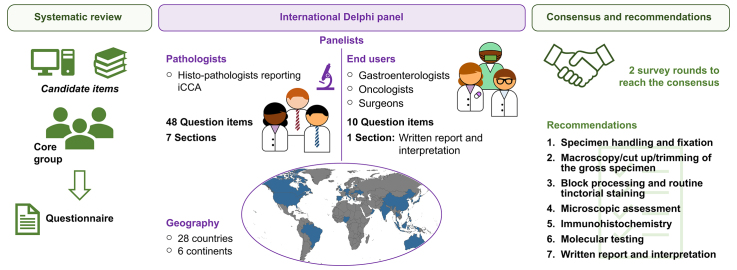

## Introduction

Cholangiocarcinoma (CCA) is the most common malignancy of the biliary tree. It is divided into intrahepatic (iCCA), perihilar (pCCA), and distal (dCCA) types; each a separate entity with distinct clinical, histopathological, and molecular features. iCCAs arise within the liver parenchyma from intrahepatic ducts proximal to second-order bile ducts, and are commonly mass-forming lesions, featuring molecular abnormalities with potential therapeutic targets in up to 50% of cases.

Although guidelines for the management of patients with iCCA are available, recommendations for the pathological pathway, with reference to specimen receipt, tissue storage, tissue processing, analyses to be performed and generation of the final report, are lacking. As personalisation based on mutational profile is increasingly common in routine clinical practice, for example through FDA- and EMA-licensed therapies for patients whose tumours bear *FGFR2* fusions or *IDH1* mutations,^1^ we sought to define recommendations that serve to maximise the overall clinical value of tissue sampling for the management of patients with iCCA. The guidance emphasises when and how molecular characterisation can be effectively undertaken within a routine reporting pathway. Patient management is the most effective when using a multidisciplinary team approach, so we also sought the input of actual end-users of the final authorised pathological report to make our pathway clinically impactful.

Herein, we describe the development of consensus recommendations achieved through an international Delphi process. The adoption of these recommendations will maximise the clinical value of tissue samples for the management of patients with iCCA. For each recommendation, we provide a rationale and relevant references providing context and supporting its utility.

## Materials and methods

### Systematic review

We undertook a systematic review of published expert opinions^2^ on the subject of histological reporting of iCCA cases to inform the generation of Delphi candidate items for subsequent rating. Full details are available in the Supplementary Methods.

#### International Delphi exercise

Candidate Delphi items were generated by synthesising the extracted guidance identified by the systematic review. Items were grouped into the stages of the reporting pathway.

The core research group (TK, MG, GC, DO) revised items to make them thematically coherent and to ensure that parts of the reporting pathway were fully addressed.

Respondents were asked to rate the importance of each item on a nine-point Likert-type scale. Pathologist respondents rated all items, and “end-users” (*i.e.*, surgeons, oncologists, gastroenterologists) of histopathology reports participated in a sub-survey rating only items relating to the final written pathology report. Two rounds were undertaken to reach consensus. Full details of the Delphi process are provided in the Supplementary Methods.

## Results

### Systematic review

#### Selection of guidance resources

The literature searches identified 1,742 records, 312 from Medline (Ovid), 1,366 from Embase (Ovid), and six from the Cochrane database. Web searches identified another 56 unique records. No grey literature was found. The titles and abstracts of 1,384 database records were screened after de-duplication. Of these, 1,371 records from the database searches were excluded by titles and abstracts: 53 of 58 records from Google/Google Scholar searches were excluded by title, and five records were duplicates of reports assessed for eligibility by full-text review from database searches. Of the 13 full-text articles retrieved and assessed for eligibility, 11 were included in the review and two were excluded as records that had been superseded by other records within those included issued by the same organisation (Fig. S1).^3^

#### Characteristics and mapping of guidance resources

Extracted statements from the individual resources were mapped onto the stages of the specimen pathway, and included specimen handling and fixation, macroscopy/slicing/trimming of the gross specimen, block processing, routine tinctorial staining, microscopic assessment, immunohistochemistry, molecular testing, and written report and interpretation (Table S1). Of the 11 guidance resources included,[4], [5], [6], [7], [8], [9], [10], [11], [12], [13], [14] ten offered recommendations about the microscopic assessment[4], [5], [6]^,^[8], [9], [10], [11], [12], [13], [14] and seven contained recommendations about macroscopic assessment,^5^^,^^6^^,^^8^^,^^9^^,^[11], [12], [13] immunohistochemical evaluation,[4], [5], [6], [7], [8], [9]^,^^14^ and the content of the final authorised report.^4^^,^^5^^,^[8], [9], [10]^,^^12^^,^^13^ Eight resources contained explicit recommendations,[4], [5], [6], [7]^,^^9^^,^[11], [12], [13] two contained implicit recommendations,^10^^,^^14^ and one had a combination of implicit and explicit recommendations.^8^

The verbatim extracted guidance from each source, mapped to the specimen pathway categories, is provided in Supplementary Text 2. The extracted guidance from each source was synthesised thematically and was used to generate the initial candidate Delphi statements.

#### Quality assessment of individual guidance resources

The majority of the guidance resources were high quality (Appraisal of Guidelines for Research & Evaluation Global Rating Scale [AGREE-GRS] score of >66%). However, the quality was lower (AGREE-GRS score of <34%) in the AGREE-GRS domains of ‘process of development’ and ‘completeness of reporting’. All guidance resources were considered highly relevant and applicable to reporting practice (Fig. S2).

### The Delphi process

Characteristics of the Delphi participants (Supplementary Text 3) are summarised in Table S2. Full details of the Delphi responses are shown in Table S3, and the process is outlined in Fig. S3.

#### Pathologists

For the full survey of pathologists, invitations to participate in Round One of the Delphi exercise were sent to 97 individuals. Of these, 59 pathologists from 28 countries across Asia, Africa, Europe, North America, Oceania, and South America consented to participate and completed Round One.

Fifty statements were assessed: 44 reached consensus-in status and the remaining six did not reach consensus (items #1, #2, #25, #26, #27, #40). All statements regarding information that should be included in the written report were classified as consensus-in.

There was no variation in opinion or consensus based on the location of participants.

Statements not reaching consensus were revised or combined based upon participant comments. Three statements (#1, #2, #27) were revised. Two statements (#25, #26) were combined into a single revised statement. One statement (#40) was removed as it was originally included as one of a mutually exclusive pair with statement #39.

Invitations for Round Two were sent to those participants who responded in Round One. Fifty-three participants amended their scores from Round One; those who did not had their Round One scores carried forward to Round Two. All four of the remaining modified items reached consensus-in status.

#### End-users

Sixty-five end-users were invited to participate in Round One of the end-user survey. Nineteen participants completed Round One of the survey rating statements about what should be included in the pathology written report. All ten statements reached consensus-in status.

### Consensus recommendations maximising the clinical value of tissue and histopathological reporting for the multidisciplinary management of patients with intrahepatic cholangiocarcinoma

The consensus statements providing guidance for the histopathological reporting of iCCA were grouped by their position in the reporting pathway between receipt of the specimen in the pathology department and the authorisation of the final written pathology report as summarised in Table 1. The agreed statements and an elaboration of their relevance and context are provided, and a reporting proforma capturing all recommended data points has been developed (Supplementary Files 1 and 2).

**Table 1:** Consensus recommendation statements for maximising the clinical value of tissue in the reporting of intrahepatic cholangiocarcinoma.

Item number	Recommendation
**Specimen handling and fixation**	
1	Although more relevant in resections of perihilar rather than intrahepatic cholangiocarcinomas, assessment of the bile duct margin by intraoperative frozen section may be advantageous if the surgeon considers it to be close to the tumour.
2	If tumour sampling is routinely undertaken for research, biobanking, or molecular testing and the specimen will be received and handled by the pathology department without delay, the whole tumour resection specimen should be sent fresh (not in fixative) to the pathology department.
3	Where possible, tumour should be sampled fresh and cryopreserved for molecular analysis.
4	Tissues should be fixed in formalin for at least 24 h before macroscopic examination and block taking (for resections) or routine processing (for biopsies).

**Macroscopy/slicing/trimming of the gross specimen**	
*Dissection*	
5	Resection surfaces other than the liver capsule should be painted/inked.
6	The specimen should be sliced into slices no more than 10 mm thick in the horizontal plane.
*Macroscopic description*	
7	The macroscopic growth pattern (mass-forming, periductal infiltrating, intraductal, mixed) should be described.
8	The size of tumours should be recorded.
9	The location of tumours should be recorded.
10	The macroscopic status of any resection margin(s) and nearest distance to tumour should be recorded.
11	The presence or absence of macroscopic vascular invasion should be recorded.
12	The presence or absence of macroscopic invasion of named bile ducts should be recorded.
13	The integrity of the liver capsule should be recorded.
14	The macroscopic appearance of the non-lesional liver should be recorded.
*Block sampling*	
15	If the tumour is close enough, a single intact tissue block containing tumour and the nearest resection margin should be taken.
16	Tumour with adjacent non-lesional liver should be sampled.
17	Peripheral liver with the closest overlying liver capsule should be sampled if the tumour is subjacent, overlying tissue is adherent, or there is macroscopic invasion of the liver capsule.
18	The gallbladder bed and wall should be sampled where the tumour is adjacent.
19	Areas suspicious for macrovascular invasion should be sampled.
20	Areas suspicious for bile duct invasion should be sampled.
21	Any bile duct resection margin(s) should be sampled.
22	Non-lesional liver distant from the tumour should be sampled.
23	An intratumoural block suitable for molecular testing should be taken or identified.

**Block processing and routine tinctorial staining**	
24	Five micron or thinner H&E-stained sections should be prepared from each block.

**Microscopic assessment**	
25	Intrahepatic cholangiocarcinoma is usually adenocarcinoma with identifiable glands. However, poorly-differentiated solid cholangiocarcinomas and rarer morphological subtypes (*e.g.* adenosquamous, squamous, mucinous, signet-ring, clear cell, mucoepidermoid, lymphoepithelioma-like, and sarcomatous) are described.
26	Desmoplastic stroma is often a prominent feature of intrahepatic cholangiocarcinoma. However, its absence does not preclude a diagnosis of cholangiocarcinoma as its distribution within a tumour may mean it is not present in needle biopsy material.
27	Biliary intraepithelial neoplasia (BilIN) adjacent to an adenocarcinoma with desmoplastic stroma is highly suggestive of cholangiocarcinoma.
28	Large duct-type intrahepatic cholangiocarcinoma is composed of columnar cells.
29	The cells of large duct-type intrahepatic cholangiocarcinoma are mucin-producing.
30	The cells of large duct-type intrahepatic cholangiocarcinoma are usually arranged in large malignant duct formations or have papillary architecture.
31	Small duct-type intrahepatic cholangiocarcinomas usually show no or minimal mucin production.
32	Small duct-type intrahepatic cholangiocarcinomas have a predominant component of cuboidal to polygonal cells.
33	Small duct-type intrahepatic cholangiocarcinoma cells are predominantly arranged as small to intermediate-sized tubules and/or anastomosing glands.

**Immunohistochemistry**	
34	No immunohistochemical pattern is specific for intrahepatic cholangiocarcinoma.
35	Distinction of intrahepatic cholangiocarcinoma from a liver metastasis of a primary upper gastrointestinal, pancreatic, or extrahepatic biliary tumour often cannot be made based on morphology and immunohistochemical profile.
36	Intrahepatic cholangiocarcinomas express typical pancreaticobiliary cytokeratins.
37	Immunohistochemistry can help differentiate intrahepatic cholangiocarcinoma from hepatocellular carcinoma.

**Molecular testing**	
38	Molecular testing, where available, including for *FGFR* abnormalities, *IDH* mutations, and any other abnormality with therapeutic significance should be undertaken.

**Written report and interpretation**	
39	The AJCC/UICCA staging schema should be used for the reporting of resection specimens, and the stage included in the primary report.
40	The results of any molecular analysis and immunohistochemical staining should be included in the primary report.
41	The presence or absence of perineural invasion should be stated in the primary report.
42	The presence or absence of lymphovascular invasion should be stated in the primary report.
43	The presence or absence of dysplasia should be stated in the primary report.
44	Small-duct or large-duct designation of intrahepatic cholangiocarcinomas should be stated in the primary report.
45	A semiquantitative grading based on the proportion of glands should be stated in the primary report (well-differentiated, >95% of tumour composed of glands; moderately-differentiated, 50%–95% of tumour composed of glands; poorly-differentiated, <49% of tumour composed of glands).
46	The status of any resection margin(s) should be stated in the primary report.
47	The status of any lymph nodes should be stated in the primary report.
48	Confident diagnosis requires the correlation of histomorphology and any immunohistochemical staining profile with available imaging and clinical information. Clinical history, endoscopic investigation, and imaging studies are all pivotal in reaching the correct conclusion.

AJCC, American Joint Committee on Cancer; UICCA, International Union Against Cancer.

#### Specimen handling and fixation

1. *Although more relevant in resections of perihilar rather than intrahepatic CCAs, assessment of the bile duct margin by intraoperative frozen section may be advantageous if the surgeon considers it to be close to the tumour.*

Data regarding the use of intraoperative frozen sections to assess the bile duct margin are restricted to patients with pCCA where the anatomy and tumour growth pattern present specific surgical challenges. Even in this setting, the data are contradictory about whether routine assessment of the bile duct margin offers a survival advantage.^15^^,^^16^ No specific studies have examined frozen sections for resections of iCCA. The consensus recommendation is that submission of the bile duct margin for histopathological examination by intraoperative frozen section should be at the discretion of the operating surgeon if the tumour is macroscopically close to the margin. Intraoperative frozen sections may also be useful in the diagnosis of potential intrahepatic metastatic lesions whose nature influences the immediate surgical management.^17^

2. *If tumour sampling is routinely undertaken for research, biobanking, or molecular testing, the specimen should be received and handled by the pathology department without delay. The whole tumour resection specimen should be sent fresh (not in fixative) to the pathology department.*
3. *Where possible, the tumour should be sampled fresh and cryopreserved for molecular analysis.*

Molecular assays used in clinical practice typically rely on formalin-fixed paraffin-embedded (FFPE) tissue, and such tested FFPE material is increasingly reliable and has become a core part of clinical workflows as technologies evolve.[18], [19], [20] However, fresh or frozen material may allow additional testing strategies to be developed and can facilitate fundamental research, for example with tumour-derived organoids. Any sampling of specimens for research purposes should only be undertaken after all required ethical approvals have been obtained and their conditions, including informed consent, have been satisfied.

If authorised sampling of tumours in resection specimens is required, and it can be undertaken in the pathology laboratory prior to fixation without impacting upon the standard clinical specimen pathway and compromising resection margins (*e.g.* as undertaken in the HepaT1ca study^21^), the specimen should be sent fresh and the tumour sampled as soon as possible as significant biochemical alterations can be identified even after only 10 min of tissue anoxia and histological alterations, such as a loss of identifiable mitotic figures, can be observed with a fixation delay of only 2 h.^22^

4. *Tissues should be fixed in formalin for at least 24 h before macroscopic examination and block sampling (for resections) or routine processing (for biopsies).*

We recommend that specimens be fixed in formalin following the standard local procedures and for at least 24 h. Fixation preserves tumour morphology and optimises the performance of clinical immunohistochemistry. Appropriate fixation of resection specimens allows these to be optimally cut and evaluated macroscopically.

#### Macroscopy, slicing, and trimming of the gross specimen

Macroscopic assessment of, and block sampling from gross resection specimens should be undertaken in a coordinated manner that allows the required information from the specimen to be obtained. Some recommendations below relate to information with proven clinical utility. Others relate to pathological macroscopic features that have a strong biological rationale but require further study that will be enabled but routine data collection.

##### Dissection

5. *Resection surfaces other than the liver capsule should be painted/inked.*

Tissue-marking dyes are commonly applied to resection specimens prior to any dissection or sectioning to mark both surgical margins and other landmarks, and the use of different colours allows the orientation of specimens to be preserved; we recommend the use of such dyes. In resections for intrahepatic lesions, including iCCA, only a single margin is usually found. The presence of ink on the subsequent histological section allows confident identification of the surgical margin so that the completeness of resection and surgical clearance can be determined. The inked resection margins should ideally be ‘fixed’ and dried before sectioning to reduce the likelihood that surface ink is introduced into the specimen upon slicing.^23^ The surgeon may indicate the vascular margin in cases where this is relevant.

6. *The specimen should be sliced into slices no more than 10 mm thick in the horizontal plane.*

We recommend that initial slicing of a resection specimen should only be undertaken once it is well fixed to ensure that slices no thicker than 10 mm can be made. These initial slices should be made perpendicular to the resection margin(s), often in the axial plane. Sectioning at 10 mm or less allows small lesions to be identified and allows the surgical clearance margin to be evaluated.

##### Macroscopic description

After initial sectioning of the resection specimen, macroscopic description of many of the core dataset items that are known to have prognostic or therapeutic importance can be undertaken.

7. *The macroscopic growth pattern (mass-forming, periductal infiltrating, intraductal, mixed) should be described.*

The macroscopic growth pattern of CCA can be mass-forming, periductal infiltrating, intraductal, or mixed although mass-forming is the most common pattern for iCCA. Patients with iCCAs that have a pure mass-forming macroscopic growth pattern probably have a better prognosis than patients with periductal infiltrating tumours although pure intraductal tumours may have a better prognosis.[24], [25], [26] We recommend that the macroscopic growth pattern be assessed at the time of specimen dissection.

8. *The size of tumours should be recorded.*

The size of each tumour should be recorded individually, which also captures the total number of tumours. This provides tumour burden, a function of tumour number and tumour size, which is of prognostic significance; patients with tumours larger and more than a single tumour having a worse prognosis.[27], [28], [29], [30] In a study of explant livers, patients with single tumours ≤2 cm had very low risk of recurrence and 73% 5-year survival.^31^

Further, a recent study has demonstrated that in the absence of lymph node or extrahepatic spread, patients with iCCA and liver metastases have a worse outcome than patients without liver metastases.^32^ Recording the size and number of tumours will allow re-staging of patients should staging systems be modified in light of this data.

9. *The location of tumours should be recorded.*

We recommend that recording of the location of tumours within specimens should be attempted although it is not always easy to do so with complete accuracy. An estimation of which Couinaud segment(s) a tumour is in, and/or its relative location (anterior/posterior, peripheral/central) may allow attempts at correlation with cross-sectional imaging. Classification of the tumour as intrahepatic should be confirmed during macroscopic assessment. If a tumour arises from the first and second order bile ducts it should be classified as a perihilar lesion and is beyond the scope of these guidelines.

10. *The macroscopic status of any resection margin(s) and nearest distance to tumour should be recorded.*

Evaluation of the margin and measurement of the shortest distance from tumour to surgical margin provides significant prognostic information and should be undertaken. R0 resection status and increasing clearance distance are both associated with better prognosis,^30^^,^[33], [34], [35], [36] and the data is also used in routine audits of surgical practice.

11. *The presence or absence of macroscopic vascular invasion should be recorded.*

We recommend that the presence or absence of vascular invasion should be recorded. Macroscopically identified vascular invasion of large portal or hepatic veins is a very poor prognostic factor in iCCA.^37^^,^^38^ Often this can be identified pre-operatively and may be a contraindication to surgery.

12. *The presence or absence of macroscopic invasion of named bile ducts should be recorded.*

Invasion of extrahepatic structures including named bile ducts is considered pT4 in the Union for International Cancer Control TNM 8th edition and is associated with poor overall prognosis,^39^ and this information should be documented. Bile duct invasion has separately demonstrated to be an adverse prognostic factor when assessed on preoperative imaging.^40^

13. *The integrity of the liver capsule should be recorded.*

Glisson’s capsule is the thin layer of interstitial connective tissue surrounding most of the external surface of the liver and extends around hilar vessels. Invasion into or through the capsule should be recorded as it may represent a route to metastasis although specific survival data examining this are lacking.^41^

14. *The macroscopic appearance of the non-lesional liver should be recorded.*

Cirrhosis of any cause is a risk factor for the development of iCCA,^30^^,^^42^ so the appearance of the non-lesional liver should be commented upon. Although the data about the influence of cirrhosis on outcomes after surgery for iCCA is conflicting,^43^^,^^44^ the subsequent investigation and management of patients newly diagnosed as having cirrhosis will be different from a patient with no background liver abnormality.

##### Block sampling

15. *If the tumour is close enough, a single intact tissue block containing tumour and the nearest resection margin should be taken.*

We recommend that a block showing the relationship of the tumour to the closest margin be taken. This allows microscopic confirmation of the resection margin status and measurement of the clearance distance, a metric predictive of disease-free and overall survival.^35^

16. *Tumour with adjacent non-lesional liver should be sampled.*

The invasive front at the interface between the tumour and the non-lesional liver often remains viable when the centre of large lesions is necrotic and, therefore, unsuitable for the confirmation of diagnosis. Further, there may be research value in separate morphological assessment of the invasive tumour front with potential prognostic significance; for example, increased tumour budding at the invasive front has been shown to be associated with worse recurrence-free and overall survival.^45^ We recommend that specific sampling of the interface of the tumour with the non-lesional liver be undertaken.

17. *Peripheral liver with the closest overlying liver capsule should be sampled if the tumour is subjacent, overlying tissue is adherent, or there is macroscopic invasion of the liver capsule.*
18. *The gallbladder bed and wall should be sampled where the tumour is adjacent.*

We recommend sampling to allow microscopic assessment of areas that are macroscopically suspicious of capsular invasion. This permits formal demonstration of tumour invasion through the capsule into adherent extrahepatic structures. Perforation of the visceral peritoneum is classified as stage pT3 and invasion of extrahepatic structures is classified as stage pT4 by the TNM schema.^39^ In one study, visceral invasion was histologically confirmed in 45.7% of cases where there was surgical suspicion of invasion leading to visceral resection, and patients with histologically confirmed visceral invasion had a poorer prognosis, not associated with the operative procedure alone.^46^

19. *Areas suspicious for macrovascular invasion should be sampled.*

We recommend that areas macroscopically suspicious of vascular invasion be sampled. Vascular invasion is a feature of the TNM8 T-stage classification, defining pT2 classification in solitary tumours, and should be confirmed microscopically.

20. *Areas suspicious for bile duct invasion should be sampled.*

As noted, bile duct invasion is associated with a poorer prognosis and influences TNM classification. Areas suspicious for bile duct invasion should be sampled to allow this to be confirmed histologically.

21. *Any bile duct resection margin(s) should be sampled.*

Bile duct resection margins, where present, should be sampled to permit histological confirmation of either complete resection or involvement.

22. *Non-lesional liver distant from the tumour should be sampled.*

In resection specimens, we recommend that a block of non-lesional liver distant from the tumour be sampled, irrespective of the macroscopic impression. Histological assessment of non-lesional liver distant from the tumour allows diagnosis or confirmation of parenchymal disease that may influence the postoperative management of the patient. Further, this allows an interpretation of the tumour alongside possible underlying and contributory parenchymal or biliary disease.

Where a patient is undergoing a diagnostic lesional biopsy of suspected CCA, the requesting multidisciplinary team should guide the radiologist undertaking the biopsy on which samples to take, including requesting a biopsy of non-lesional liver tissue when the value of additional diagnostic information and management guidance outweighs the additional risk. The targeted lesional core and distant non-lesional core should be submitted to the pathology department as separate specimens so that they can be embedded separately, and any medical liver special stains only undertaken on the non-lesional block, preserving lesional tissue for molecular testing.

23. *An intratumoural block suitable for molecular testing should be taken or identified.*

Molecular testing is required to identify molecular alterations that define patient suitability for personalised therapies. Whilst those treatments currently licenced are only used in the setting of advanced disease, in resection cases we recommend identification of a tumour block at the time of specimen trimming that is suitable for testing should that be clinically indicated.

#### Block processing and routine tinctorial staining

24. *Five micron or thinner* *hematoxylin and eosin (* *H&E* *)* *-stained sections should be prepared from each block.*

Section thickness influences the staining characteristics of tinctorial and immunohistochemical staining^47^^,^^48^ and we recommend that sections no thicker than 5 μm be used. Although a trained pathologist is able to compensate for such variation, computational pathology methods that are in use in a research setting may be unduly influenced by staining variation as a consequence of section thickness.

#### Microscopic assessment

To aid interpretation of the histological appearance of a tumour by the reporting pathologist, we provide agreed guiding principles rather than explicit rigid recommendations.

25. *Intrahepatic cholangiocarcinoma is usually adenocarcinoma with identifiable glands. However, poorly-differentiated solid cholangiocarcinomas and rarer morphological subtypes (e.g. adenosquamous, squamous, mucinous, signet-ring, clear cell, mucoepidermoid, lymphoepithelioma-like, and sarcomatous) are described.*

Intrahepatic CCA is classically adenocarcinoma with the presence of identifiable glands. However, rarer alternative morphological subtypes are described in the World Health Organisation ‘blue book’ such that gland formation is not a *sine* *qua non* for a diagnosis of CCA^49^ and this diagnosis should always be considered in a biopsy from a liver lesion with no history of prior or synchronous extrahepatic malignancy.

26. *Desmoplastic stroma is often a prominent feature of intrahepatic cholangiocarcinoma. However, its absence does not preclude a diagnosis of cholangiocarcinoma as its distribution within a tumour may mean it is not present in needle biopsy material.*

Although prominent desmoplastic stroma is a common feature of iCCA, it is variably distributed within a tumour. The centre of a lesion is often more stroma-rich compared with the periphery that often includes more malignant epithelial elements.^50^ This necessarily means that the absence of desmoplastic stroma in a needle core biopsy does not preclude a diagnosis of CCA.

27. *Biliary intraepithelial neoplasia adjacent to an adenocarcinoma with desmoplastic stroma is highly suggestive of cholangiocarcinoma.*

On purely morphological grounds, iCCA (primary hepatic adenocarcinoma) cannot be distinguished from metastatic adenocarcinoma of an extrahepatic primary malignancy. CCA (extrahepatic or large duct type in particular) often develops through a multistep process with preceding dysplastic and *in situ* lesions. Where biliary epithelial neoplasia^51^ (BilIN) can be identified in association with invasive adenocarcinoma, more confidence can be ascribed to a diagnosis of CCA. However, in a study of resection cases, BilIN was only identified in 9.5% of cases with iCCA^52^ so its absence cannot exclude a diagnosis of CCA.

28. *Large duct-type intrahepatic cholangiocarcinoma is composed of columnar cells.*
29. *Cells of large duct-type intrahepatic cholangiocarcinoma are mucin-producing.*
30. *Cells of large duct-type intrahepatic cholangiocarcinoma are usually arranged in large malignant duct formations or have papillary architecture.*

Large duct-type iCCAs are composed of columnar cells forming large infiltrating glandular structures. In one in-depth morphological study, tumours classified as large duct-type on the cell and gland morphology contained extracellular mucin and intracellular mucin (by H&E staining) in 79% of cases.^53^

31. *Small duct-type intrahepatic cholangiocarcinomas usually show no or minimal mucin production.*
32. *Small duct-type intrahepatic cholangiocarcinomas have a predominant component of cuboidal to polygonal cells.*
33. *Small duct-type intrahepatic cholangiocarcinoma cells are predominantly arranged as small to intermediate-sized tubules and/or anastomosing glands.*

Small duct-type iCCA, in contrast, is composed of cuboidal cells arranged as small-to-intermediate sized tubules or anastomosing glands. Twenty-one percent of cases contained extracellular mucin, and 8% of cases contained intracellular mucin (by H&E staining).^53^

#### Immunohistochemistry

34. *No immunohistochemical pattern is specific for intrahepatic cholangiocarcinoma.*
35. *Distinction of intrahepatic cholangiocarcinoma from a liver metastasis of a primary upper gastrointestinal, pancreatic, or extrahepatic biliary tumour often cannot be made based on morphology and immunohistochemical profile.*
36. *Intrahepatic cholangiocarcinomas express typical pancreaticobiliary cytokeratins.*

The biliary epithelium has a developmental foregut origin in common with the upper gastrointestinal tract and pancreas. In keeping with this, iCCA typically expresses CK7 and CK19, in common with normal epithelium from those sites and tumours arising from them. This necessarily means that the immunohistochemical profile of these tumours is indistinguishable, there is no specific profile that is diagnostic of CCA,^54^ and that no routine immunohistochemical panels should be undertaken. This also serves to preserve tissue for molecular testing.

37. *Immunohistochemistry can help differentiate intrahepatic cholangiocarcinoma from hepatocellular carcinoma.*

Hepatocellular carcinoma (HCC) is typically immunopositive for HepPar1 and Arginase-1 and shows canalicular immunopositivity for CD10 and bile salt exporter protein. However, immunopositivity is often lost in those tumours whose cytological features are less classical, or in poorly differentiated tumours. Further, although HCC is usually immunonegative for CK7 and CK19, these cytokeratins are expressed in a minority of HCCs with likely worse prognosis. This means that the immunohistochemical profile may aid distinguishing iCCA from HCC but cannot provide absolute certainty.

#### Molecular testing

38. *Molecular testing, where available, including for FGFR abnormalities, IDH mutations, and any other abnormality with therapeutic significance should be undertaken.*

iCCAs have a greater rate of potentially actionable genetic abnormalities than many other tumours, evidenced by recent positive trials for patients whose tumours have *IDH1* mutations or *FGFR2* fusions, and we recommend that molecular testing for any abnormality with potential therapeutic significance be undertaken. Most clinical assays used for molecular testing use FFPE biopsy or resection material. Testing of FFPE material is increasingly reliable and convenient,^18^^,^^19^ allowing testing of archival cases without the need for rebiopsy to acquire fresh tissue. Molecular testing methods are rapidly evolving and include DNA- and RNA-based next-generation sequencing assays, immunohistochemistry, and fluorescence *in situ* hybridisation. Sequencing panels, assaying several targets simultaneously, are preferred to single gene assays as they provide more usable information and can also estimate tumour mutational burden. The complexity and variety of molecular tests may mean that different tests are undertaken by different laboratories; the ability to test FFPE material also means that samples can easily be sent to an outside laboratory for a test not undertaken locally. A complete discussion of molecular tests and their therapeutic rationale is available in the recent European Society for Medical Oncology (ESMO) Clinical Practice Guideline for biliary tract cancer.^55^

However, we do recognise that such testing is expensive and that targeted treatments are not currently recommended as a first-line option, so the timing and availability of testing will vary.

Given that a biopsy must now provide the information necessary for personalised treatment decisions in addition to a traditional diagnosis, the value of additional sections used for special tinctorial or immunohistochemical stains must be balanced with the requirement for sufficient material to remain available for molecular testing to guide therapy. Given the absence of any specific immunohistochemical profile that either defines or excludes a diagnosis of iCCA, the usefulness of the tissue for immunohistochemical testing compared with the value of using the same tissue for molecular testing favours the latter. Close collaboration between the reporting histopathologist and the molecular pathology laboratory will allow the most efficient and integrated use of the available tissue.

#### Written report and interpretation

39. *The American Joint Committee on Cancer/International Union Against Cancer (AJCC/UICCA) staging schema should be used for the reporting of resection specimens, and the stage should be included in the primary report.*

The 8th edition of the AJCC/UICCA TNM staging system for iCCA was published in 2017, modifying the 2010 7th edition. This staging system provides prognostic discrimination and is the most widely used although it may be further modified in time to incorporate additional prognostic factors.^56^ We recommend that this staging system be used. However, given recent data indicating that patients with stage II (TNM8) disease and hepatic metastases have poorer outcomes than those without, reporting the number of tumours will allow additional prognostic discrimination.^32^

40. *The results of any molecular analysis and immunohistochemical staining should be included in the primary report.*

Genetic alterations with associated personalised therapeutics may be identifiable in up to half of all iCCA,^57^ and the results of the molecular testing undertaken should be included in the primary histopathology report to guide treatment. Currently and depending on geographical location, personalised therapies are available for patients with iCCAs that have *FGFR2* fusions, *IDH1* mutations, *NTRK* fusions, *ERBB2* amplification, high microsatellite instability, and *BRAF* V600E mutations and the results of these tests should be reported. Sequencing panels often identify numerous variants of uncertain significance (VUS).^58^ Reporting of such variants is not consistently undertaken by molecular laboratories^59^ but there may be value in documentation of VUSs to allow continued data gathering that may lead to some recategorisation. There may also be clinical value in reporting the tumour mutational burden.^60^ If insufficient material is available or if testing of any type has failed, this should be documented to allow consideration of additional lesional sampling.

41. *The presence or absence of perineural invasion should be stated in the primary report.*

Perineural infiltration/invasion is a common histological finding in iCCA and should be reported. It may be associated with poorer prognosis.^61^^,^^62^

42. *The presence or absence of lymphovascular invasion should be stated in the primary report.*

Although lymphatic invasion is a precursor to lymph node metastasis, histologically-confirmed lymphovascular invasion is an indicator of poorer outcome even in patients without proven lymph node involvement^63^ and so should be reported.

43. *The presence or absence of dysplasia should be stated in the primary report.*

The presence of dysplasia at a bile duct resection margin may predict disease recurrence and should be reported.^64^

44. *Small-duct or large-duct designation of intrahepatic cholangiocarcinomas should be stated in the primary report.*

Although classification of iCCA as small duct or large duct-type is made on morphological grounds, the groups show different molecular alterations and, potentially, clinical features and this classification should be reported. The large duct-type has been shown to have a poorer prognosis.^65^

45. *A semiquantitative grading based on the proportion of glands should be stated in the primary report (well-differentiated, <95% of tumour composed of glands; moderately-differentiated, 50–95% of tumour composed of glands; poorly-differentiated, <49% of tumour composed of glands).*

Although there is not a well-established and standardised grading system for iCCA differentiation, the International Collaboration on Cancer Reporting reporting proforma suggests grading based on the proportion of the tumour showing gland formation.^5^ Data suggests that patients with less well-differentiated tumours have a poorer prognosis[66], [67], [68] and we recommend reporting differentiation based on the proportion of the tumour showing gland formation.

46. *The status of any resection margin(s) should be stated in the primary report.*

Incomplete resection is associated with poorer outcomes in patients undergoing potentially curative resection for iCCA,^33^ and so margin status and shortest distance between tumour and margin should be stated in the report.

47. *The status of any lymph nodes should be stated in the primary report.*

Patients with histologically proven lymph node metastasis have a poorer prognosis after resection than patients without lymph node spread.^30^^,^[69], [70], [71] The number of positive nodes and total number of nodes examined should be conveyed in the histopathology report of a resection specimen.

48. *Confident diagnosis requires the correlation of histomorphology and any immunohistochemical staining profile with available imaging and clinical information. Clinical history, endoscopic investigation, and imaging studies are all pivotal in reaching the correct conclusion.*

A number of consensus agreed statements indicate that a diagnosis of iCCA is not possible on the basis of histological features alone and, therefore, only possible by the integration and understanding of other clinical features provided by an array of non-pathologist members of the multidisciplinary team required for patient management.

## Discussion

The purpose of a pathology report is to provide information to the multidisciplinary team delivering patient care that allows them to offer the optimum treatment. These consensus recommendations provide guidance for all stages of the specimen pathway in the reporting of iCCA to maximise the informational value of the specimen and satisfy this purpose. By explicitly seeking consensus from those delivering patient care (end-users), the development process recognises that the authorised report must be used to have value. We have designed a template to help reporting pathologists, as fillable .pdf and editable .docx files (Supplementary Files 1 and 2).

The pathologist Delphi exercise was designed to reach as wide a geographical range of expert pathologists as possible by asking every national branch of the International Academy of Pathology to nominate a national expert. Pathologists from 28 countries with representation from Asia, Africa, Europe, North America, Oceania, and South America participated. Remarkably, consensus was reached rapidly without any variation related to geography. Further, those participating in the end-user part of the process, were all members of working group 4 (‘Epidemiology, clinical characterisation and trials’) of the EURO-CHOLANGIO-NET COST action,^72^ and also reached immediate consensus about the optimal content of the authorised pathology report both amongst themselves and with the pathologist participants.

**Fig. 1:**
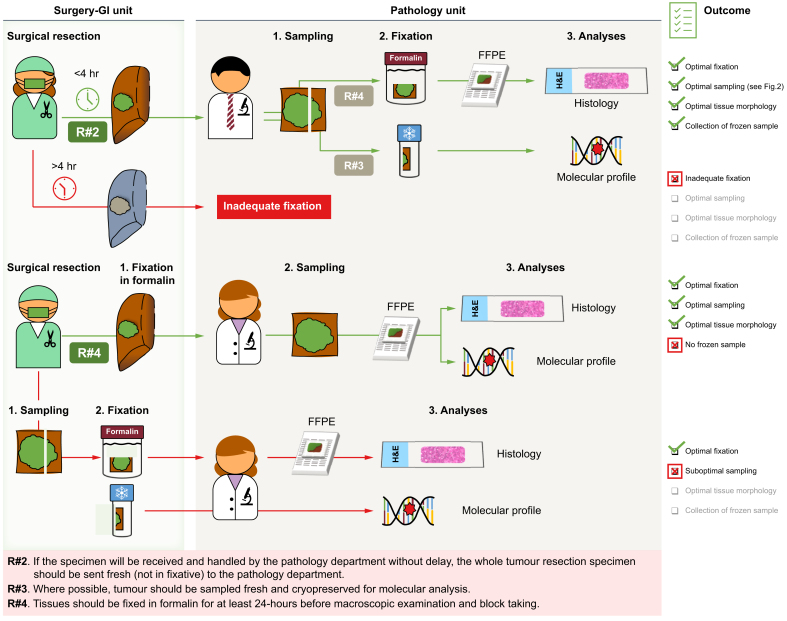
Schematic of the specimen pathway of a hepatic resection for diagnosis and management of intrahepatic cholangiocarcinoma. (FFPE, formalin-fixed, paraffin-embedded). Critical parts pf the specimen pathway for a resection are represented, with the numbers of the relevant recommendation statements corresponding to those in Table 1.

Personalised therapies for iCCA defined by the presence of specific molecular features of the tumour are now becoming available. Positive trials for FGFR2 inhibitors[73], [74], [75] and IDH1 inhibitors^76^ have led to FDA- and EMA-licensed treatments, and tumour agnostic therapies for *BRAF* V600E mutated,^77^ MSI-H,^78^ *NTRK*-fused,^79^ and *RET*-fused^80^ tumours are also FDA-licensed. Critically, treatment eligibility requires demonstration of an actionable mutation, and the ESMO guidance recommends the use of next-generation sequencing panels for CCA.^81^ Until such time as circulating tumour molecules can be reliably used to both diagnose the tumour and identify molecular abnormalities, cells and tissues submitted to pathology departments must provide a diagnosis and allow molecular characterisation (Fig. 1), and this may need a change in approach by reporting pathologists. The lack of a specific pattern of immunohistochemical staining for iCCA reduces the value of such staining and, critically, supports the statement (#48) that a confident diagnosis requires correlation with clinical and radiological features. By recognising the importance of clinicopathological correlation over immunohistochemistry, it follows that more tissue should be available in the diagnostic block for molecular testing to guide therapy.

**Fig. 2:**
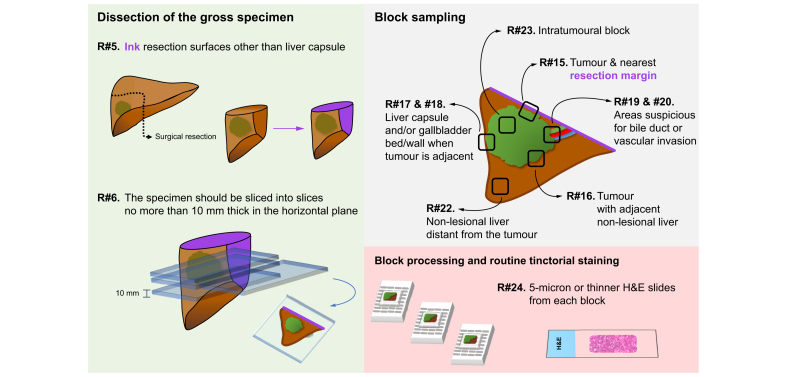
Schematic of dissection and sampling of a hepatic resection. (H&E, hematoxylin and eosin). Details of the macroscopic assessment, dissection, sampling, and associated slide preparation of the gross hepatic resection specimen, and associated recommendation statements, are indicated as in Table 1.

Many of the recommendations relate to activity that allows provision of accurate details about resection specimens (Fig. 2). Although this includes overall stage and features of tumour appearance and spread that have a strong evidence base of their prognostic value, the value of other features is unknown. Specimen handling and reporting of such features will allow their importance to be more systematically determined.

These recommendations have the limitation that only a self-selected group of highly-motivated pathologists and patient-facing clinicians were respondents and these may not be fully representative of those groups as a whole. However, the wide geographical spread of participants was chosen as an attempt to minimise this. We recognise that resources and facilities vary across territories so, wherever possible, recommendations reflect that variation and allow for local modification to suit.

We believe the recommendations in this report have incorporated and evaluated guidance developed and published by disparate national bodies in a transparent manner and represent a unified set of practice points that all pathologists reporting iCCA, wherever they are located, can follow to maximise the value of specimens for patient care through close and systematic communication between all professionals involved in the journey of the patients’ tissue sample.

## Financial support

No authors received financial support for the conduct of the research and/or preparation of the article.

## Authors’ contributions

Study concept and design: TK, MG, GC. Acquisition of data: TK, DO, MG, GC. Analysis and interpretation of data: all authors. Drafting of the manuscript: TK, DO, MG, GC. Critical revision of the manuscript for important intellectual content: JB, VC, EG, CB, BG. Supervision: TK, MG, GC. Final approval of the submitted version: all authors.

## Data availability statement

All data are available from the corresponding author on reasonable request.

## Conflicts of interest

TK undertakes consultancy work for Perspectum, Clinnovate Health, Kynos Therapeutics, Fibrofind, HistoIndex, Concept Life Sciences, and Resolution Therapeutics, and has received speaker’s fees from Incyte Corporation and Servier Laboratories. JB declares research grants (from Incyte, Albireo, Cymabay), personal fees for lectures (from Incyte, AstraZeneca), and consulting roles (for Albireo Pharma, AstraZeneca, Cymabay, Ikan Biotech and OWL-Rubió Metabolomics).

CB received honoraria as speaker (AstraZeneca, Incyte) and consultant (Incyte, Servier, Boehringer Ingelheim, AstraZeneca); received research funds (Avacta, Medannex, Servier) and her spouse is an employee of AstraZneca. CB is supported by a CSO research grant and CRUK Scotland Centre [grant number 100006]. VC received honoraria as speaker (Incyte, Albireo) and consultant (IPSEN); received research funds (Advanz). GC, DO, MG, BG, and EG have no conflicts of interest to declare.

Please refer to the accompanying ICMJE disclosure forms for further details.
