# Supplementary material for: Recommendations on maximising the clinical value of tissue in the management of patients with intrahepatic cholangiocarcinoma
Source: JHEP Rep. 2024 Mar 12;6(6):101067. doi: 10.1016/j.jhepr.2024.101067 (PMC11060959; doi:10.1016/j.jhepr.2024.101067)
Supplement: Multimedia component 1 [file mmc1.pdf]

Recommendations on maximising the clinical value of tissue in the management of patients with intrahepatic cholangiocarcinoma

Timothy Kendall, Diletta Overi, Maria Guido, Chiara Braconi, Jesus Banales, Vincenzo Cardinale, Eugenio Gaudio, Bas Groot Koerkamp, Guido Carpino

Table of contents

Supplementary Methods..2

Supplementary Text..5

Supplementary Figures...20

Supplementary Tables...23

Supplementary References...26

Supplementary Methods

Systematic review methods

The review question was developed based on the recommended Population, phenomena of Interest, Context framework - Population: Individuals involved in the histopathological reporting of iCCA, phenomena of interest: recommendations or guidelines specific to the histopathological reporting of iCCA, context: any part of the entire specimen pathway from receipt in the histopathology department to issue of the final report of an iCCA. The final review question was: What recommendations are there for the histopathological reporting of iCCA?

Protocol and registration

The systematic review protocol was prospectively registered on the Open Science Framework online repository[1].

Eligibility criteria

Resources were eligible for inclusion if they contained: 1) guidance (explicit guidelines or expert recommendations) and/or a checklist, which are (2) histopathological reporting-related, relevant to (3) iCCA, and (4) published in 1996 or later. There were no restrictions by language or publication type.

Information sources and search strategy

The search strategy was devised after examining the literature on MEDLINE and EMBASE as well as an internet search on Google Scholar to identify a comprehensive set of relevant search terms. The full search strategies for all database and web searches are available in the review protocol[1]. Keywords such as '(histolo*; OR patholo*)' AND '(checklist; OR guideline; OR checklist)' AND '(cholangiocarcinoma; OR bile duct cancer)' were used in the search through the databases of MEDLINE (Ovid), EMBASE (Ovid) and Cochrane Library. Web searches on Google and Google Scholar were performed using the advanced search function, with the keywords '(Pathology; OR Histology; OR Biomarkers)' AND '(Guideline; OR Checklist)' AND 'cholangiocarcinoma'. Only the first 3 pages (30 results) from each internet search were screened. The databases and search engines were searched from 1 January 1996 to 31 December 2021. Besides the year of publication, no other limitations were placed on the searches.

Selection of guidance resources

Titles and abstracts of the records were screened by two reviewers; TJK screened all the records and MG and GC acted as second reviewers. In cases where an abstract was not available, the full text of the article was retrieved. Resources which both reviewers selected for inclusion were subjected to full-text review, while any disagreements were arbitrated by a third reviewer (MG or GC). Two reviewers (TK and GC) independently reviewed the full-texts; any disagreements were discussed with a third reviewer (MG) and resolved by consensus.

Quality assessment of guidance resources

Each eligible resource was examined by two reviewers (TK and GC) using the Appraisal of Guidelines for Research & Evaluation Global Rating Scale (AGREE-GRS) quality assessment instrument[2]. Guidance resources were not excluded based on the quality scores. The scores were used to compare the variation in methodological quality across resources, which were categorised into low, moderate and high quality based on the AGREE-GRS scores as previously described[3].

Data extraction and data items

Using best practice from the JBI Manual for Evidence Synthesis[4], a pre-specified digital data extraction form was customised in Excel, adapting the JBI 'Text and opinion data extraction tool'[5] to incorporate data fields specific to systematic reviews of textual opinion-based evidence, as previously used[3]. The following data items were extracted from each resource:

- Context within specimen reporting pathway – areas of specimen reporting pathway the guidance related to.
- Phenomena of Interest – variables associated with histopathological iCCA reporting guidance (guidance development methodology, organisational and geographical representation in guidance development, clinical regulatory authority referenced, and presence of a reporting checklist).
- Verbatim extracts of guidance text.
- Interpretation of guidance statements; classifying guidance statements as 'explicit' or 'implicit' (explicit – 'distinctly expressing all that is meant; leaving nothing merely implied or suggested': implicit – 'suggested or understood but not directly expressed')[6].

Data from each resource were independently extracted by two reviewers (TK and GC) onto the data extraction form, followed by checks for consistency. Any discrepancies were discussed between the two reviewers and any disagreements resolved by a third reviewer (MG).

Data synthesis and analysis

The best fit framework synthesis method[7–9] that incorporates all elements of the JBI meta-aggregation approach[10,11], was used for synthesis and analysis of descriptive qualitative data. The five stages of this method require 'familiarisation' with the literature to select the appropriate *a priori* framework; the distinct stages of the specimen pathway between specimen receipt in a pathology department and final authorisation of the histopathology report were used. The core group (TK, MG, GC, DO) examined the thematically linked extracted guidance from each source and generated a unifying statement or statements that were considered to accurately capture the meaning of the extracted text.

International Delphi exercise

Ethical considerations

The UK Research Integrity Office's Code of Practice for Research and the Universities UK (UUK) Concordat to Support Research Integrity adopted as the University of Edinburgh's Research Integrity framework was followed and the project was assessed before study commencement using the UKRIO checklist for good practice in research in advance as a peer opinion study. Information was given to Delphi participants in the introductory page of the survey, and electronic informed consent, including for disclosure of participant name and affiliation at publication, was obtained following General Data Protection Regulation best practices and before survey completion (Supplementary Text 1).

Candidate item generation

The candidate items for the Delphi process were derived from the synthesised guidance generated by the systematic review. The final list was produced after discussion between the core group (TK, MG, GC, DO) allowing revision of, or addition to, the synthesised guidance to generate a complete offering to be assessed by the Delphi panellists. Statements were grouped into the stages of the reporting pathway between receipt of a specimen in the pathology department until the authorisation of the final pathology report.

Participants

Participants for the survey of histopathologists reporting iCCA were selected to include those practicing in multiple territories. The named secretary of every national division of the International Academy of Pathology were contacted to ask for a national expert. To democratise the process, and recent successes leveraging social networks of pathologists, an invitation to self-nominate was made on Twitter, amplified by liver pathologist experts on the network.

For the Delphi survey of 'end-users' (surgeons, oncologists, gastroenterologists) of histopathology reports, the membership list of working group 4 ('Epidemiology, clinical characterization and trials') of the EURO-CHOLANGIO-NET COST action was used.

Those responsible for developing the candidate items did not provide responses in the Delphi process to minimise potential bias.

Rating

Participants were asked to rate the importance of items on a nine-point Likert-type item scale, where '9' represented 'critical' and '1' represented 'not important'. Participants were able to use free-text boxes to comment about their scores, about the wording of the statement, or offer other items that were not included as candidates.

An item was defined as 'consensus-in' when >70% of participants scored the item as '7-9 (critical)' with <15% scoring '1-3 (not important)'. An item was classified as 'consensus-out' when >70% of participants scored an item '1-3' and <15% of participants scored it '7-9'. All other combinations were considered to be 'no consensus', in accordance with previous research that used similar nine-point scales[3].

'End-user' participants were only asked to rate statements regarding what should be included in the written report.

Procedure

A minimum of two rounds was chosen to allow participants to reflect and change scores between rounds. A third round was not required because complete agreement was reached by the end of the second round.

Communication between the researchers and Delphi participants used private, individual email addresses so participants remained anonymous to one another. The Delphi was conducted via Google Forms with links to the Delphi provided in the emails. In Round One (22nd September 2022), participants were given instructions, a consent form, and the items to rate. They were asked to complete the survey by 16th October 2022 and reminder emails were sent to non-responders.

Round Two (17th November 2022) invitations were sent only to those participants who completed Round One. Participants were advised which items did not reach consensus in Round One and how these had been amended based on feedback, and asked to re-rate these items. They were informed that their scores from Round One would be carried forward for any item for which a new score was not returned and asked to complete the survey by 2nd December 2022. Reminder emails were sent to non-responders.

Analysis

In Rounds One and Two, response rate, median score and item consensus scores were calculated. Data were analysed in R. In addition, free text comments were reviewed to determine if items needed amendment or additional items required inclusion.

Supplementary Text

Supplementary Text 1 Delphi consent statement

‘So that the process is transparent, we will include your name and affiliation in a list of participants to be published with the guidelines. By providing us with these details you are giving your consent for us to do this. We are also collecting other data about your experience but this information and participant responses to Delphi items will only be published in aggregated form, not attributed to individuals.

You are able to withdraw your consent at any time prior to submission of the guidelines for publication by contacting Dr Tim Kendall, University of Edinburgh (Tim.Kendall@ed.ac.uk).’

Supplementary Text 2 Verbatim extracted guidance

Bridgewater et al., 2014

4. Microscopic assessment

A pathological diagnosis of iCCA is based on the WHO classification of biliary tract cancer showing an adenocarcinoma or mucinous carcinoma. The most common histological findings of an iCCA are those of an adenocarcinoma showing tubular and/ or papillary structures and a variable fibrous stroma.

Although it has been proposed that the diagnosis of iCCA can be made based upon a combination of clinical presentation, laboratory analysis, and radiologic evaluation, pathological diagnosis is required for definitive diagnosis in most patients, particularly those with cirrhosis and small hepatic mass lesions as radiographic studies are nonspecific.

Pathological diagnosis is recommended for all patients who will be undergoing systemic chemotherapy or radiation therapy, or enrolling in a therapeutic clinical trial. The sensitivity of liver biopsy for pathological diagnosis will depend upon location, size, and operator expertise. Core biopsies are required for definitive diagnosis. Although a positive liver biopsy will establish a diagnosis, a negative biopsy does not exclude it because of the potential for sampling error.

5. Immunohistochemistry

iCCA needs to be distinguished from benign biliary lesions such as peribiliary glands, reactive ductular proliferation, biliary microhamartomas (von Meyenburg complexes) and bile duct adenomas (peribiliary tract hamartomas), particularly in the presence of inflammation which can result in reactive cellular atypia. The histological appearance of iCCA is similar to that of metastatic adenocarcinoma arising from extrahepatic primary tumors and especially those of foregut origin such as lung, pancreas, esophagus, and stomach. The differentiation of iCCA from metastatic adenocarcinoma cannot be readily ascertained on histological examination. Differentiation between iCCA and mixed HCC tumors may require evaluation of specific markers of hepatocellular or progenitor cell features such as Hep-Par-1, GPC3, HSP70, glutamine synthetase, EpCAM, and CK19. CK19 positivity is not specific for iCCA. The expression of cytokeratin 7 and cytokeratin 20 may be helpful to establish a biliary origin.

7. Written report

The 7th edition of the AJCC/UICCA staging schema is currently the preferred staging system for resected iCCA.

Burt et al., 2020

2. Macroscopy/cut up/trimming of the gross specimen

When previous therapy has been administered microscopic examination of the entire tumour should be done when feasible. For selective sampling, sampling an entire cross section has been recommended if the tumour is <2 centimetres (cm) with an additional section for each 1 cm for larger tumours.

Additional sampling of areas that appear grossly viable is often necessary.

The following guidelines are provided for intrahepatic tumours:

- Tumour with nearest hepatic resection margin (when this is close enough to the tumour to be included in the block).
- Other blocks of tumour with adjacent liver tissue (for microscopic vascular invasion (MiVI)).
- Liver capsule if there is a possibility of capsular invasion, i.e., where there is subjacent tumour and overlying adherent tissue or macroscopic capsular invasion. Where the capsule appears intact over subcapsular tumour, with a smooth shiny surface, histology is not required to confirm capsular integrity.
- Gallbladder bed and wall where there is adjacent intrahepatic tumour.
- Any site macroscopically suggestive of macrovascular or bile duct invasion.
- Background liver (taken as far away as possible from the tumour).

A block of representative background liver should be taken at a distance from the tumour, whether or not it looks abnormal macroscopically.

The number of tumours is also recognized as an important prognostic factor in intrahepatic cholangiocarcinoma. Location of all tumours (HCC and intrahepatic cholangiocarcinoma) should be reported since this is important for correlation with imaging when this is available.

Distinction from metastatic adenocarcinoma is based on the presence of a single or dominant intrahepatic mass and absence of a known extra-hepatic primary tumour.

4. Microscopic assessment

According to the current WHO Classification 5th edition, the two main histological subtypes of intrahepatic CC are the large duct type, arising in the intrahepatic large ducts and composed of mucin secreting tumour cells and the small duct type (non-mucin secreting and mainly occurring in the hepatic periphery).

Cholangiolocarcinoma (CLC) and intrahepatic cholangiocarcinoma with ductal plate malformation pattern are subtypes of small duct intrahepatic CC.

Four tumour growth patterns of intrahepatic cholangiocarcinoma are described: the mass-forming type, the periductal infiltrating type, the intraductal growth type and the mixed type. Mass-forming intrahepatic cholangiocarcinoma (65% of cases) forms a well-demarcated nodule growing in a radial pattern and invading the adjacent liver parenchyma. The periductal-infiltrating type of cholangiocarcinoma (6% of cases) spreads in a diffuse longitudinal growth pattern along the bile duct, and the intra-ductal growth type (4% of cases) shows a polypoid or papillary tumour within the dilated bile duct lumen.

Definitive criteria for histological grading of cholangiocarcinomas have not been established; however, the following semiquantitative grading system based on the proportion of gland formation within the tumour is commonly used for intrahepatic cholangiocarcinomas:

- Well differentiated (more than 95% of tumour composed of glands)
- Moderately differentiated (50% to 95% of tumour composed of glands)
- Poorly differentiated (up to 49% of tumour composed of glands)

5. Immunohistochemistry
Immunohistochemical markers that may be used to demonstrate hepatocellular differentiation in poorly differentiated tumours include arginase-1, Hep Par 1, pCEA, CD10, BSEP, AFP and glypican-3.

7. Written report
Record extent of invasion.

Record perineural invasion.

Record lymphovascular invasion.

Record other pathologies.

For dysplasia involving large bile duct radicles we recommend the use of the BilIN and Intraductal papillary neoplasm of the bile ducts (IPNB) classifications described in the WHO 5th edition guidelines, both of which distinguish low grade from high grade change.

Margin status - For intrahepatic cholangiocarcinoma there are a few publications citing margin status as a prognostic factor on multivariate analysis. A systematic review of intrahepatic CC did not include margin status among significant prognostic factors.

Lymph node metastases in intrahepatic and perihilar cholangiocarcinoma have been identified as an important predictor of prognosis.

Cong et al., 2016

1. Specimen fixation
The surgeons should fill an Application Form of Pathological Examination describing the clinical diagnosis, location and type of lesions and number of tissues. The surgical margin, suspected lesions, important vessels and bile duct margin should be marked with a dye or suture by surgeons. Small resected tissues, such as lymph nodes, should be placed into different containers and labeled with corresponding descriptions.

To maximally preserve the integrity of intracellular nucleic acids and proteins for avoiding autocytolysis, tumor specimens should be transferred to the Department of Pathology as soon as possible after resection, ideally within 30 min after surgical removal for sectioning and fixation.

The fresh specimens should be cut consecutively into 1cm thick multiple sections at the maximal diameter; a portion should remain unfixed fresh or cryopreserved for molecular examination.

At room temperature, tissues should be fixed in a neutral formalin solution (v:v, 1:4-5) for 12-24 h and embedded in paraffin.

2. Macroscopy/cut up/trimming of the gross specimen
Hepatic tumor samples should be collected using the 7 point baseline sampling protocol

The location and number of liver tissues collected should be determined as appropriate according to the size, shape and number of the liver tumors as well as the adjacent liver tissues.

Because the detection rate of MVI and satellite nodules is related to the extent of adjacent liver tissues, it is necessary to describe the size of the adjacent liver tissues, and the suspected lesions should be sampled after reviewing several sections.

When the tumor tissue is close to the surgical margin, sampling should be done at the region vertical to the margin closest to the cancer. When the tumor tissue is far away from the surgical margin, sampling should be done parallel to the surgical margin. The status of the surgical margin should be determined using the section with maximal area.

3. Block processing and routine tinctorial staining

Sections of 5µm thickness should be cut from each block and stained with hematoxylin and eosin for histological examination.

4. Microscopic assessment

Adenocarcinoma is the most common histological type of ICC, although it may also present in other special histological and cell types and its differentiation degree can be classified as well, intermediate and poor.

Tumor growth patterns, including peritumoral invasion, capsule invasion, MVI and satellite nodules.

The presence of chronic liver disease, such as chronic hepatitis or hepatic cirrhosis. Although there are many systems for grading and staging chronic viral hepatitis, a simple histologic scoring system is recommended for routine pathological diagnosis, such as the Scheuer scoring system, etc. Furthermore, Masson's trichrome staining and reticular fiber staining can be routinely undertaken to assess the degrees of hepatic fibrosis and lobule reconstruction, respectively.

5. Immunohistochemistry

Currently used biomarkers for liver cancer are somewhat imperfect in their diagnostic specificity and sensitivity; thus, a biomarker panel in combination with other tissue-specific markers could represent a useful tool for diagnosis and differential diagnosis between benign and malignant hepatocellular tumors, HCC and ICC, other specific types of hepatic tumors, and primary and metastatic liver cancer.

Although immunohistochemical staining for CD34 does not directly label hepatic parenchymal cells, it is valuable for determining the extent of MVD and examining its unique distribution pattern in different liver tumors. For instance, a diffuse staining pattern is indicative for HCC, a scattered staining pattern for ICC, a patchy staining pattern for HCA, and a cord-like staining pattern for focal nodular hyperplasia, etc.

Fernandez Moro et al., 2016

5. Immunohistochemistry

A combination of “generic” adenocarcinomamarkers (CK7, CK19, BerEP4, and polyclonal CEA) together with vimentin and a limited number of “pancreatobiliary” markers (MUC1 and CA19-9) defines its characteristic immunohistochemical profile. The present study identified cytoplasmic WT1 as a novel marker for intrahepatic cholangiocarcinoma. The characteristic immunohistochemical profile of intrahepatic cholangiocarcinoma positively supports its pathological diagnosis, which no longer needs to be regarded as a diagnosis of exclusion (of metastatic adenocarcinoma).

Geller et al., 2008

4. Microscopic assessment

Cholangiocarcinoma most often must be differentiated from metastatic carcinoma. Cholangiocarcinoma is almost always a desmoplastic tumor and often is mucin producing.

5. Immunohistochemistry

The immunophenotype of CCA is generally similar to that of extrahepatic pancreaticobiliary carcinomas. Almost all CCAs react with CK7, CK19, CEA (both monoclonal and polyclonal; noncanalicular pattern), and MOC-31. In contrast to other pancreaticobiliary carcinomas, CCAs are more likely to react with CK7 and less likely to react with CK17, CK20, and p53. Cytokeratins can also be useful in differentiating HCC from CCA, as can thyroid transcription factor 1 and claudins. Cholangiocarcinoma can also react with CA 125, and differentiation from mullerian carcinomas can be difficult, although CCA is almost never estrogen receptor positive. Newer studies with the mucins (eg, MUC4, MUC5AC, MUC5B, MUC6) suggest that they may be useful in the subclassification of CCAs and may be useful in evaluating prognosis.

Jiang et al., 2018

2. Macroscopy/cut up/trimming of the gross specimen

Pathologic staging depends on pathologic documentation of the anatomic extent of disease, whether the primary tumor has been completely removed. If a biopsied tumor is not resected for any reason and if the highest T and N categories or the M1 category of the tumor can be confirmed microscopically, the criteria for pathologic classification and staging have been satisfied without complete removal of the primary cancer. The AJCC eight edition classifies the T category according to vascular invasion, extent of invasion, and the number of tumors.

4. Microscopic assessment

Compared to other types of cancer involving liver, cholangiocarcinomas are the ones that elicit a marked desmoplastic fibrotic reaction.

It has been well recognized that cholangiocarcinomas demonstrate a wide spectrum of growth patterns. Tumors can be composed of irregular cystic, branching/glandular tubular structures or as irregular aggregates of infiltrating glands.

Cholangiocarcinomas do not always show easily identifiable tubular contours; instead, tumor cells often grow in a nodular pattern or form sheets of solid nests that mimic neuroendocrine neo-plasms and/or HCC. Other tumors may show copious mucin components, resembling mucinous adenocarcinoma or colloid tumors.

It should be kept in mind that for any cholangiocarcinoma, it is common to see a variety of the abovementioned histomorphological features in the same lesion.

Focal features of sarcomatoid or clear cell morphology are not uncommon.

Cholangiocarcinoma tumor cells frequently extend along the portal tracts by growing within the connective tissue, without directly invading into the bile ducts in the vicinity, even though this pattern of tumor invasion can be seen in not only cholangiocarcinoma but also other types of tumors, including metastatic carcinomas. Also, tumor cells can colonize and extend along the bile ducts and intermingle with native benign bile ducts and adjacent reactive ductules.

It should always be kept in mind that cholangiocarcinomas are neurotropic, and tumor cells frequently approach nerve tracts, with frequent perineural and intraneural involvement. In practice,

this pattern can be very challenging to recognize, especially during frozen section evaluation; more so when the primary tumor is small and when there is no clinical evidence of metastatic disease.

Another malignant behavior of cholangiocarcinomas is lymphovascular invasion. Astute microscopic examination and extensive tumor sectioning is essential in reaching such a diagnosis.

The usual intraluminal “dirty” necrosis, frequently associated with colorectal primary adenocarcinomas, are not typically seen in cholangiocarcinomas.

Histologically, cholangiocarcinoma follows a stepwise carcinogenesis process through a precursor lesion: BilIN, ranging from low to high grade (carcinoma in situ). The presence of these precursor lesions in the vicinity of an intrahepatic adenocarcinoma, combined with marked desmoplastic reaction and high-grade cytological atypia, is highly suggestive of cholangiocarcinoma, instead of metastatic colorectal adenocarcinoma; the latter usually demonstrates palisading, pencil-like nuclei and intraluminal “dirty” necrosis. However, a frequent obstacle encountered in practice is the small size of the biopsy material, which inevitably restricts the differential list and limits reaching a definitive diagnosis due to small areas of useful diagnostic material.

5. Immunohistochemistry

By immunostaining, cholangiocarcinomas are strongly positive for CK-7 and CAM5.2 and show cytoplasmic labeling by p-CEA. Mouse monoclonal antibody that recognizes human EPCAM on cell membrane (MOC31) is positive in around 90% of cases, whereas CK-19 is positive in 70% to 80% of cases. Interestingly, there have been findings to suggest that peripheral cholangiocarcinomas are more likely to be CK-7 positive but CK-20 negative, whereas central counterparts tend to be positive for both CK-7 and CK-20. CD56 also is reported to be positive in peripheral cholangiocarcinomas, which does not necessarily mean there is neuroendocrine differentiation, if other neuroendocrine markers are negative.

One particularly useful immunostain marker for confirming cholangiocarcinoma is S100P, which is negative in benign biliary epithelium but usually positive in cholangiocarcinomas, especially when combined with U3 small nucleolar ribonucleoprotein protein (IMP3) and a protein that in humans is encoded by the von Hippel -Lindau tumor suppressor gene (pVHL). In comparison, HCCs, including all the variants, are predominantly positive in the described pattern for the immunostain markers introduced earlier, including arginase, HepPar-1, glypican 3, CD34, CD10, and p-CEA; HCCs are also positive for AE1/3 and CAM5.2 in almost all cases but remain negative mostly for CK-7 and CK-20. In contrast, cholangiocarcinomas are mostly negative for the markers for HCC, except for p- CEA, which could be positive in various adenocarcinomas, irrespective of the organs of origin.

Rarely, HepPar-1 could be positive in cholangiocarcinoma cases, with foci of labeling, but given the negativity by other HCC markers and adenocarcinoma histomorphology and immunoprofiles, a diagnosis of cholangiocarcinoma should be beyond the question; this occasional cellular labeling is not specific.

In addition to HCC, a metastatic process from another organ or site is also frequently suspected when facing a potential cholangiocarcinoma, such as tumors originating in gastrointestinal luminal sites, especially those from colorectal regions.

It should be made clear to the entire clinical team that there are no specific markers that can be dependable for diagnosing cholangiocarcinoma. Cholangiocarcinomas are usually positive for CK-7, and sometimes for CK- 20, similar to upper gastrointestinal tract and lung adenocarcinomas. A positive Thyroid Transcription Factor 1 (TTF-1) or napsin-A immunostain would strongly favor

pulmonary origin; however, occasional lung tumors are negative for TTF-1 or napsin-A by immunostaining; these cases require detailed histomorphological evaluation and systemic radiological and clinical correlation to narrow down the most likely origin of the tumor. Likewise, the positive CK-20 labeling and/or focal CDX-2 immunoreactivity does not necessarily indicate gastrointestinal tract or colorectal origin.

In practice, immunohistochemical studies (such as cytokeratin-7 (CK-7), cytokeratin-19 (CK-19), cytokeratin-19 (CK-20), CDX-2, and carbohydrate antigen 19-9 (CA-19-9), in an attempt to tell one potential primary source from another one. However, none of these immunophenotypes are specific for telling cholangiocarcinoma; they are just frequently seen from pancreatobiliary and cholangiolar primaries.

Subsequently, it is extremely important to correlate radiological, clinical, and histopathological findings in order to draw a correct conclusion.

7. Written report

The pathological diagnosis of primary intrahepatic cholangiocarcinoma still mostly remains one of exclusion, because of a lack of specific markers. However, a novel RNA platform using in-situ hybridization for albumin RNA has shown specificity for primary liver cancers, including CC.80 However, since this test is not widely available, diagnosis of primary intrahepatic cholangiocarcinoma still requires exclusion of all other other possibilities by analyzing histomorphology, immunoprofiles, imaging studies, as well as clinical evidence. Clinical history, endoscopic investigation, and ima- ging studies are all pivotal in reaching the correct conclusion.

Frequently, making a diagnosis of cholangiocarcinoma especially the intrahepatic ones, instead of a metastatic adenocarcinoma from another organ or site, can be difficult and challenging, more so when there are none or limited clinical and radiological information available for analysis. The clinical, histological, and molecular information are all essential for decision-making and management planning.

Clinically, cholangiocarcinomas are associated with chronic biliary inflammation and regeneration, as frequently seen in PSC, fluke infestation, chronic hepatitis C and hepatitis B viral infection, profession-related chronic exposure to chemicals, and pancreatobiliary malfunction. On the other hand, history of colorectal, gastrointestinal, or gynecological malignancies as well their stages are helpful in determining the possibility of dealing with a metastatic tumor.

It is very important to compare the histomorphology of the primaries and the adenocarcinoma in the liver to see whether there is any similarities; unfortunately in the setting of a comprehensive cancer center, it is not unusual to face a newly identified intrahepatic adenocarcinoma with no or minimal history and no previous cases for morphological comparison.

To summarize briefly, in order to decide whether an intra-hepatic adenocarcinoma is a primary cholangiocarcinoma or a metastatic process, morphological and ancillary test results need to be utilized in combination with the clinical and radiological findings to reach the most likely conclusion regarding the true nature of the tumor.

Khan et al., 2012

2. Macroscopy/cut up/trimming of the gross specimen

Macroscopic features of intrahepatic CC Intrahepatic CCs are whiter and firmer than HCCs as they contain more desmoplastic stroma. They occur more commonly in non-cirrhotic livers than HCCs and are divided into four macroscopic types.

4. Microscopic assessment

Over 90% of CCs are adenocarcinomas and are classified according to the percentage of tumour composed of glandular tissue. Some types of adenocarcinoma are not graded (eg, carcinoma in situ, clear cell adenocarcinoma and papillary adenocarcinoma). Signet ring cell carcinoma is graded as 3 and small cell carcinoma as 4.

Tumours are usually adenocarcinomas and have prominent desmoplastic stroma. However, except in cases where there is co-existing biliary dysplasia, it may not be possible, even with immunohistochemistry, to differentiate between CC and metastatic tumour. Examples of this include intraductal papillary neoplasm with associated invasive neoplasia, and mucinous cystic neoplasm with associated invasive neoplasia.

5. Immunohistochemistry

Distinguishing intrahepatic CC from metastatic adenocarcinoma and other primary liver tumours can be difficult. Accurate differentiation, particularly from foregut metastases (lung, oesophagus, stomach, pancreas), often cannot be made histologically. Other modalities, especially imaging, are essential.

Immunohistochemistry panels including CK7, CK19, CK20, CDX-2, TTF-1, oestrogen/progesterone receptors and PSA, depending on clinical context, can be helpful. CCs are usually CK7 positive and CK20 negative.

In distinguishing HCC from CC, lack of mucin production and expression of HepPar-1, CD10 and glypican-3 by HCC are useful.

7. Written report

CC staging is based on the tumour-node-metastasis (TNM) system. The 7th edition of the TNM classification introduced a specific staging system for intrahepatic CC, separate from HCC, providing better prognostic information.

Surgical resection specimens should be reported systematically, for example, according to Royal College of Pathologists' guide- lines. The final report should include the following information:

Tumour - a. Histological type b. Histological grade c. Extent of invasion (according to the TNM system) d. Blood/lymphatic vessel invasion e. Perineural invasion: this is common and has prognostic significance.

Margins - These must be adequately sampled because local recurrence is related to involvement of the margins. This is particularly important because extrahepatic CC may be multifocal in up to 5% of cases.

Regional lymph nodes - To stage lymph nodes accurately, the node groups must be specifically identified. Peripancreatic nodes located along the body and tail of the pancreas are considered sites of distant metastasis.

Additional pathological findings - These must be noted if present (eg, carcinoma in situ, sclerosing cholangitis).

Metastases - Metastases to other organs or structures should be reported.

Krampitz et al., 2019

4. Microscopic assessment

Typically, pathologic analysis of biopsy specimens reveals adenocarcinoma, and the challenge is differentiating primary intrahepatic cholangiocarcinoma from metastasis from other gastrointestinal or pancreatic primary tumors, often requiring additional immunohistochemical evaluation.

7. Written report

Recognition of the biological and epidemiologic differences between hepatocellular carcinoma and intrahepatic cholangiocarcinoma led to the development of a distinct tumor, node, metastasis (TNM) staging system for intrahepatic cholangiocarcinoma that has been updated in the Eighth Edition of the American Joint Commission on Cancer (AJCC) Cancer Staging Manual.

Miyazaki et al., 2015

2. Macroscopy/cut up/trimming of the gross specimen

Since the ductal margin status has an im- pact on patients' prognosis, the histological assessment of the ductal resection margin should be performed intraoperatively.

4. Microscopic assessment

Biliary intraepithelial neoplasia (BilIN) and intraductal papillary neoplasm of bile duct (IPNB) are premalignant lesions for bile duct cancer.

Washington et al., 2010

2. Macroscopy/cut up/trimming of the gross specimen

Sections should be prepared from each major tumor nodule, with representative sampling of smaller nodules if macroscopically different in appearance

The evaluation of margins for total or partial hepatectomy specimens depends on the method and extent of resection. It is recommended that the surgeon be consulted to determine the critical foci within the margins that require microscopic evaluation. The transection margin of a partial hepatectomy may be large, rendering it impractical for complete examination. In this setting, grossly positive margins should be microscopically confirmed and documented. If the margins are grossly free of tumor, judicious sampling of the cut surface in the region closest to the nearest identified tumor nodule is indicated. In selected cases, adequate random sampling of the cut surface may be sufficient. outcome for intrahepatic cholangiocarcinoma.

Histologic examination of a regional lymphadenectomy specimen usually involves examination of 3 or more lymph nodes.

4. Microscopic assessment

Combined or mixed hepatocellular-cholangiocarcinoma accounts for less than 5% of primary liver carcinomas and should show histologic evidence of both hepatocellular differentiation and bile duct differentiation, such as production of mucin.

A system based on the proportion of gland formation within the tumor is suggested:

- Grade X Grade cannot be assessed
- Grade 1 Well differentiated (more than 95% of tumor composed of glands)
- Grade 2 Moderately differentiated (50%–95% of tumor composed of glands)
- Grade 3 Poorly differentiated (5%–49% of tumor composed of glands)
- Grade 4 Undifferentiated (less than 5% of tumor composed of glands)

Three tumor growth patterns of intrahepatic cholangiocarcinoma are described: the mass-forming type, the periductal-infiltrating type, and mixed mass-forming/periductal-infiltrating type. Mass-forming intrahepatic cholangiocarcinoma (60% of cases) forms a well-demarcated nodule growing in a radial pattern and invading the adjacent liver parenchyma (Figure 2). In contrast, the periductal-infiltrating type of cholangiocarcinoma (20% of cases) spreads in a diffuse longitudinal growth pattern along the bile duct. The remaining 20% of cases of intrahepatic cholangiocarcinoma grow in a mixed mass-forming/periductal-infiltrating pattern.

7. Written report

The histologic examination of the bile ducts at the cut margin is recommended to evaluate the lining epithelium for in situ carcinoma or dysplasia. If the neoplasm is found near the surgical margin, the distance from the margin should be reported. For multiple tumors, the distance from the nearest tumor should be reported.

The TNM staging system of the American Joint Committee on Cancer and the International Union Against Cancer applies to all primary carcinomas of the intrahepatic bile ducts and mixed hepatocellular-cholangiocarcinomas. It does not apply to hepatic sarcomas or to metastatic tumors of the liver.

Wyatt et al., 2012

2. Macroscopy/cut up/trimming of the gross specimen

Specimens can be dissected in the fresh or fixed state. Although formalin penetrates the liver poorly, intrahepatic tumours are usually clearly demarcated within the liver and examination after 24–48 hours does not significantly impair morphology.

Specimen hardening following fixation facilitates accurate slicing.

If fresh tumour is required, this can be obtained either by slicing the specimen fresh after painting the resection margin, or (if identifiable from the external appearance) by excising a portion of tumour through the capsule, so long as the capsule appears intact and is not covered by adherent fatty tissue which may result from underlying capsular breach by the tumour. The surfaces of the specimen other than the capsule (i.e. parenchymal resection plane, extrahepatic biliary tree, any tissue adherent to the liver capsule) should be painted with ink or silver nitrate to allow identification in histological sections.

Block-taking strategy for all liver specimens:

- Tumour with nearest hepatic resection margin (when this is close enough to the tumour to be included in the block).
- Other blocks of tumour with adjacent liver tissue (for microscopic vascular invasion).
- Liver capsule if there is a possibility of capsular invasion, i.e. where there is subjacent tumour and overlying adherent tissue or macroscopic capsular invasion. Where the capsule appears intact over subcapsular tumour, with a smooth shiny surface, histology is not required to confirm capsular integrity.
- Gall bladder bed where there is adjacent intrahepatic tumour.
- Any site macroscopically suggestive of vascular or bile duct invasion. Background liver (taken as far away as possible from the tumour).

The number of tumour blocks will depend on the tumour type, but should include samples from areas of differing macroscopic appearance in heterogeneous tumours.

A block of representative background liver should be taken, whether or not it looks abnormal macroscopically. The appearance should be included in the text description of the specimen, but is a core item in the dataset proforma only in the histology section, since microscopy provides the more reliable assessment of fibrosis/cirrhosis.

The following additional blocks are required as appropriate:

- where there is tumour tissue close to the hepatic hilum, the hilum should be sampled to include large vessels. Specifically label blocks of main left or right portal vein or bile duct, if present hepatic vein margin (if there is tumour nearby) extrahepatic biliary tree (when included)
- gall bladder – optional when this is macroscopically normal

The site of lymph nodes should be specified if known (hilar, hepatic artery, portal vein, cystic duct). More distant nodes (coeliac axis, periduodenal, periaortic) may be submitted separately by the surgeon. Large nodes that do not show macroscopic involvement should be serially sliced and embedded in their entirety since nodes at this site are often enlarged as a result of reactive changes and may harbour micrometastases, especially in cholangiocarcinoma.

Record the segments resected and the specimen weight after opening the gall bladder and rinsing out the bile.

The specimen dimensions (antero-posterior, medio-lateral and supero- inferior) should also be measured, particularly in cases where much of the specimen is occupied by tumour.

When included with the specimen, record the length of extrahepatic duct, number and site of lymph nodes, size and appearance of gall bladder.

The specimen should be sliced at right angles to the parenchymal resection plane, and preferably in the horizontal plane to facilitate correlation with preoperative cross-sectional imaging. Slices should be as thin as possible, and no more than 10 mm thick. The minimum size of tumours detectable by imaging is now less than 5 mm.

Record the number, site, maximum diameter and distance from hepatic margin of the tumour(s). For multiple tumours, the sites should be recorded in the text of the report in such a way that allows correlation with preoperative imaging. For example, this can conveniently be recorded by numbering the horizontal slices from the top, and specifying the slices and approximate segments of each tumour. ‘Multiple tumours’ encompasses satellitosis, multifocal tumours and intrahepatic metastases. The presence of satellite nodules should be noted.

The appearance of the background liver (normal, bile-stained, fibrotic/cirrhotic) should be recorded. It is good practice to keep a photographic record of the macroscopic features of the specimen for use during MDT meetings.

The specimen should be inspected carefully for macroscopically apparent vascular invasion, and any suspected vascular invasion should be sampled for histological confirmation. Involvement of the main left or right branch of the portal vein or any of the three main hepatic veins should be specifically recorded, as this information is relevant to TNM staging.

The type of intrahepatic tumour may not be known until histology, however the same core items are required for all:

- type of specimen

- specimen weight
- specimen dimensions (where orientation is known, provide antero-posterior, medio-lateral and superior-inferior dimensions)
- tumour number and size
- presence of satellite lesions (regarded as multiple tumours for TNM staging)
- distance from nearest hepatic resection margin(s)
- for HCC and intrahepatic CC: macroscopic involvement of vessels; specify if main left or right portal vein or a main hepatic vein, and record diameter of vessel involved
- integrity of liver capsule (including bare area on postero-superior aspect) and presence of adherent tissues (e.g. diaphragm) or other organs
- presence and number of lymph nodes received.

3. Block processing and routine tinctorial staining

Needle biopsy - Sectioning and staining - Initially one or two shallow levels stained with H&E should be examined. The pathologist can then determine whether tumour is present and what further investigations are required based on the morphology of the tumour in the biopsy and clinical circumstances.

4. Microscopic assessment

Provide comment on:

- Tumour type
- Tumour differentiation
- Minimum distance to resection margin (hepatic, and where appropriate bile duct or vascular) measured microscopically when less than 5 mm. Microscopic involvement (R1) is generally defined as a clearance of <1 mm.
- Invasion through liver capsule (Glisson's capsule)
- Vascular invasion including confirmation of macroscopic vessel invasion. ? Perineural invasion (cholangiocarcinoma). ? Effects of ablative or neoadjuvant therapy on tumour (if applicable).
- Background liver – presence and stage of fibrosis, and other chronic liver.
- Lymph node involvement (where appropriate); number of nodes with metastasis.

In addition to the location of the CC, the Japanese classification according to the growth pattern into mass-forming, periductal infiltrating and intraductal papillary has gained international recognition. In general, mass-forming CC arises peripherally in the liver. The periductal infiltrating pattern is characteristic of perihilar CC, which includes CC arising in right, left and common hepatic ducts.

Intrahepatic CCs form an expansile tumour mass with obvious borders, and usually arise peripherally in the liver. The maximum diameter is readily determined. The size of the tumour is not a staging criterion since prognosis is independent of size however, it is important for correlation with preoperative imaging.

Staging depends on the number of tumours (single or multiple) and vascular invasion. Unlike HCC, vascular invasion (any size of vessel, pT2a) is distinguished from multiple tumours (pT2b, with or without vascular invasion). Tumour perforating the visceral peritoneum or direct infiltration of adjacent organs constitutes pT3 disease. The periductal infiltrating pattern, when associated with a tumour mass, indicates late-stage disease with a poorer prognosis and is classified as pT4 in TNM7. If the tumour is entirely of periductal infiltrating pattern, consideration should be given as to whether it arises in a main right or left duct; if so, it should be staged as perihilar CC.

Intrahepatic CC typically has a microacinar glandular pattern with central sclerosis, and distinction from metastatic adenocarcinoma particularly from stomach or pancreas is based on the single or dominant intrahepatic mass and absence of a known extra-hepatic primary tumour. Currently, available immunohistochemistry is not contributory.

Most intrahepatic CCs are adenocarcinomas. Rare variants listed in the WHO classification include adenosquamous, squamous, mucinous, signet ring, clear cell, mucoepidermoid, lymphoepithelioma-like (EBV associated) and sarcomatous intrahepatic CCs.

Locally advanced intrahepatic mass-forming CCs invade through the liver capsule and directly into adjacent adherent organs. Perforation of the visceral peritoneum constitutes pT3 disease, and any roughened area of capsule over the tumour should be sampled for histology.

For right intrahepatic CC, the regional lymph nodes include the hilar (common bile duct, hepatic artery, portal vein and cystic duct), periduodenal and peripancreatic lymph nodes. For left intrahepatic CC, regional lymph nodes include hilar and gastrohepatic lymph nodes. For either type of intrahepatic CC, spread to the coeliac and/or periaortic and caval lymph nodes represent distant metastases (M1).

7. Written report

Needle biopsy - The report should include the following:

- the clinical information received with the biopsy
- a macroscopic description including biopsy size
- the presence or absence of tissue from the focal lesion, and of liver tissue (hepatocytes, bile ducts) as histological confirmation that the specimen is indeed from the liver
- a morphological description of the lesion
- the results of any additional stains carried out, including immunohistochemistry a comment on the background liver, if sufficient is included
- a definite diagnosis of the focal lesion where possible, or a discussion of the differential diagnosis. This would include a discussion of tumours compatible with or excluded by immunohistochemistry
- an appropriate SNOMED code

Supplementary Text 3 Delphi participants

Pathologist survey

Prof Jun Akiba (Kurume University Hospital, Kurume, Japan), Dr Mohammed Al-Masqari (The Royal Hospital, Muscat, Oman), Dr Halima Aliyu (Ahmadu Bello University and Ahmadu Bello University Teaching Hospital, Zaria, Nigeria), Prof Venancio Alves (University of Sao Paulo School of Medicine, Sao Paulo, Brazil), Dr Marife Bonifacio (St Luke's Medical Center, Quezon City and Global City, Metro Manila, Philippines), Prof Paula Borralho Nunes (Faculdade de Medicina da Universidade de Lisboa, Lisbon, Portugal), Prof Alastair Burt (Newcastle University, Newcastle upon Tyne, United Kingdom), Dr Anthony Chan (The Chinese University of Hong Kong, Hong Kong, China), Dr LM. Chinchilla-Tábora (University Hospital of Salamanca, Salamanca, Spain), Dr Caroline Cooper (Pathology Queensland, Brisbane, Australia), Dr Alyn Cratchley (Leeds Teaching Hospitals NHS Trust, Leeds, UK), Dr James M Crawford (Donald and Barbara Zucker School of Medicine at Hofstra/Northwell, NY, USA), Dr Sofía del Carmen (Hospital Universitario Marqués de Valdecilla, Santander, Spain), Dr Rouchelle dela Cruz (Makati Medical Center, Makati City, Philippines), Mrs Alba Diaz (Hospital Clínic, Barcelona, Spain), Prof David Driman (London Health Sciences Centre and Schulich School of Medicine and Dentistry, Western University, London, Canada), Dr Fraser Duthie (NHS Greater Glasgow and Clyde, Glasgow,

United Kingdom), Dr Isioma U. Egbuniwe (University of Nottingham/ Nottingham University Hospitals NHS Trust, Nottingham, United Kingdom), Prof Dr Matthias Evert (Institute for Pathology, University of Regensburg, Regensburg, Germany), Dr Kenneth Fleming (Emeritus Fellow, Green Templeton College, University of Oxford, UK), Dr Luiz Freitas (School of Medicine of The Federal University of Bahia, Salvador-Bahia, Brazil), Dr Alessandro Gambella (Department of Medical Sciences - University of Turin, Turin, Italy), Dr Maddi Garmendia (Osakidetza, Donostia - SAN Sebastian, Spain), Prof Eugenio Gaudio (Sapienza University, Rome, Italy), Dr Ryan Gill (University of California, San Francisco, USA), Prof Robert Goldin (Imperial College, London, UK), Dr M. Augusta Gomes Cipriano (Centro Hospitalar Universitário de Coimbra, Coimbra, Portugal), Dr Zachary Goodman (Inova Fairfax Hospital, Falls Church, Virginia, USA), Dr Judit Halász (Semmelweis University Department of Pathology, Forensic and Insurance Medicine, Budapest, Hungary), Prof Seung-Mo Hong (Asan Medical Center, University of Ulsan College of Medicine, Seoul, Republic of Korea), Prof Prodromos Hytiroglou (Aristotle University, Thessaloniki, Greece), Prof Yung-Ming Jeng (National Taiwan University Hospital, Taipei, Taiwan), Dr Haeryoung Kim (Seoul National University Hospital, Seoul, Republic of Korea), Prof Dr Mina Komuta (International University of Health and Welfare, School of Medicine, Narita Hospital, Narita, JAPAN), Associate Prof. Supinda Koonmee (Faculty of Medicine, Khon Kaen university, Khon Kaen, Thailand), Dr Maalini Krishnasamy (Hospital Selayang, Selayang, Malaysia), Dr Hanna Lapsar (Medical laboratory CSD, Kyiv, Ukraine), Dr Wei-Qiang Leow (Department of Anatomical Pathology, Singapore General Hospital, Singapore, Singapore), Dr Regina Lo (The University of Hong Kong, Hong Kong, Hong Kong, China), Dr Paulo Giovanni Mendoza (Cardinal Santos Mendical Center, San Juan Metro Manila, Philippines), Dr Rui Oliveira (Germano de Sousa - Centro de Diagnóstico Histopatológico CEDAP, Coimbra, Portugal), Prof Young Nyun Park (Yonsei university, Seoul, Korea), Dr Federica Pedica (IRCCS San Raffaele Scientific Institute, Milan, Italia), Dr Glenda Lyn Pua (St. Luke's Medical Center, Quezon City, Philippines), Dr Pavitratha Puspanathan (Hospital Pulau Pinang, Pulau Pinang, Malaysia), Prof Alberto Quaglia (Department of Cellular Pathology, Royal Free London and UCL Cancer Institute, London, United Kingdom), Dr Alphania Rahniayu (Dept. Anatomical Pathology, Faculty of Medicine, Universitas Airlangga, Surabaya, Indonesia), Prof Dr Tania Roskams (University of Leuven, Leuven, Belgium), Dr Puja Sakhuja (GB Pant Institute of Postgraduate Medical Education and Research, New Delhi, India), Prof Dr Christine Sempoux (Service of Clinical Pathology, Institute of Pathology, Lausanne University Hospital, University of Lausanne, Lausanne, Switzerland), Dr Aatur Singhi (University of Pittsburgh Medical Center, Pittsburgh, USA), Dr Marini Stephanie (Cipto Mangunkusumo Hospital, Jakarta, Indonesia), Dr Ashley Stueck (Dalhousie University, Halifax, NS, Canada), Prof Dina Tiniakos (Aretaieion Hospital, Medical School, National and Kapodistrian University of Athens, Athens, Greece), Dr Mihhail Vassiljev (North Estonian Medical Center Foundation, Tallinn, Estonia), Prof Dr Joanne Verheij (Amsterdam UMC, Cancer Center Amsterdam, location University of Amsterdam, Department of Pathology, Amsterdam, The Netherlands), Dr Mukul Vij (Dr Rela institute and medical center, Chennai, India), Associate Professor Lai Mun Wang (Changi General Hospital, Singapore, Singapore), Dr Judy Wyatt (Leeds Teaching Hospitals NHS Trust, Leeds, UK)

‘End-user’ survey

Dr Jorge Adeva (Hospital Universitario 12 de octubre, Madrid, Spain), Drr Mohamed Bouattour (Beaujon University Hospital, Clichy, France), Dr Chiara Braconi (University of Glasgow, Glasgow, United KIngdom), Dr Vincenzo Cardinale (Sapienza University of Rome, Rome, Italy), Prof Massimo Colombo (IRCCS San Raffaele Hospital, Milan, Italy), Dr Cristina Dopazo (Hospital Universitario Vall d’Hebron, Barcelona, Spain), Prof Julien Edeline (Centre Eugène Marquis, Rennes, France), Dr Alejandro Forner (University Barcelona, Barcelona, Spain), Dr Bas Groot Koerkamp (Erasmus MC, Rotterdam, Netherlands), Prof Juozas Kupcinskas (Lithuanian University of Health Sciences, Kaunas,

Lithuania), Dr Angela Lamarca (Fundacion Jimenez Diaz University Hospital, Madrid, Spain), Dr Vera Megdanova (University hospital Queen Yoana -ISUL, Sofia, Bulgaria), Dr Anu Planken (North-Estonian Medical Centre, Tallinn, Estonia), Dr Mariano Ponz Sarvise (Clínica Universidad de Navarra, Pamplona, Spain), Dr Francesca Ratti (Hepatobiliary Surgery Division San Raffaele Hospital, Milano, Italy), Professor Bruno Sangro (Clinica Universidad de Navarra, Pamplona, Spain), Prof Juan Valle (University of Manchester / The Christie, Manchester, United Kingdom), Dr Gianpaolo Vidili (University of Sassari, Sassari, Italy), Prof Dr Henning Wege (Medical Center Esslingen, Esslingen, Germany)

Supplementary Figures

Fig. S1

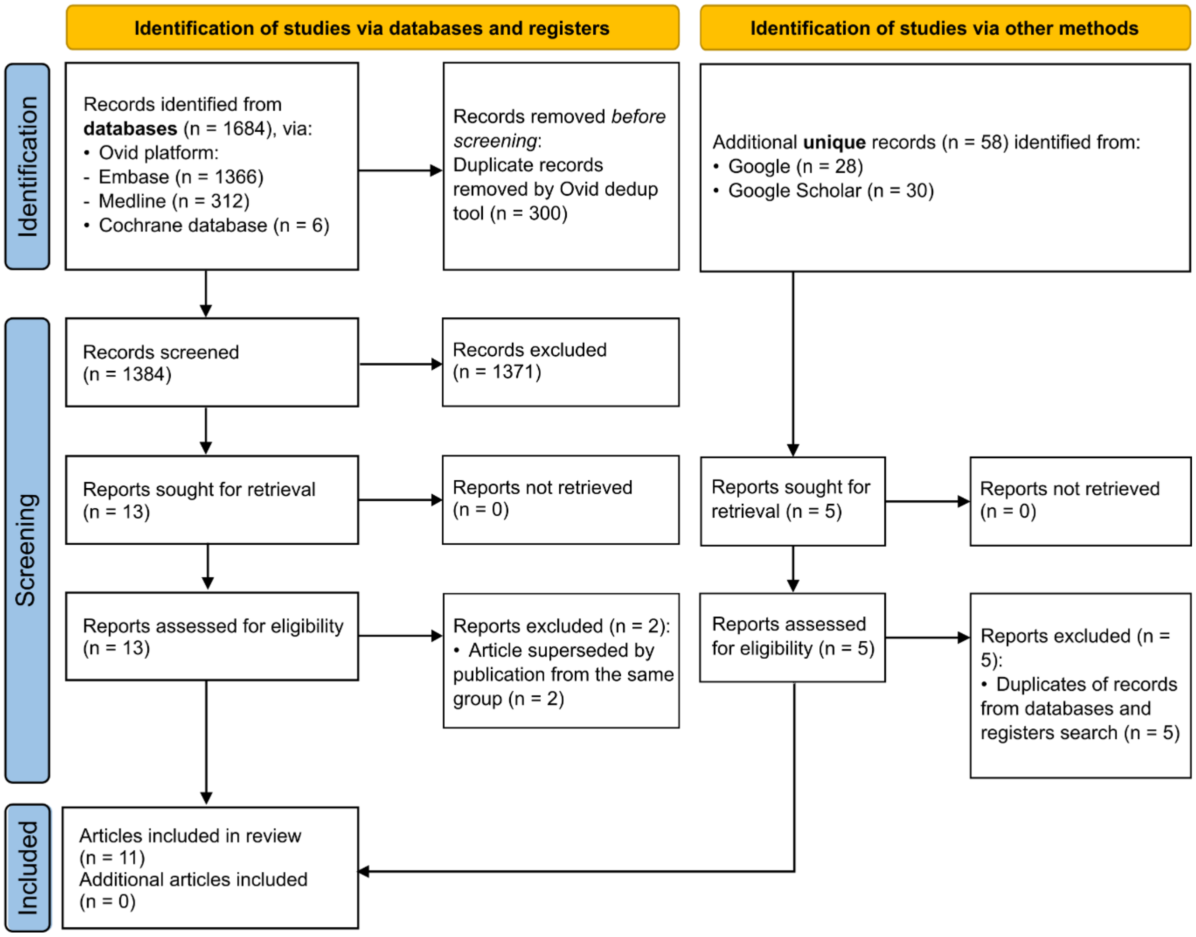

Fig. S1 PRISMA flow diagram of the selection process of guidance resources

Fig. S2

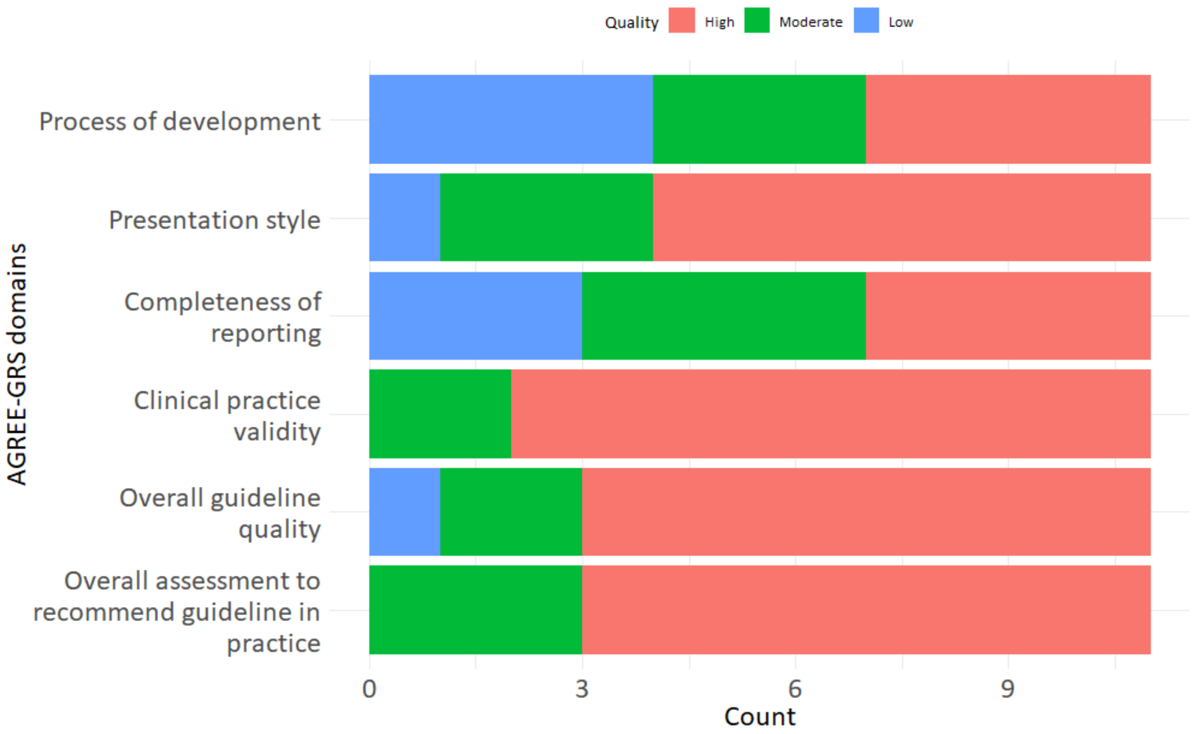

Fig. S2 Number of guidance resources showing low, moderate, and high quality across AGREE-GRS domains

Fig. S3

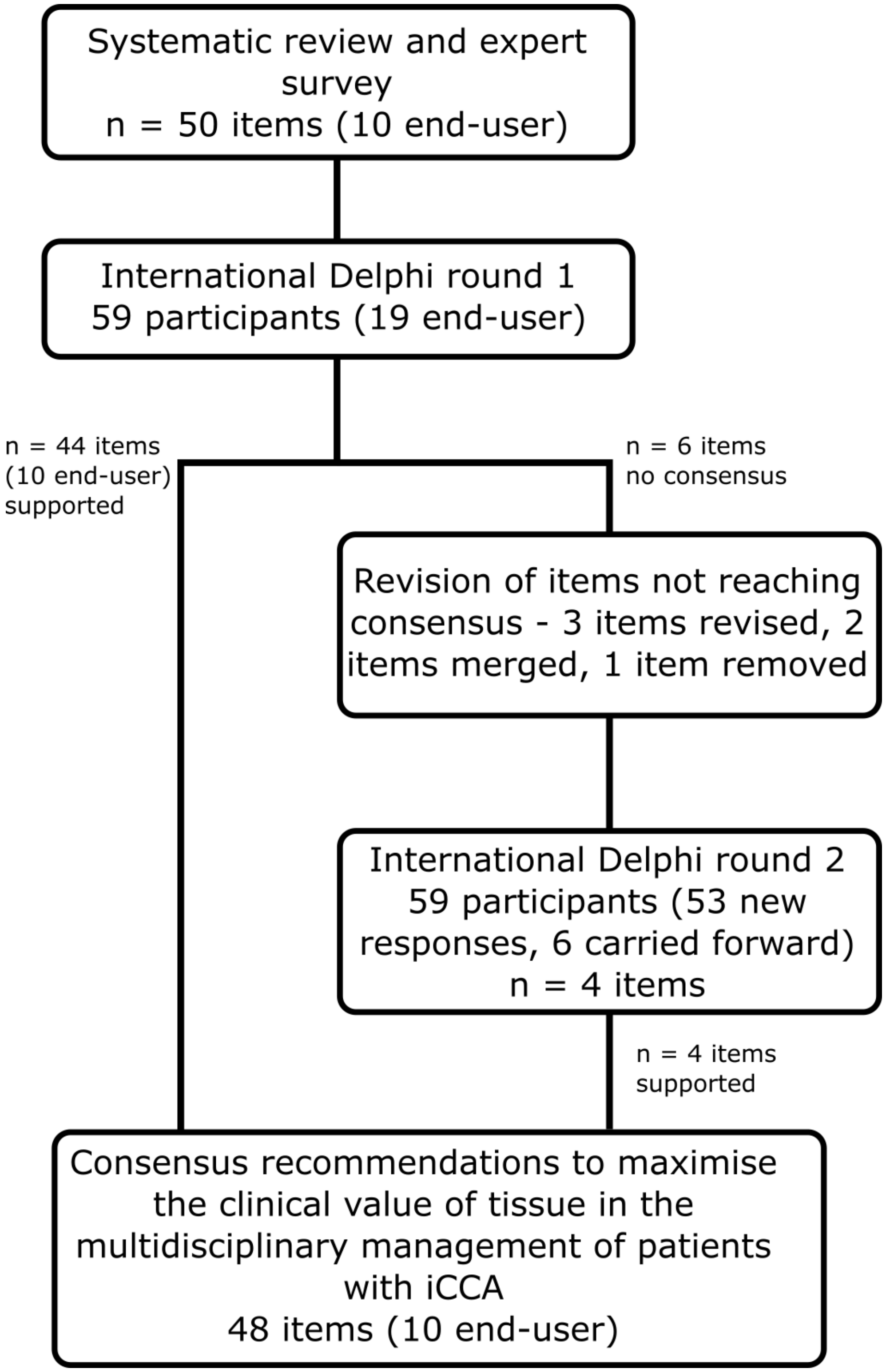

Fig. S3 iCCA recommendation development diagram

Supplementary Tables

Table S1 Systematic review sources and guidance mapping

Category	Number	Percentage	References
1. Specimen fixation	1	9.09	Cong[12]
2. Macroscopy/cut up/trimming of the gross specimen	7	63.64	Burt[13], Cong[12], Jiang[14], Khan[15], Miyazaki[16], Washington[17], Wyatt[18]
3. Block processing and routine tinctorial staining	2	18.18	Cong[12], Wyatt[18]
4. Microscopic assessment	10	90.91	Bridgewater[19], Burt[13], Cong[12], Geller[20], Jiang[14], Khan[15], Krampitz[21], Miyazaki[16], Washington[17], Wyatt[18]
5. Immunohistochemistry	7	63.64	Bridgewater[19], Burt[13], Cong[12], Fernandez Morro[22], Geller[20], Jiang[14], Khan[15]
6. Written report	7	63.64	Bridgewater[19], Burt[13], Jiang[14], Khan[15], Krampitz[21], Washington[17], Wyatt[18]

Table S2 Delphi participant aggregated demographics and experience

Participants	Pathologist, number (total = 59)	User, number (total = 19)
Years experience reporting/managing iCCA		
1 to 5 years	10	1
6 to 10 years	14	7
More than 10 years	35	11
Number of iCCA cases/year		
10 or fewer	12	5
11 to 24	22	6
25 to 49	16	5
50 to 100	7	1
Greater than 100	2	2
Location		
Africa	1	0
Asia	22	0
Europe	27	19
North America	6	0
Oceania	1	0
South America	2	0

Table S3 Delphi responses

Round 1				Round 2			
Statement	% scored 1-3	% scored 7-9	Item status	Statement	% scored 1-3	% scored 7-9	Item status
Specimen handling and fixation							
a. The bile duct margin in a resection should be assessed by intraoperative frozen section	12.07	53.45	none	a. Although more relevant in resections of perihilar rather than intrahepatic cholangiocarcinomas, assessment of the bile duct margin by intraoperative frozen section may be advantageous if the surgeon considers it to be close to the tumour.	0	91.07	in
b. Whole tumour resection specimens should be sent fresh (not in fixative) to the pathology department	15.79	61.4	none	b. If tumour sampling is routinely undertaken for research, biobanking, or molecular testing and the specimen will be received and handled by the pathology department without delay, the whole tumour resection specimen should be sent fresh (not in fixative) to the pathology department.	1.69	93.22	in
c. Where possible, tumour should be sampled fresh and cryopreserved for molecular analysis	7.02	77.19	in				
d. Tissues should be fixed in formalin for at least 24-hours before macroscopic examination and block taking (for resections) or routine processing (for biopsies)	13.56	77.97	in				
Macroscopy/cut up/trimming of the gross specimen							
a. Dissection - Resection surfaces other than the liver capsule should be painted/inked.	1.72	96.55	in				
b. Dissection - The specimen should be sliced into slices no more than 10 mm thick in the horizontal plane.	3.45	86.21	in				
c. Macroscopic description - The macroscopic growth pattern (mass-forming, periductal infiltrating, intraductal, mixed) should be described.	0	86.44	in				
d. Macroscopic description - The size of tumours should be recorded.	0	100	in				
e. Macroscopic description - The location of tumours should be recorded.	0	94.92	in				
f. Macroscopic description - The macroscopic status of any resection margin(s) and nearest distance to tumour should be recorded.	0	100	in				
g. Macroscopic description - The presence or absence of macroscopic vascular invasion should be recorded.	1.72	93.1	in				
h. Macroscopic description - The presence or absence of macroscopic invasion of named bile ducts should be recorded.	1.72	91.38	in				
i. Macroscopic description - The integrity of the liver capsule should be recorded.	0	96.61	in				
j. Macroscopic description - The macroscopic appearance of the non-lesional liver should be recorded.	0	98.31	in				
k. Block sampling - If the tumour is close enough, a single intact tissue block containing tumour and the nearest resection margin should be taken.	3.39	93.22	in				
l. Block sampling - Tumour with adjacent non-lesional liver should be sampled.	0	100	in				
m. Block sampling - Peripheral liver with the closest overlying liver capsule should be sampled if the tumour is subjacent, overlying tissue is adherent, or there is macroscopic invasion of the liver capsule.	0	96.61	in				
n. Block sampling - The gallbladder bed and wall should be sampled where the tumour is adjacent.	0	98.31	in				
o. Block sampling - Areas suspicious for macrovascular invasion should be sampled.	0	100	in				
p. Block sampling - Areas suspicious for bile duct invasion should be sampled.	0	96.61	in				
q. Block sampling - Any bile duct resection margin(s) should be sampled.	0	98.31	in				
r. Block sampling - Non-lesional liver distant from the tumour should be sampled.	0	96.61	in				
s. Block sampling - An intratumoural block suitable for molecular testing should be taken or identified.	1.69	88.14	in				
Block processing and routine tinctorial staining							
a. 5-micron or thinner H&E-stained sections should be prepared from each block.	0	96.55	in				
Microscopic assessment							
a. For a diagnosis of intrahepatic cholangiocarcinoma to be made, the tumour should be an adenocarcinoma.	11.86	61.02	none	a/b. Intrahepatic cholangiocarcinoma is usually adenocarcinoma with identifiable glands. However, poorly-differentiated solid cholangiocarcinomas and rarer morphological subtypes (e.g. adenosquamous, squamous, mucinous, signet-ring, clear cell, mucoepidermoid, lymphoepithelioma-like, and sarcomatous) are described.	3.77	90.57	in
b. Tumours with a variety of histological patterns can be diagnosed as intrahepatic cholangiocarcinoma i.e. no specific microscopic pattern is required.	17.24	65.52	none	Merged with above			
c. For a histological diagnosis of intrahepatic cholangiocarcinoma to be made, desmoplastic stroma must be present.	32.2	33.9	none	c. Desmoplastic stroma is often a prominent feature of intrahepatic cholangiocarcinoma. However, its absence does not preclude a diagnosis of cholangiocarcinoma as its distribution within a tumour may mean it is not present in needle biopsy material.	3.39	84.75	in
d. Biliary intraepithelial neoplasia (BilIN) adjacent to an adenocarcinoma with desmoplastic stroma is highly suggestive of cholangiocarcinoma.	3.39	83.05	in				
e. Large duct-type intrahepatic cholangiocarcinoma is composed of columnar cells.	3.39	83.05	in				
f. The cells of large duct-type intrahepatic cholangiocarcinoma are mucin-producing.	1.69	81.36	in				
g. The cells of large duct-type intrahepatic cholangiocarcinoma are usually arranged in large malignant duct formations or have papillary architecture.	3.39	84.75	in				
h. Small duct-type intrahepatic cholangiocarcinomas usually show no or minimal mucin production.	0	88.14	in				
i. Small duct-type intrahepatic cholangiocarcinomas have a predominant component of cuboidal to polygonal cells....75	0	96.55	in				
j. Small duct-type intrahepatic cholangiocarcinoma cells are predominantly arranged as small to intermediate-sized tubules and/or anastomosing glands.	1.69	96.61	in				
Immunohistochemistry							
a. No immunohistochemical pattern is specific for intrahepatic cholangiocarcinoma.	5.17	79.31	in				
b. Distinction of intrahepatic cholangiocarcinoma from a liver metastasis of a primary upper gastrointestinal, pancreatic, or extrahepatic biliary tumour often cannot be made based on morphology and immunohistochemical profile.	3.39	89.83	in				
c. Intrahepatic cholangiocarcinomas express typical pancreaticobiliary cytokeratins.	0	93.1	in				
d. Immunohistochemistry can always differentiate intrahepatic cholangiocarcinoma from hepatocellular carcinoma.	29.31	37.93	none	Removed			
e. Immunohistochemistry can help differentiate intrahepatic cholangiocarcinoma from hepatocellular carcinoma.	1.69	94.92	in				
Molecular testing							
a. Molecular testing, where available, including for FGFR abnormalities, IDH mutations, and any other abnormality with therapeutic significance should be undertaken.	0	80.7	in				
Written report and interpretation							
a. The AJCC/UICCA staging schema should be used for the reporting of resection specimens, and the stage included in the primary report.	0	96.61	in				
b. The results of any molecular analysis and immunohistochemical staining should be included in the primary report.	6.9	84.48	in				
c. The presence or absence of perineural invasion should be stated in the primary report.	0	94.92	in				
d. The presence or absence of lymphovascular invasion should be stated in the primary report.	0	98.31	in				
e. The presence or absence of dysplasia should be stated in the primary report.	1.69	91.53	in				
f. Small-duct or large-duct designation of intrahepatic cholangiocarcinomas should be stated in the primary report.	1.72	89.66	in				
g. A semiquantitative grading based on the proportion of glands should be stated in the primary report (well differentiated - more than 95% of tumour composed of glands; moderately-differentiated - 50% to 95% of tumour composed of glands; poorly-differentiated - up to 49% of tumour composed of glands).	6.78	77.97	in				
h. The status of any resection margin(s) should be stated in the primary report.	0	100	in				
i. The status of any lymph nodes should be stated in the primary report.	0	100	in				
j. Confident diagnosis requires the correlation of histomorphology and any immunohistochemical staining profile with available imaging and clinical information. Clinical history, endoscopic investigation, and imaging studies are all pivotal in reaching the correct conclusion.	0	94.92	in				

Supplementary References

1 Kendall T, Carpino G, Guido M. EURO-CHOLANGIO-NET diagnostic histopathology systematic review. Published Online First: 22 October 2021.

2 Brouwers MC, Kho ME, Browman GP, *et al.* The Global Rating Scale complements the AGREE II in advancing the quality of practice guidelines. *J Clin Epidemiol*. 2012;65:526–34.

3 Lim SJ, Gurusamy K, O'Connor D, *et al.* Recommendations for cellular and molecular pathology input into clinical trials: a systematic review and meta-aggregation. *The Journal of Pathology: Clinical Research*. 2021;7:191–202.

4 McArthur A, Klugarova J, Yan H, *et al.* Chapter 4: Systematic Reviews of text and opinion. In: Aromataris E, Munn Z, eds. *JBI Manual for Evidence Synthesis*. JBI 2020. https://doi.org/10.46658/JBIMES-20-05

5 McArthur A, Klugarova J, Yan H, *et al.* 4.3.4.5.2 Textual data extraction - Systematic Reviews of text and opinion. JBI Manual for Evidence Synthesis. 2020. https://wiki.joannabriggs.org/display/MANUAL/4.3.4.5.2+Textual+data+extraction (accessed 18 July 2020)

6 Oxford University Press. Oxford English Dictionary. https://www.oed.com/ (accessed 7 July 2020)

7 Carroll C, Booth A, Cooper K. A worked example of "best fit" framework synthesis: A systematic review of views concerning the taking of some potential chemopreventive agents. *BMC Medical Research Methodology*. 2011;11. doi: 10.1186/1471-2288-11-29

8 Carroll C, Booth A, Leaviss J, *et al.* "Best fit" framework synthesis: Refining the method. *BMC Medical Research Methodology*. 2013;13. doi: 10.1186/1471-2288-13-37

9 Brunton G, Oliver S, Thomas J. Innovations in framework synthesis as a systematic review method. *Research Synthesis Methods*. 2020;11:316–30.

10 McArthur A, Klugarova J, Yan H, *et al.* 4.3.4.5.3 Textual data synthesis - Systematic Reviews of text and opinion. JBI Manual for Evidence Synthesis. 2020. https://wiki.joannabriggs.org/display/MANUAL/4.3.4.5.3+Textual+data+synthesis (accessed 20 July 2020)

11 McArthur A, Klugarova J, Yan H, *et al.* 4.3.4.6.3 Findings of the review - Systematic Reviews of text and opinion. JBI Manual for Evidence Synthesis. 2020. https://wiki.joannabriggs.org/display/MANUAL/4.3.4.6.3+Findings+of+the+review (accessed 20 July 2020)

12 Cong W-M, Dong H, Zhu Y-Y, *et al.* Practice guidelines for the pathological diagnosis of primary liver cancer: 2015 update. *World J Gastroenterol*. 2016;22:9279–87.

13 Burt A, Alves V, Coulston A, *et al. Intrahepatic Cholangiocarcinoma, Perihilar Cholangiocarcinoma and Hepatocellular Carcinoma Histopathology Reporting Guide.* 2nd ed. Sydney, Australia: International Collaboration on Cancer Reporting 2020. http://www.iccr-cancer.org/datasets/published-datasets/digestive-tract/liver

14 Jiang K, Al-Diffhala S, Centeno BA. Primary Liver Cancers-Part 1: Histopathology, Differential Diagnoses, and Risk Stratification. *Cancer Control*. 2018;25:1073274817744625.

15 Khan SA, Davidson BR, Goldin RD, *et al.* Guidelines for the diagnosis and treatment of cholangiocarcinoma: an update. *Gut*. 2012;61:1657–69.

16 Miyazaki M, Yoshitomi H, Miyakawa S, *et al.* Clinical practice guidelines for the management of biliary tract cancers 2015: the 2nd English edition. *J Hepatobiliary Pancreat Sci*. 2015;22:249–73.

17 Washington MK, Berlin J, Branton PA, *et al.* Protocol for the Examination of Specimens From Patients With Carcinoma of the Intrahepatic Bile Ducts. *Archives of Pathology & Laboratory Medicine*. 2010;134:e14–8.

18 Wyatt J, Hubscher S, Goldin R. Dataset for histopathology reporting of liver resection specimens (including gall bladder) and liver biopsies for primary and metastatic carcinoma (2nd edition). 2012.

19 Bridgewater J, Galle PR, Khan SA, *et al.* Guidelines for the diagnosis and management of intrahepatic cholangiocarcinoma. *Journal of Hepatology*. 2014;60:1268–89.

20 Geller SA, Dhall D, Alsabeh R. Application of Immunohistochemistry to Liver and Gastrointestinal Neoplasms: Liver, Stomach, Colon, and Pancreas. *Archives of Pathology & Laboratory Medicine*. 2008;132:490–9.

21 Krampitz GW, Aloia TA. Staging of Biliary and Primary Liver Tumors: Current Recommendations and Workup. *Surg Oncol Clin N Am*. 2019;28:663–83.

22 Fernandez Moro C, Fernandez-Woodbridge A, Alistair D'souza M, *et al.* Immunohistochemical Typing of Adenocarcinomas of the Pancreatobiliary System Improves Diagnosis and Prognostic Stratification. *PLoS ONE*. 2016;11:e0166067.
