# Supplementary material for: Recommendations on maximising the clinical value of tissue in the management of patients with intrahepatic cholangiocarcinoma
Source: JHEP Rep. 2024 Mar 12;6(6):101067. doi: 10.1016/j.jhepr.2024.101067 (PMC11060959; doi:10.1016/j.jhepr.2024.101067)
Supplement: Multimedia component 4 [file mmc4.pdf]

Recommendations on maximising the clinical value of tissue in the management of patients with intrahepatic cholangiocarcinoma

Timothy Kendall,[1,*] Diletta Overi,[2] Maria Guido,[3] Chiara Braconi,[4] Jesus Banales,[5,6] Vincenzo Cardinale,[7] Eugenio Gaudio,[2] Bas Groot Koerkamp,[8] Guido Carpino[2]

[1]University of Edinburgh Centre for Inflammation Research and Edinburgh Pathology, University of Edinburgh, Edinburgh, UK; [2]Department of Anatomical, Histological, Forensic Medicine and Orthopedics Sciences, Sapienza University of Rome, Rome, Italy; [3]Department of Medicine, DIMED, University of Padua, Padua, Italy; [4]School of Cancer Sciences, University of Glasgow, CRUK Scotland Cancer Centre, Beatson West of Scotland Cancer Centre, Glasgow, UK; [5]Department of Liver and Gastrointestinal Diseases, Biodonostia Health Research Institute, Donostia University Hospital, CIBERehd and University of the Basque Country (UPV/EHU), San Sebastian, Spain; [6]Department of Biochemistry and Genetics, School of Sciences, University of Navarra, Pamplona, Spain; [7]Department of Translational and Precision Medicine, Sapienza University of Rome, Rome, Italy; [8]Department of Surgery, Erasmus MC Cancer Institute, Rotterdam, the Netherlands

JHEP Reports **2024.** https://doi.org/10.1016/j.jhepr.2024.101067

Background & Aims: Patients with intrahepatic cholangiocarcinoma can now be managed with targeted therapies directed against specific molecular alterations. Consequently, tissue samples submitted to the pathology department must produce molecular information in addition to a diagnosis or, for resection specimens, staging information. The pathologist's role when evaluating these specimens has therefore changed to accommodate such personalised approaches.
Methods: We developed recommendations and guidance for pathologists by conducting a systematic review of existing guidance to generate candidate statements followed by an international Delphi process. Fifty-nine pathologists from 28 countries in six continents rated statements mapped to all elements of the specimen pathway from receipt in the pathology department to authorisation of the final written report. A separate survey of 'end-users' of the report including surgeons, oncologists, and gastroenterologists was undertaken to evaluate what information should be included in the written report to enable appropriate patient management.
Results: Forty-eight statements reached consensus for inclusion in the guidance including 10 statements about the content of the written report that also reached consensus by end-user participants. A reporting proforma to allow easy inclusion of the recommended data points was developed.
Conclusions: These guiding principles and recommendations provide a framework to allow pathologists reporting on patients with intrahepatic cholangiocarcinoma to maximise the informational yield of specimens required for personalised patient management.
Impact and Implications: Biopsy or resection lesional tissue from intrahepatic cholangiocarcinoma must yield information about the molecular abnormalities within the tumour that define suitability for personalised therapies in addition to a diagnosis and staging information. Here, we have developed international consensus guidance for pathologists that report such cases using a Delphi process that sought the views of both pathologists and 'end-users of pathology reports. The guide highlights the need to report cases in a way that preserves tissue for molecular testing and emphasises that reporting requires interpretation of histological characteristics within the broader clinical and radiological context. The guide will allow pathologists to report cases of intrahepatic cholangiocarcinoma in a uniform manner that maximises the value of the tissue received to facilitate optimal multidisciplinary patient management.
© 2024 The Authors. Published by Elsevier B.V. on behalf of European Association for the Study of the Liver (EASL). This is an open access article under the CC BY license (http://creativecommons.org/licenses/by/4.0/).

Introduction

Cholangiocarcinoma (CCA) is the most common malignancy of the biliary tree. It is divided into intrahepatic (iCCA), perihilar (pCCA), and distal (dCCA) types; each a separate entity with distinct clinical, histopathological, and molecular features. iCCAs arise within the liver parenchyma from intrahepatic ducts proximal to second-order bile ducts, and are commonly mass-forming lesions, featuring molecular abnormalities with potential therapeutic targets in up to 50% of cases.

Keywords: Cholangiocarcinoma; Guidelines; Histopathology; Management; Molecular testing; Multidisciplinary.
Received 6 January 2024; received in revised form 4 March 2024; accepted 8 March 2024; available online 12 March 2024
* Corresponding author. Address: University of Edinburgh Centre for Inflammation Research, Institute for Regeneration and Repair, University of Edinburgh, 4-5 Little France Drive, Edinburgh BioQuarter, Edinburgh EH16 4UU, UK. Tel.: +44 (0)131 2427007.
E-mail address: Tim.Kendall@ed.ac.uk (T. Kendall).

ELSEVIER
EASL The Home of Hepatology

Although guidelines for the management of patients with iCCA are available, recommendations for the pathological pathway, with reference to specimen receipt, tissue storage, tissue processing, analyses to be performed and generation of the final report, are lacking. As personalisation based on mutational profile is increasingly common in routine clinical practice, for example through FDA- and EMA-licensed therapies for patients whose tumours bear *FGFR2* fusions or *IDH1* mutations,[1] we sought to define recommendations that serve to maximise the overall clinical value of tissue sampling for the management of patients with iCCA. The guidance emphasises when and how molecular characterisation can be effectively undertaken within a routine reporting pathway. Patient management is the most effective when using a multidisciplinary team approach, so we also sought the input of actual end-users of the final authorised pathological report to make our pathway clinically impactful.

Herein, we describe the development of consensus recommendations achieved through an international Delphi process. The adoption of these recommendations will maximise the clinical value of tissue samples for the management of patients with iCCA. For each recommendation, we provide a rationale and relevant references providing context and supporting its utility.

Materials and methods

Systematic review

We undertook a systematic review of published expert opinions[2] on the subject of histological reporting of iCCA cases to inform the generation of Delphi candidate items for subsequent rating. Full details are available in the Supplementary Methods.

International Delphi exercise

Candidate Delphi items were generated by synthesising the extracted guidance identified by the systematic review. Items were grouped into the stages of the reporting pathway.

The core research group (TK, MG, GC, DO) revised items to make them thematically coherent and to ensure that parts of the reporting pathway were fully addressed.

Respondents were asked to rate the importance of each item on a nine-point Likert-type scale. Pathologist respondents rated all items, and "end-users" (*i.e.*, surgeons, oncologists, gastroenterologists) of histopathology reports participated in a sub-survey rating only items relating to the final written pathology report. Two rounds were undertaken to reach consensus. Full details of the Delphi process are provided in the Supplementary Methods.

Results

Systematic review

Selection of guidance resources

The literature searches identified 1,742 records, 312 from Medline (Ovid), 1,366 from Embase (Ovid), and six from the Cochrane database. Web searches identified another 56 unique records. No grey literature was found. The titles and abstracts of 1,384 database records were screened after de-duplication. Of these, 1,371 records from the database searches were excluded by titles and abstracts: 53 of 58 records from Google/Google Scholar searches were excluded by title, and five records were duplicates of reports assessed for eligibility by full-text review from database searches. Of the 13 full-text articles retrieved and assessed for eligibility, 11 were included in the review and two were excluded as records that had been superseded by other records within those included issued by the same organisation (Fig. S1).[3]

Characteristics and mapping of guidance resources

Extracted statements from the individual resources were mapped onto the stages of the specimen pathway, and included specimen handling and fixation, macroscopy/slicing/trimming of the gross specimen, block processing, routine tinctorial staining, microscopic assessment, immunohistochemistry, molecular testing, and written report and interpretation (Table S1). Of the 11 guidance resources included,[4–14] ten offered recommendations about the microscopic assessment[4–6,8–14] and seven contained recommendations about macroscopic assessment,[5,6,8,9,11–13] immunohistochemical evaluation,[4–9,14] and the content of the final authorised report.[4,5,8–10,12,13] Eight resources contained explicit recommendations,[4–7,9,11–13] two contained implicit recommendations,[10,14] and one had a combination of implicit and explicit recommendations.[8]

The verbatim extracted guidance from each source, mapped to the specimen pathway categories, is provided in Supplementary Text 2. The extracted guidance from each source was synthesised thematically and was used to generate the initial candidate Delphi statements.

Quality assessment of individual guidance resources

The majority of the guidance resources were high quality (Appraisal of Guidelines for Research & Evaluation Global Rating Scale [AGREE-GRS] score of >66%). However, the quality was lower (AGREE-GRS score of <34%) in the AGREE-GRS domains of 'process of development' and 'completeness of reporting'. All guidance resources were considered highly relevant and applicable to reporting practice (Fig. S2).

The Delphi process

Characteristics of the Delphi participants (Supplementary Text 3) are summarised in Table S2. Full details of the Delphi responses are shown in Table S3, and the process is outlined in Fig. S3.

Pathologists

For the full survey of pathologists, invitations to participate in Round One of the Delphi exercise were sent to 97 individuals. Of these, 59 pathologists from 28 countries across Asia, Africa, Europe, North America, Oceania, and South America consented to participate and completed Round One.

Fifty statements were assessed: 44 reached consensus-in status and the remaining six did not reach consensus (items #1, #2, #25, #26, #27, #40). All statements regarding information that should be included in the written report were classified as consensus-in.

There was no variation in opinion or consensus based on the location of participants.

Statements not reaching consensus were revised or combined based upon participant comments. Three statements (#1, #2, #27) were revised. Two statements (#25, #26) were combined into a single revised statement. One statement (#40) was removed as it was originally included as one of a mutually exclusive pair with statement #39.

Invitations for Round Two were sent to those participants who responded in Round One. Fifty-three participants amended their scores from Round One; those who did not had their Round One scores carried forward to Round Two. All four of the remaining modified items reached consensus-in status.

End-users
Sixty-five end-users were invited to participate in Round One of the end-user survey. Nineteen participants completed Round One of the survey rating statements about what should be included in the pathology written report. All ten statements reached consensus-in status.

Consensus recommendations maximising the clinical value of tissue and histopathological reporting for the multidisciplinary management of patients with intrahepatic cholangiocarcinoma
The consensus statements providing guidance for the histopathological reporting of iCCA were grouped by their position in the reporting pathway between receipt of the specimen in the pathology department and the authorisation of the final written pathology report as summarised in Table 1. The agreed statements and an elaboration of their relevance and context are provided, and a reporting proforma capturing all recommended data points has been developed (Supplementary Files 1 and 2).

Specimen handling and fixation

1. *Although more relevant in resections of perihilar rather than intrahepatic CCAs, assessment of the bile duct margin by intraoperative frozen section may be advantageous if the surgeon considers it to be close to the tumour.*

Data regarding the use of intraoperative frozen sections to assess the bile duct margin are restricted to patients with pCCA where the anatomy and tumour growth pattern present specific surgical challenges. Even in this setting, the data are contradictory about whether routine assessment of the bile duct margin offers a survival advantage.[15,16] No specific studies have examined frozen sections for resections of iCCA. The consensus recommendation is that submission of the bile duct margin for histopathological examination by intraoperative frozen section should be at the discretion of the operating surgeon if the tumour is macroscopically close to the margin. Intraoperative frozen sections may also be useful in the diagnosis of potential intrahepatic metastatic lesions whose nature influences the immediate surgical management.[17]

2. *If tumour sampling is routinely undertaken for research, biobanking, or molecular testing, the specimen should be received and handled by the pathology department without delay. The whole tumour resection specimen should be sent fresh (not in fixative) to the pathology department.*
3. *Where possible, the tumour should be sampled fresh and cryopreserved for molecular analysis.*

Molecular assays used in clinical practice typically rely on formalin-fixed paraffin-embedded (FFPE) tissue, and such tested FFPE material is increasingly reliable and has become a core part of clinical workflows as technologies evolve.[18–20] However, fresh or frozen material may allow additional testing strategies to be developed and can facilitate fundamental research, for example with tumour-derived organoids. Any sampling of specimens for research purposes should only be undertaken after all required ethical approvals have been obtained and their conditions, including informed consent, have been satisfied.

If authorised sampling of tumours in resection specimens is required, and it can be undertaken in the pathology laboratory prior to fixation without impacting upon the standard clinical specimen pathway and compromising resection margins (*e.g.* as undertaken in the HepaT1ca study[21]), the specimen should be sent fresh and the tumour sampled as soon as possible as significant biochemical alterations can be identified even after only 10 min of tissue anoxia and histological alterations, such as a loss of identifiable mitotic figures, can be observed with a fixation delay of only 2 h.[22]

4. *Tissues should be fixed in formalin for at least 24 h before macroscopic examination and block sampling (for resections) or routine processing (for biopsies).*

We recommend that specimens be fixed in formalin following the standard local procedures and for at least 24 h. Fixation preserves tumour morphology and optimises the performance of clinical immunohistochemistry. Appropriate fixation of resection specimens allows these to be optimally cut and evaluated macroscopically.

Macroscopy, slicing, and trimming of the gross specimen
Macroscopic assessment of, and block sampling from gross resection specimens should be undertaken in a coordinated manner that allows the required information from the specimen to be obtained. Some recommendations below relate to information with proven clinical utility. Others relate to pathological macroscopic features that have a strong biological rationale but require further study that will be enabled but routine data collection.

Dissection.

5. *Resection surfaces other than the liver capsule should be painted/inked.*

Tissue-marking dyes are commonly applied to resection specimens prior to any dissection or sectioning to mark both surgical margins and other landmarks, and the use of different colours allows the orientation of specimens to be preserved; we recommend the use of such dyes. In resections for intrahepatic lesions, including iCCA, only a single margin is usually found. The presence of ink on the subsequent histological section allows confident identification of the surgical margin so that the completeness of resection and surgical clearance can be determined. The inked resection margins should ideally be 'fixed' and dried before sectioning to reduce the likelihood that surface ink is introduced into the specimen upon slicing.[23] The surgeon may indicate the vascular margin in cases where this is relevant.

6. *The specimen should be sliced into slices no more than 10 mm thick in the horizontal plane.*

We recommend that initial slicing of a resection specimen should only be undertaken once it is well fixed to ensure that slices no thicker than 10 mm can be made. These initial slices should be made perpendicular to the resection margin(s), often in the axial plane. Sectioning at 10 mm or less allows small lesions to be identified and allows the surgical clearance margin to be evaluated.

Macroscopic description. After initial sectioning of the resection specimen, macroscopic description of many of the core dataset

Table 1. Consensus recommendation statements for maximising the clinical value of tissue in the reporting of intrahepatic cholangiocarcinoma.

Item number	Recommendation
**Specimen handling and fixation**	
1	Although more relevant in resections of perihilar rather than intrahepatic cholangiocarcinomas, assessment of the bile duct margin by intraoperative frozen section may be advantageous if the surgeon considers it to be close to the tumour.
2	If tumour sampling is routinely undertaken for research, biobanking, or molecular testing and the specimen will be received and handled by the pathology department without delay, the whole tumour resection specimen should be sent fresh (not in fixative) to the pathology department.
3	Where possible, tumour should be sampled fresh and cryopreserved for molecular analysis.
4	Tissues should be fixed in formalin for at least 24 h before macroscopic examination and block taking (for resections) or routine processing (for biopsies).
**Macroscopy/slicing/trimming of the gross specimen**	
Dissection	
5	Resection surfaces other than the liver capsule should be painted/inked.
6	The specimen should be sliced into slices no more than 10 mm thick in the horizontal plane.
Macroscopic description	
7	The macroscopic growth pattern (mass-forming, periductal infiltrating, intraductal, mixed) should be described.
8	The size of tumours should be recorded.
9	The location of tumours should be recorded.
10	The macroscopic status of any resection margin(s) and nearest distance to tumour should be recorded.
11	The presence or absence of macroscopic vascular invasion should be recorded.
12	The presence or absence of macroscopic invasion of named bile ducts should be recorded.
13	The integrity of the liver capsule should be recorded.
14	The macroscopic appearance of the non-lesional liver should be recorded.
Block sampling	
15	If the tumour is close enough, a single intact tissue block containing tumour and the nearest resection margin should be taken.
16	Tumour with adjacent non-lesional liver should be sampled.
17	Peripheral liver with the closest overlying liver capsule should be sampled if the tumour is subjacent, overlying tissue is adherent, or there is macroscopic invasion of the liver capsule.
18	The gallbladder bed and wall should be sampled where the tumour is adjacent.
19	Areas suspicious for macrovascular invasion should be sampled.
20	Areas suspicious for bile duct invasion should be sampled.
21	Any bile duct resection margin(s) should be sampled.
22	Non-lesional liver distant from the tumour should be sampled.
23	An intratumoural block suitable for molecular testing should be taken or identified.
**Block processing and routine tinctorial staining**	
24	Five micron or thinner H&E-stained sections should be prepared from each block.
**Microscopic assessment**	
25	Intrahepatic cholangiocarcinoma is usually adenocarcinoma with identifiable glands. However, poorly-differentiated solid cholangiocarcinomas and rarer morphological subtypes (*e.g.* adenosquamous, squamous, mucinous, signet-ring, clear cell, mucoepidermoid, lymphoepithelioma-like, and sarcomatous) are described.
26	Desmoplastic stroma is often a prominent feature of intrahepatic cholangiocarcinoma. However, its absence does not preclude a diagnosis of cholangiocarcinoma as its distribution within a tumour may mean it is not present in needle biopsy material.
27	Biliary intraepithelial neoplasia (BilIN) adjacent to an adenocarcinoma with desmoplastic stroma is highly suggestive of cholangiocarcinoma.
28	Large duct-type intrahepatic cholangiocarcinoma is composed of columnar cells.
29	The cells of large duct-type intrahepatic cholangiocarcinoma are mucin-producing.
30	The cells of large duct-type intrahepatic cholangiocarcinoma are usually arranged in large malignant duct formations or have papillary architecture.
31	Small duct-type intrahepatic cholangiocarcinomas usually show no or minimal mucin production.
32	Small duct-type intrahepatic cholangiocarcinomas have a predominant component of cuboidal to polygonal cells.
33	Small duct-type intrahepatic cholangiocarcinoma cells are predominantly arranged as small to intermediate-sized tubules and/or anastomosing glands.
**Immunohistochemistry**	
34	No immunohistochemical pattern is specific for intrahepatic cholangiocarcinoma.
35	Distinction of intrahepatic cholangiocarcinoma from a liver metastasis of a primary upper gastrointestinal, pancreatic, or extrahepatic biliary tumour often cannot be made based on morphology and immunohistochemical profile.
36	Intrahepatic cholangiocarcinomas express typical pancreaticobiliary cytokeratins.
37	Immunohistochemistry can help differentiate intrahepatic cholangiocarcinoma from hepatocellular carcinoma.
**Molecular testing**	
38	Molecular testing, where available, including for *FGFR* abnormalities, *IDH* mutations, and any other abnormality with therapeutic significance should be undertaken.
**Written report and interpretation**	
39	The AJCC/UICCA staging schema should be used for the reporting of resection specimens, and the stage included in the primary report.
40	The results of any molecular analysis and immunohistochemical staining should be included in the primary report.
41	The presence or absence of perineural invasion should be stated in the primary report.
42	The presence or absence of lymphovascular invasion should be stated in the primary report.
43	The presence or absence of dysplasia should be stated in the primary report.
44	Small-duct or large-duct designation of intrahepatic cholangiocarcinomas should be stated in the primary report.
45	A semiquantitative grading based on the proportion of glands should be stated in the primary report (well-differentiated, >95% of tumour composed of glands; moderately-differentiated, 50%–95% of tumour composed of glands; poorly-differentiated, <49% of tumour composed of glands).

(*continued on next page*)

Table 1 (*continued*)

Item number	Recommendation
46	The status of any resection margin(s) should be stated in the primary report.
47	The status of any lymph nodes should be stated in the primary report.
48	Confident diagnosis requires the correlation of histomorphology and any immunohistochemical staining profile with available imaging and clinical information. Clinical history, endoscopic investigation, and imaging studies are all pivotal in reaching the correct conclusion.

AJCC, American Joint Committee on Cancer; UICCA, International Union Against Cancer.

items that are known to have prognostic or therapeutic importance can be undertaken.

7. *The macroscopic growth pattern (mass-forming, periductal infiltrating, intraductal, mixed) should be described.*

The macroscopic growth pattern of CCA can be mass-forming, periductal infiltrating, intraductal, or mixed although mass-forming is the most common pattern for iCCA. Patients with iCCAs that have a pure mass-forming macroscopic growth pattern probably have a better prognosis than patients with periductal infiltrating tumours although pure intraductal tumours may have a better prognosis.[24–26] We recommend that the macroscopic growth pattern be assessed at the time of specimen dissection.

8. *The size of tumours should be recorded.*

The size of each tumour should be recorded individually, which also captures the total number of tumours. This provides tumour burden, a function of tumour number and tumour size, which is of prognostic significance; patients with tumours larger and more than a single tumour having a worse prognosis.[27–30] In a study of explant livers, patients with single tumours ≤2 cm had very low risk of recurrence and 73% 5-year survival.[31]

Further, a recent study has demonstrated that in the absence of lymph node or extrahepatic spread, patients with iCCA and liver metastases have a worse outcome than patients without liver metastases.[32] Recording the size and number of tumours will allow re-staging of patients should staging systems be modified in light of this data.

9. *The location of tumours should be recorded.*

We recommend that recording of the location of tumours within specimens should be attempted although it is not always easy to do so with complete accuracy. An estimation of which Couinaud segment(s) a tumour is in, and/or its relative location (anterior/posterior, peripheral/central) may allow attempts at correlation with cross-sectional imaging. Classification of the tumour as intrahepatic should be confirmed during macroscopic assessment. If a tumour arises from the first and second order bile ducts it should be classified as a perihilar lesion and is beyond the scope of these guidelines.

10. *The macroscopic status of any resection margin(s) and nearest distance to tumour should be recorded.*

Evaluation of the margin and measurement of the shortest distance from tumour to surgical margin provides significant prognostic information and should be undertaken. R0 resection status and increasing clearance distance are both associated with better prognosis,[30,33–36] and the data is also used in routine audits of surgical practice.

11. *The presence or absence of macroscopic vascular invasion should be recorded.*

We recommend that the presence or absence of vascular invasion should be recorded. Macroscopically identified vascular invasion of large portal or hepatic veins is a very poor prognostic factor in iCCA.[37,38] Often this can be identified pre-operatively and may be a contraindication to surgery.

12. *The presence or absence of macroscopic invasion of named bile ducts should be recorded.*

Invasion of extrahepatic structures including named bile ducts is considered pT4 in the Union for International Cancer Control TNM 8th edition and is associated with poor overall prognosis,[39] and this information should be documented. Bile duct invasion has separately demonstrated to be an adverse prognostic factor when assessed on preoperative imaging.[40]

13. *The integrity of the liver capsule should be recorded.*

Glisson's capsule is the thin layer of interstitial connective tissue surrounding most of the external surface of the liver and extends around hilar vessels. Invasion into or through the capsule should be recorded as it may represent a route to metastasis although specific survival data examining this are lacking.[41]

14. *The macroscopic appearance of the non-lesional liver should be recorded.*

Cirrhosis of any cause is a risk factor for the development of iCCA,[30,42] so the appearance of the non-lesional liver should be commented upon. Although the data about the influence of cirrhosis on outcomes after surgery for iCCA is conflicting,[43,44] the subsequent investigation and management of patients newly diagnosed as having cirrhosis will be different from a patient with no background liver abnormality.

Block sampling.

15. *If the tumour is close enough, a single intact tissue block containing tumour and the nearest resection margin should be taken.*

We recommend that a block showing the relationship of the tumour to the closest margin be taken. This allows microscopic confirmation of the resection margin status and measurement of the clearance distance, a metric predictive of disease-free and overall survival.[35]

16. *Tumour with adjacent non-lesional liver should be sampled.*

The invasive front at the interface between the tumour and the non-lesional liver often remains viable when the centre of large lesions is necrotic and, therefore, unsuitable for the confirmation of diagnosis. Further, there may be research value in separate morphological assessment of the invasive tumour front with potential prognostic significance; for example, increased tumour budding at the invasive front has been shown to be associated with worse recurrence-free and overall survival.[45] We recommend that specific sampling of the interface of the tumour with the non-lesional liver be undertaken.

17. *Peripheral liver with the closest overlying liver capsule should be sampled if the tumour is subjacent, overlying tissue is adherent, or there is macroscopic invasion of the liver capsule.*
18. *The gallbladder bed and wall should be sampled where the tumour is adjacent.*

We recommend sampling to allow microscopic assessment of areas that are macroscopically suspicious of capsular invasion. This permits formal demonstration of tumour invasion through the capsule into adherent extrahepatic structures. Perforation of the visceral peritoneum is classified as stage pT3 and invasion of extrahepatic structures is classified as stage pT4 by the TNM schema.[39] In one study, visceral invasion was histologically confirmed in 45.7% of cases where there was surgical suspicion of invasion leading to visceral resection, and patients with histologically confirmed visceral invasion had a poorer prognosis, not associated with the operative procedure alone.[46]

19. *Areas suspicious for macrovascular invasion should be sampled.*

We recommend that areas macroscopically suspicious of vascular invasion be sampled. Vascular invasion is a feature of the TNM8 T-stage classification, defining pT2 classification in solitary tumours, and should be confirmed microscopically.

20. *Areas suspicious for bile duct invasion should be sampled.*

As noted, bile duct invasion is associated with a poorer prognosis and influences TNM classification. Areas suspicious for bile duct invasion should be sampled to allow this to be confirmed histologically.

21. *Any bile duct resection margin(s) should be sampled.*

Bile duct resection margins, where present, should be sampled to permit histological confirmation of either complete resection or involvement.

22. *Non-lesional liver distant from the tumour should be sampled.*

In resection specimens, we recommend that a block of non-lesional liver distant from the tumour be sampled, irrespective of the macroscopic impression. Histological assessment of non-lesional liver distant from the tumour allows diagnosis or confirmation of parenchymal disease that may influence the postoperative management of the patient. Further, this allows an interpretation of the tumour alongside possible underlying and contributory parenchymal or biliary disease.

Where a patient is undergoing a diagnostic lesional biopsy of suspected CCA, the requesting multidisciplinary team should guide the radiologist undertaking the biopsy on which samples to take, including requesting a biopsy of non-lesional liver tissue when the value of additional diagnostic information and management guidance outweighs the additional risk. The targeted lesional core and distant non-lesional core should be submitted to the pathology department as separate specimens so that they can be embedded separately, and any medical liver special stains only undertaken on the non-lesional block, preserving lesional tissue for molecular testing.

23. *An intratumoural block suitable for molecular testing should be taken or identified.*

Molecular testing is required to identify molecular alterations that define patient suitability for personalised therapies. Whilst those treatments currently licenced are only used in the setting of advanced disease, in resection cases we recommend identification of a tumour block at the time of specimen trimming that is suitable for testing should that be clinically indicated.

Block processing and routine tinctorial staining

24. *Five micron or thinner hematoxylin and eosin (H&E)-stained sections should be prepared from each block.*

Section thickness influences the staining characteristics of tinctorial and immunohistochemical staining[47,48] and we recommend that sections no thicker than 5 μm be used. Although a trained pathologist is able to compensate for such variation, computational pathology methods that are in use in a research setting may be unduly influenced by staining variation as a consequence of section thickness.

Microscopic assessment

To aid interpretation of the histological appearance of a tumour by the reporting pathologist, we provide agreed guiding principles rather than explicit rigid recommendations.

25. *Intrahepatic cholangiocarcinoma is usually adenocarcinoma with identifiable glands. However, poorly-differentiated solid cholangiocarcinomas and rarer morphological subtypes (e.g. adenosquamous, squamous, mucinous, signet-ring, clear cell, mucoepidermoid, lymphoepithelioma-like, and sarcomatous) are described.*

Intrahepatic CCA is classically adenocarcinoma with the presence of identifiable glands. However, rarer alternative morphological subtypes are described in the World Health Organisation 'blue book' such that gland formation is not a *sine qua non* for a diagnosis of CCA[49] and this diagnosis should always be considered in a biopsy from a liver lesion with no history of prior or synchronous extrahepatic malignancy.

26. *Desmoplastic stroma is often a prominent feature of intrahepatic cholangiocarcinoma. However, its absence does not preclude a diagnosis of cholangiocarcinoma as its distribution*

within a tumour may mean it is not present in needle biopsy material.

Although prominent desmoplastic stroma is a common feature of iCCA, it is variably distributed within a tumour. The centre of a lesion is often more stroma-rich compared with the periphery that often includes more malignant epithelial elements.[50] This necessarily means that the absence of desmoplastic stroma in a needle core biopsy does not preclude a diagnosis of CCA.

27. *Biliary intraepithelial neoplasia adjacent to an adenocarcinoma with desmoplastic stroma is highly suggestive of cholangiocarcinoma.*

On purely morphological grounds, iCCA (primary hepatic adenocarcinoma) cannot be distinguished from metastatic adenocarcinoma of an extrahepatic primary malignancy. CCA (extrahepatic or large duct type in particular) often develops through a multistep process with preceding dysplastic and *in situ* lesions. Where biliary epithelial neoplasia[51] (BilIN) can be identified in association with invasive adenocarcinoma, more confidence can be ascribed to a diagnosis of CCA. However, in a study of resection cases, BilIN was only identified in 9.5% of cases with iCCA[52] so its absence cannot exclude a diagnosis of CCA.

28. *Large duct-type intrahepatic cholangiocarcinoma is composed of columnar cells.*
29. *Cells of large duct-type intrahepatic cholangiocarcinoma are mucin-producing.*
30. *Cells of large duct-type intrahepatic cholangiocarcinoma are usually arranged in large malignant duct formations or have papillary architecture.*

Large duct-type iCCAs are composed of columnar cells forming large infiltrating glandular structures. In one in-depth morphological study, tumours classified as large duct-type on the cell and gland morphology contained extracellular mucin and intracellular mucin (by H&E staining) in 79% of cases.[53]

31. *Small duct-type intrahepatic cholangiocarcinomas usually show no or minimal mucin production.*
32. *Small duct-type intrahepatic cholangiocarcinomas have a predominant component of cuboidal to polygonal cells.*
33. *Small duct-type intrahepatic cholangiocarcinoma cells are predominantly arranged as small to intermediate-sized tubules and/or anastomosing glands.*

Small duct-type iCCA, in contrast, is composed of cuboidal cells arranged as small-to-intermediate sized tubules or anastomosing glands. Twenty-one percent of cases contained extracellular mucin, and 8% of cases contained intracellular mucin (by H&E staining).[53]

Immunohistochemistry

34. *No immunohistochemical pattern is specific for intrahepatic cholangiocarcinoma.*
35. *Distinction of intrahepatic cholangiocarcinoma from a liver metastasis of a primary upper gastrointestinal, pancreatic, or extrahepatic biliary tumour often cannot be made based on morphology and immunohistochemical profile.*
36. *Intrahepatic cholangiocarcinomas express typical pancreaticobiliary cytokeratins.*

The biliary epithelium has a developmental foregut origin in common with the upper gastrointestinal tract and pancreas. In keeping with this, iCCA typically expresses CK7 and CK19, in common with normal epithelium from those sites and tumours arising from them. This necessarily means that the immunohistochemical profile of these tumours is indistinguishable, there is no specific profile that is diagnostic of CCA,[54] and that no routine immunohistochemical panels should be undertaken. This also serves to preserve tissue for molecular testing.

37. *Immunohistochemistry can help differentiate intrahepatic cholangiocarcinoma from hepatocellular carcinoma.*

Hepatocellular carcinoma (HCC) is typically immunopositive for HepPar1 and Arginase-1 and shows canalicular immunopositivity for CD10 and bile salt exporter protein. However, immunopositivity is often lost in those tumours whose cytological features are less classical, or in poorly differentiated tumours. Further, although HCC is usually immunonegative for CK7 and CK19, these cytokeratins are expressed in a minority of HCCs with likely worse prognosis. This means that the immunohistochemical profile may aid distinguishing iCCA from HCC but cannot provide absolute certainty.

Molecular testing

38. *Molecular testing, where available, including for FGFR abnormalities, IDH mutations, and any other abnormality with therapeutic significance should be undertaken.*

iCCAs have a greater rate of potentially actionable genetic abnormalities than many other tumours, evidenced by recent positive trials for patients whose tumours have *IDH1* mutations or *FGFR2* fusions, and we recommend that molecular testing for any abnormality with potential therapeutic significance be undertaken. Most clinical assays used for molecular testing use FFPE biopsy or resection material. Testing of FFPE material is increasingly reliable and convenient,[18,19] allowing testing of archival cases without the need for rebiopsy to acquire fresh tissue. Molecular testing methods are rapidly evolving and include DNA- and RNA-based next-generation sequencing assays, immunohistochemistry, and fluorescence *in situ* hybridisation. Sequencing panels, assaying several targets simultaneously, are preferred to single gene assays as they provide more usable information and can also estimate tumour mutational burden. The complexity and variety of molecular tests may mean that different tests are undertaken by different laboratories; the ability to test FFPE material also means that samples can easily be sent to an outside laboratory for a test not undertaken locally. A complete discussion of molecular tests and their therapeutic rationale is available in the recent European Society for Medical Oncology (ESMO) Clinical Practice Guideline for biliary tract cancer.[55]

However, we do recognise that such testing is expensive and that targeted treatments are not currently recommended as a first-line option, so the timing and availability of testing will vary.

Given that a biopsy must now provide the information necessary for personalised treatment decisions in addition to a traditional diagnosis, the value of additional sections used for special tinctorial or immunohistochemical stains must be

balanced with the requirement for sufficient material to remain available for molecular testing to guide therapy. Given the absence of any specific immunohistochemical profile that either defines or excludes a diagnosis of iCCA, the usefulness of the tissue for immunohistochemical testing compared with the value of using the same tissue for molecular testing favours the latter. Close collaboration between the reporting histopathologist and the molecular pathology laboratory will allow the most efficient and integrated use of the available tissue.

Written report and interpretation

39. *The American Joint Committee on Cancer/International Union Against Cancer (AJCC/UICCA) staging schema should be used for the reporting of resection specimens, and the stage should be included in the primary report.*

The 8th edition of the AJCC/UICCA TNM staging system for iCCA was published in 2017, modifying the 2010 7th edition. This staging system provides prognostic discrimination and is the most widely used although it may be further modified in time to incorporate additional prognostic factors.[56] We recommend that this staging system be used. However, given recent data indicating that patients with stage II (TNM8) disease and hepatic metastases have poorer outcomes than those without, reporting the number of tumours will allow additional prognostic discrimination.[32]

40. *The results of any molecular analysis and immunohistochemical staining should be included in the primary report.*

Genetic alterations with associated personalised therapeutics may be identifiable in up to half of all iCCA,[57] and the results of the molecular testing undertaken should be included in the primary histopathology report to guide treatment. Currently and depending on geographical location, personalised therapies are available for patients with iCCAs that have *FGFR2* fusions, *IDH1* mutations, *NTRK* fusions, *ERBB2* amplification, high microsatellite instability, and *BRAF* V600E mutations and the results of these tests should be reported. Sequencing panels often identify numerous variants of uncertain significance (VUS).[58] Reporting of such variants is not consistently undertaken by molecular laboratories[59] but there may be value in documentation of VUSs to allow continued data gathering that may lead to some recategorisation. There may also be clinical value in reporting the tumour mutational burden.[60] If insufficient material is available or if testing of any type has failed, this should be documented to allow consideration of additional lesional sampling.

41. *The presence or absence of perineural invasion should be stated in the primary report.*

Perineural infiltration/invasion is a common histological finding in iCCA and should be reported. It may be associated with poorer prognosis.[61,62]

42. *The presence or absence of lymphovascular invasion should be stated in the primary report.*

Although lymphatic invasion is a precursor to lymph node metastasis, histologically-confirmed lymphovascular invasion is an indicator of poorer outcome even in patients without proven lymph node involvement[63] and so should be reported.

43. *The presence or absence of dysplasia should be stated in the primary report.*

The presence of dysplasia at a bile duct resection margin may predict disease recurrence and should be reported.[64]

44. *Small-duct or large-duct designation of intrahepatic cholangiocarcinomas should be stated in the primary report.*

Although classification of iCCA as small duct or large duct-type is made on morphological grounds, the groups show different molecular alterations and, potentially, clinical features and this classification should be reported. The large duct-type has been shown to have a poorer prognosis.[65]

45. *A semiquantitative grading based on the proportion of glands should be stated in the primary report (well-differentiated, <95% of tumour composed of glands; moderately-differentiated, 50–95% of tumour composed of glands; poorly-differentiated, <49% of tumour composed of glands).*

Although there is not a well-established and standardised grading system for iCCA differentiation, the International Collaboration on Cancer Reporting reporting proforma suggests grading based on the proportion of the tumour showing gland formation.[5] Data suggests that patients with less well-differentiated tumours have a poorer prognosis[66–68] and we recommend reporting differentiation based on the proportion of the tumour showing gland formation.

46. *The status of any resection margin(s) should be stated in the primary report.*

Incomplete resection is associated with poorer outcomes in patients undergoing potentially curative resection for iCCA,[33] and so margin status and shortest distance between tumour and margin should be stated in the report.

47. *The status of any lymph nodes should be stated in the primary report.*

Patients with histologically proven lymph node metastasis have a poorer prognosis after resection than patients without lymph node spread.[30,69–71] The number of positive nodes and total number of nodes examined should be conveyed in the histopathology report of a resection specimen.

48. *Confident diagnosis requires the correlation of histomorphology and any immunohistochemical staining profile with available imaging and clinical information. Clinical history, endoscopic investigation, and imaging studies are all pivotal in reaching the correct conclusion.*

A number of consensus agreed statements indicate that a diagnosis of iCCA is not possible on the basis of histological features alone and, therefore, only possible by the integration and understanding of other clinical features provided by an array

of non-pathologist members of the multidisciplinary team required for patient management.

Discussion

The purpose of a pathology report is to provide information to the multidisciplinary team delivering patient care that allows them to offer the optimum treatment. These consensus recommendations provide guidance for all stages of the specimen pathway in the reporting of iCCA to maximise the informational value of the specimen and satisfy this purpose. By explicitly seeking consensus from those delivering patient care (end-users), the development process recognises that the authorised report must be used to have value. We have designed a template to help reporting pathologists, as fillable .pdf and editable .docx files (Supplementary Files 1 and 2).

The pathologist Delphi exercise was designed to reach as wide a geographical range of expert pathologists as possible by asking every national branch of the International Academy of Pathology to nominate a national expert. Pathologists from 28 countries with representation from Asia, Africa, Europe, North America, Oceania, and South America participated. Remarkably, consensus was reached rapidly without any variation related to geography. Further, those participating in the end-user part of the process, were all members of working group 4 ('Epidemiology, clinical characterisation and trials') of the EURO-CHOLANGIO-NET COST action,[72] and also reached immediate consensus about the optimal content of the authorised pathology report both amongst themselves and with the pathologist participants.

Personalised therapies for iCCA defined by the presence of specific molecular features of the tumour are now becoming available. Positive trials for FGFR2 inhibitors[73–75] and IDH1 inhibitors[76] have led to FDA- and EMA-licensed treatments, and tumour agnostic therapies for *BRAF* V600E mutated,[77] MSI-H,[78] *NTRK*-fused,[79] and *RET*-fused[80] tumours are also FDA-licensed. Critically, treatment eligibility requires demonstration of an actionable mutation, and the ESMO guidance recommends the

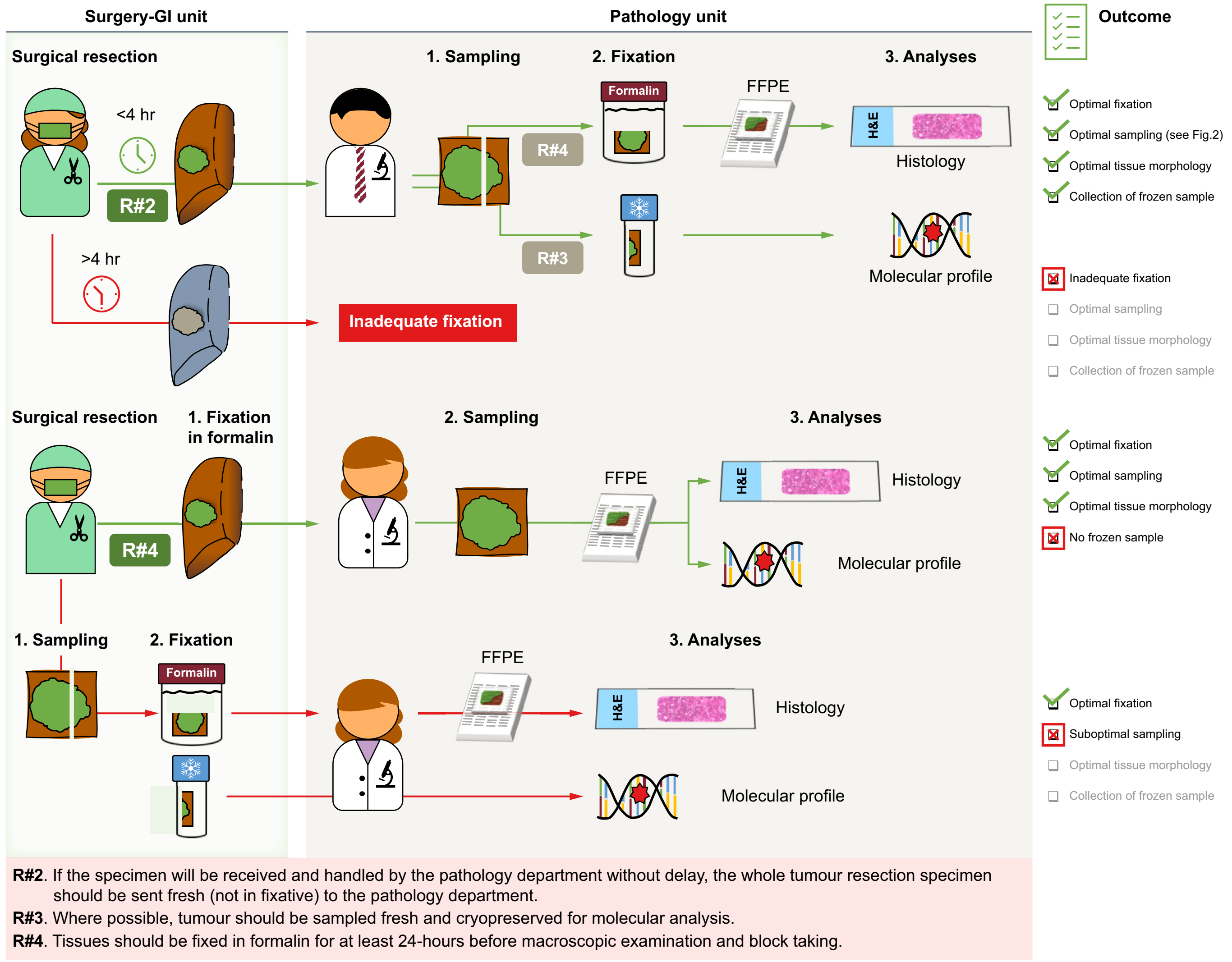

Fig. 1. Schematic of the specimen pathway of a hepatic resection for diagnosis and management of intrahepatic cholangiocarcinoma. (FFPE, formalin-fixed, paraffin-embedded). Critical parts pf the specimen pathway for a resection are represented, with the numbers of the relevant recommendation statements corresponding to those in Table 1.

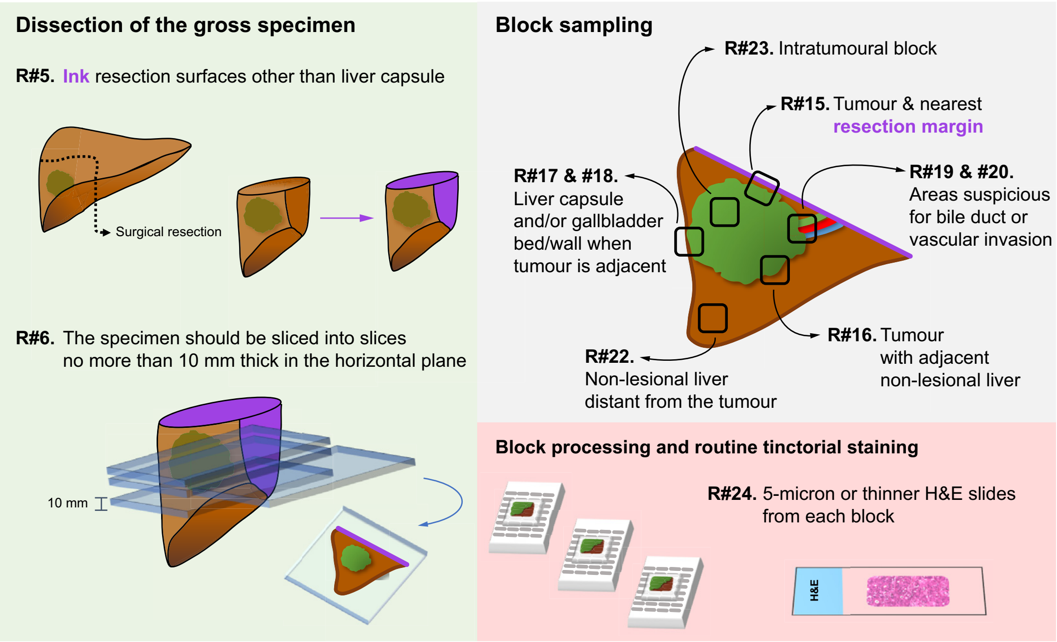

Fig. 2. Schematic of dissection and sampling of a hepatic resection. (H&E, hematoxylin and eosin). Details of the macroscopic assessment, dissection, sampling, and associated slide preparation of the gross hepatic resection specimen, and associated recommendation statements, are indicated as in Table 1.

use of next-generation sequencing panels for CCA.[81] Until such time as circulating tumour molecules can be reliably used to both diagnose the tumour and identify molecular abnormalities, cells and tissues submitted to pathology departments must provide a diagnosis and allow molecular characterisation (Fig. 1), and this may need a change in approach by reporting pathologists. The lack of a specific pattern of immunohistochemical staining for iCCA reduces the value of such staining and, critically, supports the statement (#48) that a confident diagnosis requires correlation with clinical and radiological features. By recognising the importance of clinicopathological correlation over immunohistochemistry, it follows that more tissue should be available in the diagnostic block for molecular testing to guide therapy.

Many of the recommendations relate to activity that allows provision of accurate details about resection specimens (Fig. 2). Although this includes overall stage and features of tumour appearance and spread that have a strong evidence base of their prognostic value, the value of other features is unknown. Specimen handling and reporting of such features will allow their importance to be more systematically determined.

These recommendations have the limitation that only a self-selected group of highly-motivated pathologists and patient-facing clinicians were respondents and these may not be fully representative of those groups as a whole. However, the wide geographical spread of participants was chosen as an attempt to minimise this. We recognise that resources and facilities vary across territories so, wherever possible, recommendations reflect that variation and allow for local modification to suit.

We believe the recommendations in this report have incorporated and evaluated guidance developed and published by disparate national bodies in a transparent manner and represent a unified set of practice points that all pathologists reporting iCCA, wherever they are located, can follow to maximise the value of specimens for patient care through close and systematic communication between all professionals involved in the journey of the patients' tissue sample.

Abbreviations

AGREE-GRS, Appraisal of Guidelines for Research & Evaluation Global Rating Scale; AJCC/UICCA, American Joint Committee on Cancer/International Union Against Cancer; BilIN, Biliary intraepithelial neoplasia; CCA, cholangiocarcinoma; ESMO, European Society for Medical Oncology; FFPE, formalin-fixed paraffin-embedded; dCCA, distal cholangiocarcinoma; HCC, hepatocellular carcinoma; H&E, hematoxylin and eosin; iCCA, intrahepatic cholangiocarcinoma; pCCA, perihilar cholangiocarcinoma; VUS, variants of uncertain significance.

Financial support

No authors received financial support for the conduct of the research and/or preparation of the article.

Conflicts of interest

TK undertakes consultancy work for Perspectum, Clinnovate Health, Kynos Therapeutics, Fibrofind, HistoIndex, Concept Life Sciences, and Resolution Therapeutics, and has received speaker's fees from Incyte Corporation and Servier Laboratories. JB declares research grants (from Incyte, Albireo, Cymabay), personal fees for lectures (from Incyte, AstraZeneca), and consulting roles (for Albireo Pharma, AstraZeneca, Cymabay, Ikan Biotech and OWL-Rubió Metabolomics).

CB received honoraria as speaker (AstraZeneca, Incyte) and consultant (Incyte, Servier, Boehringer Ingelheim, AstraZeneca); received research funds (Avacta, Medannex, Servier) and her spouse is an employee of AstraZneca. CB is supported by a CSO research grant and CRUK Scotland Centre [grant number 100006]. VC received honoraria as speaker (Incyte,

Albireo) and consultant (IPSEN); received research funds (Advanz). GC, DO, MG, BG, and EG have no conflicts of interest to declare.

Please refer to the accompanying ICMJE disclosure forms for further details.

Authors' contributions

Study concept and design: TK, MG, GC. Acquisition of data: TK, DO, MG, GC. Analysis and interpretation of data: all authors. Drafting of the manuscript: TK, DO, MG, GC. Critical revision of the manuscript for important intellectual content: JB, VC, EG, CB, BG. Supervision: TK, MG, GC. Final approval of the submitted version: all authors.

Data availability statement

All data are available from the corresponding author on reasonable request.

Acknowledgements

This article is based upon work from European Network for the Study of Cholangiocarcinoma (ENSCCA) and COST Actions Euro-Cholangio-Net (CA18122) and Precision-BTC-network (CA22125), supported by COST (European Cooperation in Science and Technology).

Supplementary data

Supplementary data to this article can be found online at https://doi.org/10.1016/j.jhepr.2024.101067.

References

Author names in bold designate shared co-first authorship

[1] **Kendre G, Murugesan K**, Brummer T, et al. Charting co-mutation patterns associated with actionable drivers in intrahepatic cholangiocarcinoma. J Hepatol 2023;78:614–626.

[2] Munn Z, Stern C, Aromataris E, et al. What kind of systematic review should I conduct? A proposed typology and guidance for systematic reviewers in the medical and health sciences. BMC Med Res Methodol 2018;18:1–9.

[3] Page MJ, McKenzie JE, Bossuyt PM, et al. The PRISMA 2020 statement: an updated guideline for reporting systematic reviews. BMJ 2021;372:n71.

[4] Bridgewater J, Galle PR, Khan SA, et al. Guidelines for the diagnosis and management of intrahepatic cholangiocarcinoma. J Hepatol 2014;60:1268–1289.

[5] Burt A., Alves V., Coulston A., et al. Intrahepatic cholangiocarcinoma, perihilar cholangiocarcinoma and hepatocellular carcinoma histopathology reporting guide. 2nd ed Sydney, Australia: International Collaboration on Cancer Reporting; 2020. http://www.iccr-cancer.org/datasets/published-datasets/digestive-tractgendeiver. [Accessed 9 November 2023].

[6] Cong W-M, Dong H, Zhu Y-Y, et al. Practice guidelines for the pathological diagnosis of primary liver cancer: 2015 update. World J Gastroenterol 2016;22:9279–9287.

[7] Fernandez Moro C, Fernandez-Woodbridge A, Alistair D'souza M, et al. Immunohistochemical typing of adenocarcinomas of the pancreatobiliary system improves diagnosis and prognostic stratification. PLoS ONE 2016;11:e0166067.

[8] Jiang K, Al-Diffhala S, Centeno BA. Primary liver cancers-part 1: histopathology, differential diagnoses, and risk stratification. Cancer Control 2018;25:1073274817744625.

[9] Khan SA, Davidson BR, Goldin RD, et al. Guidelines for the diagnosis and treatment of cholangiocarcinoma: an update. Gut 2012;61:1657–1669.

[10] Krampitz GW, Aloia TA. Staging of biliary and primary liver tumors: current recommendations and workup. Surg Oncol Clin N Am 2019;28:663–683.

[11] Miyazaki M, Yoshitomi H, Miyakawa S, et al. Clinical practice guidelines for the management of biliary tract cancers 2015: the 2nd English edition. J Hepatobiliary Pancreat Sci 2015;22:249–273.

[12] Washington MK, Berlin J, Branton PA, et al. Protocol for the examination of specimens from patients with carcinoma of the intrahepatic bile ducts. Arch Pathol Lab Med 2010;134:e14–e18.

[13] Wyatt J, Hubscher S, Goldin R. Dataset for histopathology reporting of liver resection specimens (including gall bladder) and liver biopsies for primary and metastatic carcinoma. 2nd ed 2012.

[14] Geller SA, Dhall D, Alsabeh R. Application of immunohistochemistry to liver and gastrointestinal neoplasms: liver, stomach, colon, and pancreas. Arch Pathol Lab Med 2008;132:490–499.

[15] Kawano F, Ito H, Oba A, et al. Role of intraoperative assessment of proximal bile duct margin status and additional resection of perihilar cholangiocarcinoma: can local clearance trump tumor biology? A retrospective cohort study. Ann Surg Oncol 2023;30:3348–3359.

[16] Lenet T, Gilbert RWD, Smoot R, et al. Does intraoperative frozen section and revision of margins lead to improved survival in patients undergoing resection of perihilar cholangiocarcinoma? A systematic review and meta-analysis. Ann Surg Oncol 2022;29:7592–7602.

[17] Rakha E, Ramaiah S, McGregor A. Accuracy of frozen section in the diagnosis of liver mass lesions. J Clin Pathol 2006;59:352–354.

[18] Stenzinger A, Vogel A, Lehmann U, et al. Molecular profiling in cholangiocarcinoma: a practical guide to next-generation sequencing. Cancer Treat Rev 2024;122:102649.

[19] **Fassan M, Angerilli V**, Normanno N, et al. Practical guidelines for molecular testing of cholangiocarcinoma in clinical practice: Italian experts' position paper. Crit Rev Oncol Hematol 2024;194:104224.

[20] Mathieson W, Thomas GA. Why formalin-fixed, paraffin-embedded biospecimens must be used in genomic medicine: an evidence-based review and conclusion. J Histochem Cytochem 2020;68:543–552.

[21] Mole DJ, Fallowfield JA, Sherif AE, et al. Quantitative magnetic resonance imaging predicts individual future liver performance after liver resection for cancer. PLoS ONE 2020;15:e0238568.

[22] Srinivasan M, Sedmak D, Jewell S. Effect of fixatives and tissue processing on the content and integrity of nucleic acids. Am J Pathol 2002;161:1961–1971.

[23] Salerno A, Trent R, Jackson PJ, et al. A rapid and safe method to fix India ink on specimen resection margins. J Clin Pathol 1995;48:689–690.

[24] Guglielmi A, Ruzzenente A, Campagnaro T, et al. Intrahepatic cholangiocarcinoma: prognostic factors after surgical resection. World J Surg 2009;33:1247–1254.

[25] Shimada K, Sano T, Sakamoto Y, et al. Surgical outcomes of the mass-forming plus periductal infiltrating types of intrahepatic cholangiocarcinoma: a comparative study with the typical mass-forming type of intrahepatic cholangiocarcinoma. World J Surg 2007;31:2016–2022.

[26] Ohtsuka M, Ito H, Kimura F, et al. Results of surgical treatment for intrahepatic cholangiocarcinoma and clinicopathological factors influencing survival. Br J Surg 2002;89:1525–1531.

[27] Hyder O, Marques H, Pulitano C, et al. A nomogram to predict long-term survival after resection for intrahepatic cholangiocarcinoma: an Eastern and Western experience. JAMA Surg 2014;149:432–438.

[28] Bagante F, Spolverato G, Merath K, et al. Intrahepatic cholangiocarcinoma tumor burden: a classification and regression tree model to define prognostic groups after resection. Surgery 2019;166:983–990.

[29] Ali SM, Clark CJ, Mounajjed T, et al. Model to predict survival after surgical resection of intrahepatic cholangiocarcinoma: the Mayo Clinic experience. HPB (Oxford) 2015;17:244–250.

[30] Izquierdo-Sanchez L, Lamarca A, La Casta A, et al. Cholangiocarcinoma landscape in Europe: diagnostic, prognostic and therapeutic insights from the ENSCCA Registry. J Hepatol 2022;76:1109–1121.

[31] Sapisochin G, Rodríguez de Lope C, Gastaca M, et al. "Very early" intrahepatic cholangiocarcinoma in cirrhotic patients: should liver transplantation be reconsidered in these patients? Am J Transpl 2014;14:660–667.

[32] Lamarca A, Santos-Laso A, Utpatel K, et al. Liver metastases of intrahepatic cholangiocarcinoma: implications for an updated staging system. Hepatology 2021;73:2311–2325.

[33] Spolverato G, Yakoob MY, Kim Y, et al. The impact of surgical margin status on long-term outcome after resection for intrahepatic cholangiocarcinoma. Ann Surg Oncol 2015;22:4020–4028.

[34] Farges O, Fuks D, Boleslawski E, et al. Influence of surgical margins on outcome in patients with intrahepatic cholangiocarcinoma: a multicenter study by the AFC-IHCC-2009 study group. Ann Surg 2011;254:824.

[35] Ma KW, Cheung TT, She WH, et al. The effect of wide resection margin in patients with intrahepatic cholangiocarcinoma: a single-center experience. Medicine 2016;95:e4133.

[36] Bartsch F, Baumgart J, Hoppe-Lotichius M, et al. Intrahepatic cholangiocarcinoma – influence of resection margin and tumor distance to the liver capsule on survival. BMC Surg 2020;20:61.

[37] Suzuki S, Sakaguchi T, Yokoi Y, et al. Clinicopathological prognostic factors and impact of surgical treatment of mass-forming intrahepatic cholangiocarcinoma. World J Surg 2002;26:687–693.

[38] Mavros MN, Economopoulos KP, Alexiou VG, et al. Treatment and prognosis for patients with intrahepatic cholangiocarcinoma: systematic review and meta-analysis. JAMA Surg 2014;149:565–574.
[39] Kang S-H, Hwang S, Lee Y-J, et al. Prognostic comparison of the 7th and 8th editions of the American Joint Committee on Cancer staging system for intrahepatic cholangiocarcinoma. J Hepatobiliary Pancreat Sci 2018;25:240–248.
[40] Hirohashi K, Uenishi T, Kubo S, et al. Histologic bile duct invasion by a mass-forming intrahepatic cholangiocarcinoma. J Hepatobiliary Pancreat Surg 2002;9:233–236.
[41] Sasaki A, Aramaki M, Kawano K, et al. Intrahepatic peripheral cholangiocarcinoma: mode of spread and choice of surgical treatment. Br J Surg 1998;85:1206–1209.
[42] Palmer WC, Patel T. Are common factors involved in the pathogenesis of primary liver cancers? A meta-analysis of risk factors for intrahepatic cholangiocarcinoma. J Hepatol 2012;57:69–76.
[43] Jeong S, Gao L, Tong Y, et al. Prognostic impact of cirrhosis in patients with intrahepatic cholangiocarcinoma following hepatic resection. Can J Gastroenterol Hepatol 2017;2017:6543423.
[44] Li Y, Li H, Lv P, et al. Prognostic value of cirrhosis for intrahepatic cholangiocarcinoma after surgical treatment. J Gastrointest Surg 2011;15:608–613.
[45] Budau K-L, Sigel CS, Bergmann L, et al. Prognostic impact of tumor budding in intrahepatic cholangiocellular carcinoma. J Cancer 2022;13:2457–2471.
[46] Bartsch F, Baumgart J, Hoppe-Lotichius M, et al. Visceral infiltration of intrahepatic cholangiocarcinoma is most prognostic after curative resection - retrospective cohort study of 102 consecutive liver resections from a single center. Int J Surg 2018;55:193–200.
[47] Chlipala EA, Butters M, Brous M, et al. Impact of preanalytical factors during histology processing on section suitability for digital image analysis. Toxicol Pathol 2021;49:755–772.
[48] Masuda S, Suzuki R, Kitano Y, et al. Tissue thickness interferes with the estimation of the immunohistochemical intensity: introduction of a control system for managing tissue thickness. Appl Immunohistochem Mol Morphol 2021;29:118.
[49] WHO Classification of Tumours Editorial Board. World Health Organization Classification of Tumours of the Digestive System. 5th ed., 1. Lyon: International Agency for Research on Cancer; 2019.
[50] Vijgen S, Terris B, Rubbia-Brandt L. Pathology of intrahepatic cholangiocarcinoma. Hepatobiliary Surg Nutr 2017;6:22–34.
[51] Sato Y, Sasaki M, Harada K, et al. Pathological diagnosis of flat epithelial lesions of the biliary tract with emphasis on biliary intraepithelial neoplasia. J Gastroenterol 2014;49:64–72.
[52] Yoon K-C, Yu Y-D, Kang W-H, et al. Prevalence and clinical significance of biliary intraepithelial neoplasia (BilIN) in cholangiocarcinoma. Am Surg 2019;85:511–517.
[53] Sigel CS, Drill E, Zhou Y, et al. Intrahepatic cholangiocarcinomas have histologically and immunophenotypically distinct small and large duct patterns. Am J Surg Pathol 2018;42:1334–1345.
[54] Shimonishi T, Miyazaki K, Nakanuma Y. Cytokeratin profile relates to histological subtypes and intrahepatic location of intrahepatic cholangiocarcinoma and primary sites of metastatic adenocarcinoma of liver. Histopathology 2000;37:55–63.
[55] Vogel A, Bridgewater J, Edeline J, et al. Biliary tract cancer: ESMO Clinical Practice Guideline for diagnosis, treatment and follow-up. Ann Oncol 2023;34:127–140.
[56] Yamamoto Y, Sugiura T, Okamura Y, et al. The evaluation of the eighth edition of the AJCC/UICC staging system for intrahepatic cholangiocarcinoma: a proposal of a modified new staging system. J Gastrointest Surg 2020;24:786–795.
[57] Tomczak A, Springfeld C, Dill MT, et al. Precision oncology for intrahepatic cholangiocarcinoma in clinical practice. Br J Cancer 2022;127:1701–1708.
[58] Federici G, Soddu S. Variants of uncertain significance in the era of high-throughput genome sequencing: a lesson from breast and ovary cancers. J Exp Clin Cancer Res 2020;39:46.
[59] Vears DF, Sénécal K, Borry P. Reporting practices for variants of uncertain significance from next generation sequencing technologies. s. Eur J Med Genet 2017;60:553–558.
[60] Liddell SS, Chakrabarti S, Wintheiser GA, et al. Tumor mutational burden is a potential predictive biomarker for response to immune checkpoint inhibitors in patients with advanced biliary tract cancer. JCO Precis Oncol 2022:e2200003.
[61] Shirai K, Ebata T, Oda K, et al. Perineural invasion is a prognostic factor in intrahepatic cholangiocarcinoma. World J Surg 2008;32:2395–2402.
[62] Zhang Z, Zhou Y, Hu K, et al. Perineural invasion as a prognostic factor for intrahepatic cholangiocarcinoma after curative resection and a potential indication for postoperative chemotherapy: a retrospective cohort study. BMC Cancer 2020;20:270.
[63] Patel SH, Kooby DA, Staley CA, et al. The prognostic importance of lymphovascular invasion in cholangiocarcinoma above the cystic duct: a new selection criterion for adjuvant therapy? HPB (Oxford) 2011;13:605–611.
[64] Shiraki T, Kuroda H, Takada A, et al. Intraoperative frozen section diagnosis of bile duct margin for extrahepatic cholangiocarcinoma. World J Gastrointest 2018;24:1332–1342.
[65] Akita M, Fujikura K, Ajiki T, et al. Dichotomy in intrahepatic cholangiocarcinomas based on histologic similarities to hilar cholangiocarcinomas. Mod Pathol 2017;30:986–997.
[66] Chang ME, Lei HJ, Chen MH, et al. Evaluation of prognostic factors and implication of lymph node dissection in intrahepatic cholangiocarcinoma: 10-year experience at a tertiary referral center. J Chin Med Assoc 2017;80:140–146.
[67] Lu J, Li B, Li FY, et al. Long-term outcome and prognostic factors of intrahepatic cholangiocarcinoma involving the hepatic hilus versus hilar cholangiocarcinoma after curative-intent resection: should they be recognized as perihilar cholangiocarcinoma or differentiated? Eur J Surg Oncol 2019;45:2173–2179.
[68] Wirasorn K, Ngamprasertchai T, Chindaprasirt J, et al. Prognostic factors in resectable cholangiocarcinoma patients: carcinoembryonic antigen, lymph node, surgical margin and chemotherapy. World J Gastrointest Oncol 2013;5:81–87.
[69] Jolissaint JS, Soares KC, Seier KP, et al. Intrahepatic cholangiocarcinoma with lymph node metastasis: treatment-related outcomes and the role of tumor genomics in patient selection. Clin Cancer Res 2021;27:4101–4108.
[70] Jutric Z, Johnston WC, Hoen HM, et al. Impact of lymph node status in patients with intrahepatic cholangiocarcinoma treated by major hepatectomy: a review of the National Cancer Database. HPB 2016;18:79–87.
[71] Zhang X-F, Xue F, Dong D-H, et al. Number and station of lymph node metastasis after curative-intent resection of intrahepatic cholangiocarcinoma impact prognosis. Ann Surg 2021;274:e1187–e1195.
[72] European cholangiocarcinoma network. 2017; https://eurocholangionet.eu/. [Accessed 9 November 2023].
[73] Abou-Alfa GK, Sahai V, Hollebecque A, et al. Pemigatinib for previously treated, locally advanced or metastatic cholangiocarcinoma: a multicentre, open-label, phase 2 study. Lancet Oncol 2020;21:671–684.
[74] Javle MM, Roychowdhury S, Kelley RK, et al. Final results from a phase II study of infigratinib (BGJ398), an FGFR-selective tyrosine kinase inhibitor, in patients with previously treated advanced cholangiocarcinoma harboring an FGFR2 gene fusion or rearrangement. JCO 2021;39(Suppl. 3): 265.
[75] Goyal L, Meric-Bernstam F, Hollebecque A, et al. Futibatinib for FGFR2-rearranged intrahepatic cholangiocarcinoma. N Engl J Med 2023;388:228–239.
[76] Abou-Alfa GK, Macarulla T, Javle MM, et al. Ivosidenib in IDH1-mutant, chemotherapy-refractory cholangiocarcinoma (ClarIDHy): a multicentre, randomised, double-blind, placebo-controlled, phase 3 study. Lancet Oncol 2020;21:796–807.
[77] Subbiah V, Lassen U, Élez E, et al. Dabrafenib plus trametinib in patients with BRAFV600E-mutated biliary tract cancer (ROAR): a phase 2, open-label, single-arm, multicentre basket trial. Lancet Oncol 2020;21:1234–1243.
[78] Marabelle A, Le DT, Ascierto PA, et al. Efficacy of pembrolizumab in patients with noncolorectal high microsatellite instability/mismatch repair–deficient cancer: results from the phase II KEYNOTE-158 study. J Clin Oncol 2020;38:1–10.
[79] **Drilon A**, **Siena S**, Ou S-HI, et al. Safety and antitumor activity of the multi-targeted pan-TRK, ROS1, and ALK inhibitor entrectinib (RXDX-101): combined results from two phase 1 trials (ALKA-372-001 and STARTRK-1). Cancer Discov 2017;7:400–409.
[80] Subbiah V, Cassier PA, Siena S, et al. Pan-cancer efficacy of pralsetinib in patients with RET fusion–positive solid tumors from the phase 1/2 ARROW trial. Nat Med 2022;28:1640–1645.
[81] Mosele F, Remon J, Mateo J, et al. Recommendations for the use of next-generation sequencing (NGS) for patients with metastatic cancers: a report from the ESMO Precision Medicine Working Group. Ann Oncol 2020;31:1491–1505.

Journal of Hepatology, Volume *6*

Supplemental information

Recommendations on maximising the clinical value of tissue in the management of patients with intrahepatic cholangiocarcinoma

Timothy Kendall, Diletta Overi, Maria Guido, Chiara Braconi, Jesus Banales, Vincenzo Cardinale, Eugenio Gaudio, Bas Groot Koerkamp, and Guido Carpino

Recommendations on maximising the clinical value of tissue in the management of patients with intrahepatic cholangiocarcinoma

Timothy Kendall, Diletta Overi, Maria Guido, Chiara Braconi, Jesus Banales, Vincenzo Cardinale, Eugenio Gaudio, Bas Groot Koerkamp, Guido Carpino

Table of contents

Supplementary Methods....................2

Supplementary Text....................5

Supplementary Figures....................20

Supplementary Tables....................23

Supplementary References....................26

Supplementary Methods

Systematic review methods

The review question was developed based on the recommended Population, phenomena of Interest, Context framework - Population: Individuals involved in the histopathological reporting of iCCA, phenomena of interest: recommendations or guidelines specific to the histopathological reporting of iCCA, context: any part of the entire specimen pathway from receipt in the histopathology department to issue of the final report of an iCCA. The final review question was: What recommendations are there for the histopathological reporting of iCCA?

Protocol and registration

The systematic review protocol was prospectively registered on the Open Science Framework online repository[1].

Eligibility criteria

Resources were eligible for inclusion if they contained: 1) guidance (explicit guidelines or expert recommendations) and/or a checklist, which are (2) histopathological reporting-related, relevant to (3) iCCA, and (4) published in 1996 or later. There were no restrictions by language or publication type.

Information sources and search strategy

The search strategy was devised after examining the literature on MEDLINE and EMBASE as well as an internet search on Google Scholar to identify a comprehensive set of relevant search terms. The full search strategies for all database and web searches are available in the review protocol[1]. Keywords such as '(histolo*; OR patholo*)' AND '(checklist; OR guideline; OR checklist)' AND '(cholangiocarcinoma; OR bile duct cancer)' were used in the search through the databases of MEDLINE (Ovid), EMBASE (Ovid) and Cochrane Library. Web searches on Google and Google Scholar were performed using the advanced search function, with the keywords '(Pathology; OR Histology; OR Biomarkers)' AND '(Guideline; OR Checklist)' AND 'cholangiocarcinoma'. Only the first 3 pages (30 results) from each internet search were screened. The databases and search engines were searched from 1 January 1996 to 31 December 2021. Besides the year of publication, no other limitations were placed on the searches.

Selection of guidance resources

Titles and abstracts of the records were screened by two reviewers; TJK screened all the records and MG and GC acted as second reviewers. In cases where an abstract was not available, the full text of the article was retrieved. Resources which both reviewers selected for inclusion were subjected to full-text review, while any disagreements were arbitrated by a third reviewer (MG or GC). Two reviewers (TK and GC) independently reviewed the full-texts; any disagreements were discussed with a third reviewer (MG) and resolved by consensus.

Quality assessment of guidance resources

Each eligible resource was examined by two reviewers (TK and GC) using the Appraisal of Guidelines for Research & Evaluation Global Rating Scale (AGREE-GRS) quality assessment instrument[2]. Guidance resources were not excluded based on the quality scores. The scores were used to compare the variation in methodological quality across resources, which were categorised into low, moderate and high quality based on the AGREE-GRS scores as previously described[3].

Data extraction and data items

Using best practice from the JBI Manual for Evidence Synthesis[4], a pre-specified digital data extraction form was customised in Excel, adapting the JBI 'Text and opinion data extraction tool'[5] to incorporate data fields specific to systematic reviews of textual opinion-based evidence, as previously used[3]. The following data items were extracted from each resource:

- Context within specimen reporting pathway – areas of specimen reporting pathway the guidance related to.
- Phenomena of Interest – variables associated with histopathological iCCA reporting guidance (guidance development methodology, organisational and geographical representation in guidance development, clinical regulatory authority referenced, and presence of a reporting checklist).
- Verbatim extracts of guidance text.
- Interpretation of guidance statements; classifying guidance statements as 'explicit' or 'implicit' (explicit – 'distinctly expressing all that is meant; leaving nothing merely implied or suggested': implicit – 'suggested or understood but not directly expressed')[6].

Data from each resource were independently extracted by two reviewers (TK and GC) onto the data extraction form, followed by checks for consistency. Any discrepancies were discussed between the two reviewers and any disagreements resolved by a third reviewer (MG).

Data synthesis and analysis

The best fit framework synthesis method[7–9] that incorporates all elements of the JBI meta-aggregation approach[10,11], was used for synthesis and analysis of descriptive qualitative data. The five stages of this method require 'familiarisation' with the literature to select the appropriate *a priori* framework; the distinct stages of the specimen pathway between specimen receipt in a pathology department and final authorisation of the histopathology report were used. The core group (TK, MG, GC, DO) examined the thematically linked extracted guidance from each source and generated a unifying statement or statements that were considered to accurately capture the meaning of the extracted text.

International Delphi exercise

Ethical considerations

The UK Research Integrity Office's Code of Practice for Research and the Universities UK (UUK) Concordat to Support Research Integrity adopted as the University of Edinburgh's Research Integrity framework was followed and the project was assessed before study commencement using the UKRIO checklist for good practice in research in advance as a peer opinion study. Information was given to Delphi participants in the introductory page of the survey, and electronic informed consent, including for disclosure of participant name and affiliation at publication, was obtained following General Data Protection Regulation best practices and before survey completion (Supplementary Text 1).

Candidate item generation

The candidate items for the Delphi process were derived from the synthesised guidance generated by the systematic review. The final list was produced after discussion between the core group (TK, MG, GC, DO) allowing revision of, or addition to, the synthesised guidance to generate a complete offering to be assessed by the Delphi panellists. Statements were grouped into the stages of the reporting pathway between receipt of a specimen in the pathology department until the authorisation of the final pathology report.

Participants

Participants for the survey of histopathologists reporting iCCA were selected to include those practicing in multiple territories. The named secretary of every national division of the International Academy of Pathology were contacted to ask for a national expert. To democratise the process, and recent successes leveraging social networks of pathologists, an invitation to self-nominate was made on Twitter, amplified by liver pathologist experts on the network.

For the Delphi survey of 'end-users' (surgeons, oncologists, gastroenterologists) of histopathology reports, the membership list of working group 4 ('Epidemiology, clinical characterization and trials') of the EURO-CHOLANGIO-NET COST action was used.

Those responsible for developing the candidate items did not provide responses in the Delphi process to minimise potential bias.

Rating

Participants were asked to rate the importance of items on a nine-point Likert-type item scale, where '9' represented 'critical' and '1' represented 'not important'. Participants were able to use free-text boxes to comment about their scores, about the wording of the statement, or offer other items that were not included as candidates.

An item was defined as 'consensus-in' when >70% of participants scored the item as '7-9 (critical)' with <15% scoring '1-3 (not important)'. An item was classified as 'consensus-out' when >70% of participants scored an item '1-3' and <15% of participants scored it '7-9'. All other combinations were considered to be 'no consensus', in accordance with previous research that used similar nine-point scales[3].

'End-user' participants were only asked to rate statements regarding what should be included in the written report.

Procedure

A minimum of two rounds was chosen to allow participants to reflect and change scores between rounds. A third round was not required because complete agreement was reached by the end of the second round.

Communication between the researchers and Delphi participants used private, individual email addresses so participants remained anonymous to one another. The Delphi was conducted via Google Forms with links to the Delphi provided in the emails. In Round One (22nd September 2022), participants were given instructions, a consent form, and the items to rate. They were asked to complete the survey by 16th October 2022 and reminder emails were sent to non-responders.

Round Two (17th November 2022) invitations were sent only to those participants who completed Round One. Participants were advised which items did not reach consensus in Round One and how these had been amended based on feedback, and asked to re-rate these items. They were informed that their scores from Round One would be carried forward for any item for which a new score was not returned and asked to complete the survey by 2nd December 2022. Reminder emails were sent to non-responders.

Analysis

In Rounds One and Two, response rate, median score and item consensus scores were calculated. Data were analysed in R. In addition, free text comments were reviewed to determine if items needed amendment or additional items required inclusion.

Supplementary Text

Supplementary Text 1 Delphi consent statement

'So that the process is transparent, we will include your name and affiliation in a list of participants to be published with the guidelines. By providing us with these details you are giving your consent for us to do this. We are also collecting other data about your experience but this information and participant responses to Delphi items will only be published in aggregated form, not attributed to individuals.

You are able to withdraw your consent at any time prior to submission of the guidelines for publication by contacting Dr Tim Kendall, University of Edinburgh (Tim.Kendall@ed.ac.uk).'

Supplementary Text 2 Verbatim extracted guidance

Bridgewater et al., 2014

4. Microscopic assessment

A pathological diagnosis of iCCA is based on the WHO classification of biliary tract cancer showing an adenocarcinoma or mucinous carcinoma. The most common histological findings of an iCCA are those of an adenocarcinoma showing tubular and/ or papillary structures and a variable fibrous stroma.

Although it has been proposed that the diagnosis of iCCA can be made based upon a combination of clinical presentation, laboratory analysis, and radiologic evaluation, pathological diagnosis is required for definitive diagnosis in most patients, particularly those with cirrhosis and small hepatic mass lesions as radiographic studies are nonspecific.

Pathological diagnosis is recommended for all patients who will be undergoing systemic chemotherapy or radiation therapy, or enrolling in a therapeutic clinical trial. The sensitivity of liver biopsy for pathological diagnosis will depend upon location, size, and operator expertise. Core biopsies are required for definitive diagnosis. Although a positive liver biopsy will establish a diagnosis, a negative biopsy does not exclude it because of the potential for sampling error.

5. Immunohistochemistry

iCCA needs to be distinguished from benign biliary lesions such as peribiliary glands, reactive ductular proliferation, biliary microhamartomas (von Meyenburg complexes) and bile duct adenomas (peribiliary tract hamartomas), particularly in the presence of inflammation which can result in reactive cellular atypia. The histological appearance of iCCA is similar to that of metastatic adenocarcinoma arising from extrahepatic primary tumors and especially those of foregut origin such as lung, pancreas, esophagus, and stomach. The differentiation of iCCA from metastatic adenocarcinoma cannot be readily ascertained on histological examination. Differentiation between iCCA and mixed HCC tumors may require evaluation of specific markers of hepatocellular or progenitor cell features such as Hep-Par-1, GPC3, HSP70, glutamine synthetase, EpCAM, and CK19. CK19 positivity is not specific for iCCA. The expression of cytokeratin 7 and cytokeratin 20 may be helpful to establish a biliary origin.

7. Written report

The 7th edition of the AJCC/UICCA staging schema is currently the preferred staging system for resected iCCA.

Burt et al., 2020

2. Macroscopy/cut up/trimming of the gross specimen

When previous therapy has been administered microscopic examination of the entire tumour should be done when feasible. For selective sampling, sampling an entire cross section has been recommended if the tumour is <2 centimetres (cm) with an additional section for each 1 cm for larger tumours.

Additional sampling of areas that appear grossly viable is often necessary.

The following guidelines are provided for intrahepatic tumours:

- Tumour with nearest hepatic resection margin (when this is close enough to the tumour to be included in the block).
- Other blocks of tumour with adjacent liver tissue (for microscopic vascular invasion (MiVI)).
- Liver capsule if there is a possibility of capsular invasion, i.e., where there is subjacent tumour and overlying adherent tissue or macroscopic capsular invasion. Where the capsule appears intact over subcapsular tumour, with a smooth shiny surface, histology is not required to confirm capsular integrity.
- Gallbladder bed and wall where there is adjacent intrahepatic tumour.
- Any site macroscopically suggestive of macrovascular or bile duct invasion.
- Background liver (taken as far away as possible from the tumour).

A block of representative background liver should be taken at a distance from the tumour, whether or not it looks abnormal macroscopically.

The number of tumours is also recognized as an important prognostic factor in intrahepatic cholangiocarcinoma. Location of all tumours (HCC and intrahepatic cholangiocarcinoma) should be reported since this is important for correlation with imaging when this is available.

Distinction from metastatic adenocarcinoma is based on the presence of a single or dominant intrahepatic mass and absence of a known extra-hepatic primary tumour.

4. Microscopic assessment

According to the current WHO Classification 5th edition, the two main histological subtypes of intrahepatic CC are the large duct type, arising in the intrahepatic large ducts and composed of mucin secreting tumour cells and the small duct type (non-mucin secreting and mainly occurring in the hepatic periphery).

Cholangiolocarcinoma (CLC) and intrahepatic cholangiocarcinoma with ductal plate malformation pattern are subtypes of small duct intrahepatic CC.

Four tumour growth patterns of intrahepatic cholangiocarcinoma are described: the mass-forming type, the periductal infiltrating type, the intraductal growth type and the mixed type. Mass-forming intrahepatic cholangiocarcinoma (65% of cases) forms a well-demarcated nodule growing in a radial pattern and invading the adjacent liver parenchyma. The periductal-infiltrating type of cholangiocarcinoma (6% of cases) spreads in a diffuse longitudinal growth pattern along the bile duct, and the intra-ductal growth type (4% of cases) shows a polypoid or papillary tumour within the dilated bile duct lumen.

Definitive criteria for histological grading of cholangiocarcinomas have not been established; however, the following semiquantitative grading system based on the proportion of gland formation within the tumour is commonly used for intrahepatic cholangiocarcinomas:

- Well differentiated (more than 95% of tumour composed of glands)
- Moderately differentiated (50% to 95% of tumour composed of glands)
- Poorly differentiated (up to 49% of tumour composed of glands)

5. Immunohistochemistry
Immunohistochemical markers that may be used to demonstrate hepatocellular differentiation in poorly differentiated tumours include arginase-1, Hep Par 1, pCEA, CD10, BSEP, AFP and glypican-3.

7. Written report
Record extent of invasion.

Record perineural invasion.

Record lymphovascular invasion.

Record other pathologies.

For dysplasia involving large bile duct radicles we recommend the use of the BilIN and Intraductal papillary neoplasm of the bile ducts (IPNB) classifications described in the WHO 5th edition guidelines, both of which distinguish low grade from high grade change.

Margin status - For intrahepatic cholangiocarcinoma there are a few publications citing margin status as a prognostic factor on multivariate analysis. A systematic review of intrahepatic CC did not include margin status among significant prognostic factors.

Lymph node metastases in intrahepatic and perihilar cholangiocarcinoma have been identified as an important predictor of prognosis.

Cong et al., 2016
1. Specimen fixation
The surgeons should fill an Application Form of Pathological Examination describing the clinical diagnosis, location and type of lesions and number of tissues. The surgical margin, suspected lesions, important vessels and bile duct margin should be marked with a dye or suture by surgeons. Small resected tissues, such as lymph nodes, should be placed into different containers and labeled with corresponding descriptions.

To maximally preserve the integrity of intracellular nucleic acids and proteins for avoiding autocytolysis, tumor specimens should be transferred to the Department of Pathology as soon as possible after resection, ideally within 30 min after surgical removal for sectioning and fixation.

The fresh specimens should be cut consecutively into 1cm thick multiple sections at the maximal diameter; a portion should remain unfixed fresh or cryopreserved for molecular examination.

At room temperature, tissues should be fixed in a neutral formalin solution (v:v, 1:4-5) for 12-24 h and embedded in paraffin.

2. Macroscopy/cut up/trimming of the gross specimen
Hepatic tumor samples should be collected using the 7 point baseline sampling protocol

The location and number of liver tissues collected should be determined as appropriate according to the size, shape and number of the liver tumors as well as the adjacent liver tissues.

Because the detection rate of MVI and satellite nodules is related to the extent of adjacent liver tissues, it is necessary to describe the size of the adjacent liver tissues, and the suspected lesions should be sampled after reviewing several sections.

When the tumor tissue is close to the surgical margin, sampling should be done at the region vertical to the margin closest to the cancer. When the tumor tissue is far away from the surgical margin, sampling should be done parallel to the surgical margin. The status of the surgical margin should be determined using the section with maximal area.

3. Block processing and routine tinctorial staining

Sections of 5µm thickness should be cut from each block and stained with hematoxylin and eosin for histological examination.

4. Microscopic assessment

Adenocarcinoma is the most common histological type of ICC, although it may also present in other special histological and cell types and its differentiation degree can be classified as well, intermediate and poor.

Tumor growth patterns, including peritumoral invasion, capsule invasion, MVI and satellite nodules.

The presence of chronic liver disease, such as chronic hepatitis or hepatic cirrhosis. Although there are many systems for grading and staging chronic viral hepatitis, a simple histologic scoring system is recommended for routine pathological diagnosis, such as the Scheuer scoring system, etc. Furthermore, Masson's trichrome staining and reticular fiber staining can be routinely undertaken to assess the degrees of hepatic fibrosis and lobule reconstruction, respectively.

5. Immunohistochemistry

Currently used biomarkers for liver cancer are somewhat imperfect in their diagnostic specificity and sensitivity; thus, a biomarker panel in combination with other tissue-specific markers could represent a useful tool for diagnosis and differential diagnosis between benign and malignant hepatocellular tumors, HCC and ICC, other specific types of hepatic tumors, and primary and metastatic liver cancer.

Although immunohistochemical staining for CD34 does not directly label hepatic parenchymal cells, it is valuable for determining the extent of MVD and examining its unique distribution pattern in different liver tumors. For instance, a diffuse staining pattern is indicative for HCC, a scattered staining pattern for ICC, a patchy staining pattern for HCA, and a cord-like staining pattern for focal nodular hyperplasia, etc.

Fernandez Moro et al., 2016

5. Immunohistochemistry

A combination of "generic" adenocarcinomamarkers (CK7, CK19, BerEP4, and polyclonal CEA) together with vimentin and a limited number of "pancreatobiliary" markers (MUC1 and CA19-9) defines its characteristic immunohistochemical profile. The present study identified cytoplasmic WT1 as a novel marker for intrahepatic cholangiocarcinoma. The characteristic immunohistochemical profile of intrahepatic cholangiocarcinoma positively supports its pathological diagnosis, which no longer needs to be regarded as a diagnosis of exclusion (of metastatic adenocarcinoma).

Geller et al., 2008

4. Microscopic assessment

Cholangiocarcinoma most often must be differentiated from metastatic carcinoma. Cholangiocarcinoma is almost always a desmoplastic tumor and often is mucin producing.

5. Immunohistochemistry

The immunophenotype of CCA is generally similar to that of extrahepatic pancreaticobiliary carcinomas. Almost all CCAs react with CK7, CK19, CEA (both monoclonal and polyclonal; noncanalicular pattern), and MOC-31. In contrast to other pancreaticobiliary carcinomas, CCAs are more likely to react with CK7 and less likely to react with CK17, CK20, and p53. Cytokeratins can also be useful in differentiating HCC from CCA, as can thyroid transcription factor 1 and claudins. Cholangiocarcinoma can also react with CA 125, and differentiation from mullerian carcinomas can be difficult, although CCA is almost never estrogen receptor positive. Newer studies with the mucins (eg, MUC4, MUC5AC, MUC5B, MUC6) suggest that they may be useful in the subclassification of CCAs and may be useful in evaluating prognosis.

Jiang et al., 2018

2. Macroscopy/cut up/trimming of the gross specimen

Pathologic staging depends on pathologic documentation of the anatomic extent of disease, whether the primary tumor has been completely removed. If a biopsied tumor is not resected for any reason and if the highest T and N categories or the M1 category of the tumor can be confirmed microscopically, the criteria for pathologic classification and staging have been satisfied without complete removal of the primary cancer. The AJCC eight edition classifies the T category according to vascular invasion, extent of invasion, and the number of tumors.

4. Microscopic assessment

Compared to other types of cancer involving liver, cholangiocarcinomas are the ones that elicit a marked desmoplastic fibrotic reaction.

It has been well recognized that cholangiocarcinomas demonstrate a wide spectrum of growth patterns. Tumors can be composed of irregular cystic, branching/glandular tubular structures or as irregular aggregates of infiltrating glands.

Cholangiocarcinomas do not always show easily identifiable tubular contours; instead, tumor cells often grow in a nodular pattern or form sheets of solid nests that mimic neuroendocrine neo-plasms and/or HCC. Other tumors may show copious mucin components, resembling mucinous adenocarcinoma or colloid tumors.

It should be kept in mind that for any cholangiocarcinoma, it is common to see a variety of the abovementioned histomorphological features in the same lesion.

Focal features of sarcomatoid or clear cell morphology are not uncommon.

Cholangiocarcinoma tumor cells frequently extend along the portal tracts by growing within the connective tissue, without directly invading into the bile ducts in the vicinity, even though this pattern of tumor invasion can be seen in not only cholangiocarcinoma but also other types of tumors, including metastatic carcinomas. Also, tumor cells can colonize and extend along the bile ducts and intermingle with native benign bile ducts and adjacent reactive ductules.

It should always be kept in mind that cholangiocarcinomas are neurotropic, and tumor cells frequently approach nerve tracts, with frequent perineural and intraneural involvement. In practice,

this pattern can be very challenging to recognize, especially during frozen section evaluation; more so when the primary tumor is small and when there is no clinical evidence of metastatic disease.

Another malignant behavior of cholangiocarcinomas is lymphovascular invasion. Astute microscopic examination and extensive tumor sectioning is essential in reaching such a diagnosis.

The usual intraluminal “dirty” necrosis, frequently associated with colorectal primary adenocarcinomas, are not typically seen in cholangiocarcinomas.

Histologically, cholangiocarcinoma follows a stepwise carcinogenesis process through a precursor lesion: BilIN, ranging from low to high grade (carcinoma in situ). The presence of these precursor lesions in the vicinity of an intrahepatic adenocarcinoma, combined with marked desmoplastic reaction and high-grade cytological atypia, is highly suggestive of cholangiocarcinoma, instead of metastatic colorectal adenocarcinoma; the latter usually demonstrates palisading, pencil-like nuclei and intraluminal “dirty” necrosis. However, a frequent obstacle encountered in practice is the small size of the biopsy material, which inevitably restricts the differential list and limits reaching a definitive diagnosis due to small areas of useful diagnostic material.

5. Immunohistochemistry

By immunostaining, cholangiocarcinomas are strongly positive for CK-7 and CAM5.2 and show cytoplasmic labeling by p-CEA. Mouse monoclonal antibody that recognizes human EPCAM on cell membrane (MOC31) is positive in around 90% of cases, whereas CK-19 is positive in 70% to 80% of cases. Interestingly, there have been findings to suggest that peripheral cholangiocarcinomas are more likely to be CK-7 positive but CK-20 negative, whereas central counterparts tend to be positive for both CK-7 and CK-20. CD56 also is reported to be positive in peripheral cholangiocarcinomas, which does not necessarily mean there is neuroendocrine differentiation, if other neuroendocrine markers are negative.

One particularly useful immunostain marker for confirming cholangiocarcinoma is S100P, which is negative in benign biliary epithelium but usually positive in cholangiocarcinomas, especially when combined with U3 small nucleolar ribonucleoprotein protein (IMP3) and a protein that in humans is encoded by the von Hippel -Lindau tumor suppressor gene (pVHL). In comparison, HCCs, including all the variants, are predominantly positive in the described pattern for the immunostain markers introduced earlier, including arginase, HepPar-1, glypican 3, CD34, CD10, and p-CEA; HCCs are also positive for AE1/3 and CAM5.2 in almost all cases but remain negative mostly for CK-7 and CK-20. In contrast, cholangiocarcinomas are mostly negative for the markers for HCC, except for p- CEA, which could be positive in various adenocarcinomas, irrespective of the organs of origin.

Rarely, HepPar-1 could be positive in cholangiocarcinoma cases, with foci of labeling, but given the negativity by other HCC markers and adenocarcinoma histomorphology and immunoprofiles, a diagnosis of cholangiocarcinoma should be beyond the question; this occasional cellular labeling is not specific.

In addition to HCC, a metastatic process from another organ or site is also frequently suspected when facing a potential cholangiocarcinoma, such as tumors originating in gastrointestinal luminal sites, especially those from colorectal regions.

It should be made clear to the entire clinical team that there are no specific markers that can be dependable for diagnosing cholangiocarcinoma. Cholangiocarcinomas are usually positive for CK-7, and sometimes for CK- 20, similar to upper gastrointestinal tract and lung adenocarcinomas. A positive Thyroid Transcription Factor 1 (TTF-1) or napsin-A immunostain would strongly favor

pulmonary origin; however, occasional lung tumors are negative for TTF-1 or napsin-A by immunostaining; these cases require detailed histomorphological evaluation and systemic radiological and clinical correlation to narrow down the most likely origin of the tumor. Likewise, the positive CK-20 labeling and/or focal CDX-2 immunoreactivity does not necessarily indicate gastrointestinal tract or colorectal origin.

In practice, immunohistochemical studies (such as cytokeratin-7 (CK-7), cytokeratin-19 (CK-19), cytokeratin-19 (CK-20), CDX-2, and carbohydrate antigen 19-9 (CA-19-9), in an attempt to tell one potential primary source from another one. However, none of these immunophenotypes are specific for telling cholangiocarcinoma; they are just frequently seen from pancreatobiliary and cholangiolar primaries.

Subsequently, it is extremely important to correlate radiological, clinical, and histopathological findings in order to draw a correct conclusion.

7. Written report

The pathological diagnosis of primary intrahepatic cholangiocarcinoma still mostly remains one of exclusion, because of a lack of specific markers. However, a novel RNA platform using in-situ hybridization for albumin RNA has shown specificity for primary liver cancers, including CC.80 However, since this test is not widely available, diagnosis of primary intrahepatic cholangiocarcinoma still requires exclusion of all other other possibilities by analyzing histomorphology, immunoprofiles, imaging studies, as well as clinical evidence. Clinical history, endoscopic investigation, and ima- ging studies are all pivotal in reaching the correct conclusion.

Frequently, making a diagnosis of cholangiocarcinoma especially the intrahepatic ones, instead of a metastatic adenocarcinoma from another organ or site, can be difficult and challenging, more so when there are none or limited clinical and radiological information available for analysis. The clinical, histological, and molecular information are all essential for decision-making and management planning.

Clinically, cholangiocarcinomas are associated with chronic biliary inflammation and regeneration, as frequently seen in PSC, fluke infestation, chronic hepatitis C and hepatitis B viral infection, profession-related chronic exposure to chemicals, and pancreatobiliary malfunction. On the other hand, history of colorectal, gastrointestinal, or gynecological malignancies as well their stages are helpful in determining the possibility of dealing with a metastatic tumor.

It is very important to compare the histomorphology of the primaries and the adenocarcinoma in the liver to see whether there is any similarities; unfortunately in the setting of a comprehensive cancer center, it is not unusual to face a newly identified intrahepatic adenocarcinoma with no or minimal history and no previous cases for morphological comparison.

To summarize briefly, in order to decide whether an intra-hepatic adenocarcinoma is a primary cholangiocarcinoma or a metastatic process, morphological and ancillary test results need to be utilized in combination with the clinical and radiological findings to reach the most likely conclusion regarding the true nature of the tumor.

Khan et al., 2012

2. Macroscopy/cut up/trimming of the gross specimen

Macroscopic features of intrahepatic CC Intrahepatic CCs are whiter and firmer than HCCs as they contain more desmoplastic stroma. They occur more commonly in non-cirrhotic livers than HCCs and are divided into four macroscopic types.

4. Microscopic assessment

Over 90% of CCs are adenocarcinomas and are classified according to the percentage of tumour composed of glandular tissue. Some types of adenocarcinoma are not graded (eg, carcinoma in situ, clear cell adenocarcinoma and papillary adenocarcinoma). Signet ring cell carcinoma is graded as 3 and small cell carcinoma as 4.

Tumours are usually adenocarcinomas and have prominent desmoplastic stroma. However, except in cases where there is co-existing biliary dysplasia, it may not be possible, even with immunohistochemistry, to differentiate between CC and metastatic tumour. Examples of this include intraductal papillary neoplasm with associated invasive neoplasia, and mucinous cystic neoplasm with associated invasive neoplasia.

5. Immunohistochemistry

Distinguishing intrahepatic CC from metastatic adenocarcinoma and other primary liver tumours can be difficult. Accurate differentiation, particularly from foregut metastases (lung, oesophagus, stomach, pancreas), often cannot be made histologically. Other modalities, especially imaging, are essential.

Immunohistochemistry panels including CK7, CK19, CK20, CDX-2, TTF-1, oestrogen/progesterone receptors and PSA, depending on clinical context, can be helpful. CCs are usually CK7 positive and CK20 negative.

In distinguishing HCC from CC, lack of mucin production and expression of HepPar-1, CD10 and glypican-3 by HCC are useful.

7. Written report

CC staging is based on the tumour-node-metastasis (TNM) system. The 7th edition of the TNM classification introduced a specific staging system for intrahepatic CC, separate from HCC, providing better prognostic information.

Surgical resection specimens should be reported systematically, for example, according to Royal College of Pathologists' guide- lines. The final report should include the following information:

Tumour - a. Histological type b. Histological grade c. Extent of invasion (according to the TNM system) d. Blood/lymphatic vessel invasion e. Perineural invasion: this is common and has prognostic significance.

Margins - These must be adequately sampled because local recurrence is related to involvement of the margins. This is particularly important because extrahepatic CC may be multifocal in up to 5% of cases.

Regional lymph nodes - To stage lymph nodes accurately, the node groups must be specifically identified. Peripancreatic nodes located along the body and tail of the pancreas are considered sites of distant metastasis.

Additional pathological findings - These must be noted if present (eg, carcinoma in situ, sclerosing cholangitis).

Metastases - Metastases to other organs or structures should be reported.

Krampitz et al., 2019

4. Microscopic assessment

Typically, pathologic analysis of biopsy specimens reveals adenocarcinoma, and the challenge is differentiating primary intrahepatic cholangiocarcinoma from metastasis from other gastrointestinal or pancreatic primary tumors, often requiring additional immunohistochemical evaluation.

7. Written report

Recognition of the biological and epidemiologic differences between hepatocellular carcinoma and intrahepatic cholangiocarcinoma led to the development of a distinct tumor, node, metastasis (TNM) staging system for intrahepatic cholangiocarcinoma that has been updated in the Eighth Edition of the American Joint Commission on Cancer (AJCC) Cancer Staging Manual.

Miyazaki et al., 2015

2. Macroscopy/cut up/trimming of the gross specimen

Since the ductal margin status has an im- pact on patients' prognosis, the histological assessment of the ductal resection margin should be performed intraoperatively.

4. Microscopic assessment

Biliary intraepithelial neoplasia (BilIN) and intraductal papillary neoplasm of bile duct (IPNB) are premalignant lesions for bile duct cancer.

Washington et al., 2010

2. Macroscopy/cut up/trimming of the gross specimen

Sections should be prepared from each major tumor nodule, with representative sampling of smaller nodules if macroscopically different in appearance

The evaluation of margins for total or partial hepatectomy specimens depends on the method and extent of resection. It is recommended that the surgeon be consulted to determine the critical foci within the margins that require microscopic evaluation. The transection margin of a partial hepatectomy may be large, rendering it impractical for complete examination. In this setting, grossly positive margins should be microscopically confirmed and documented. If the margins are grossly free of tumor, judicious sampling of the cut surface in the region closest to the nearest identified tumor nodule is indicated. In selected cases, adequate random sampling of the cut surface may be sufficient. outcome for intrahepatic cholangiocarcinoma.

Histologic examination of a regional lymphadenectomy specimen usually involves examination of 3 or more lymph nodes.

4. Microscopic assessment

Combined or mixed hepatocellular-cholangiocarcinoma accounts for less than 5% of primary liver carcinomas and should show histologic evidence of both hepatocellular differentiation and bile duct differentiation, such as production of mucin.

A system based on the proportion of gland formation within the tumor is suggested:

- Grade X Grade cannot be assessed
- Grade 1 Well differentiated (more than 95% of tumor composed of glands)
- Grade 2 Moderately differentiated (50%–95% of tumor composed of glands)
- Grade 3 Poorly differentiated (5%–49% of tumor composed of glands)
- Grade 4 Undifferentiated (less than 5% of tumor composed of glands)

Three tumor growth patterns of intrahepatic cholangiocarcinoma are described: the mass-forming type, the periductal-infiltrating type, and mixed mass-forming/periductal-infiltrating type. Mass-forming intrahepatic cholangiocarcinoma (60% of cases) forms a well-demarcated nodule growing in a radial pattern and invading the adjacent liver parenchyma (Figure 2). In contrast, the periductal-infiltrating type of cholangiocarcinoma (20% of cases) spreads in a diffuse longitudinal growth pattern along the bile duct. The remaining 20% of cases of intrahepatic cholangiocarcinoma grow in a mixed mass-forming/periductal-infiltrating pattern.

7. Written report

The histologic examination of the bile ducts at the cut margin is recommended to evaluate the lining epithelium for in situ carcinoma or dysplasia. If the neoplasm is found near the surgical margin, the distance from the margin should be reported. For multiple tumors, the distance from the nearest tumor should be reported.

The TNM staging system of the American Joint Committee on Cancer and the International Union Against Cancer applies to all primary carcinomas of the intrahepatic bile ducts and mixed hepatocellular-cholangiocarcinomas. It does not apply to hepatic sarcomas or to metastatic tumors of the liver.

Wyatt et al., 2012

2. Macroscopy/cut up/trimming of the gross specimen

Specimens can be dissected in the fresh or fixed state. Although formalin penetrates the liver poorly, intrahepatic tumours are usually clearly demarcated within the liver and examination after 24–48 hours does not significantly impair morphology.

Specimen hardening following fixation facilitates accurate slicing.

If fresh tumour is required, this can be obtained either by slicing the specimen fresh after painting the resection margin, or (if identifiable from the external appearance) by excising a portion of tumour through the capsule, so long as the capsule appears intact and is not covered by adherent fatty tissue which may result from underlying capsular breach by the tumour. The surfaces of the specimen other than the capsule (i.e. parenchymal resection plane, extrahepatic biliary tree, any tissue adherent to the liver capsule) should be painted with ink or silver nitrate to allow identification in histological sections.

Block-taking strategy for all liver specimens:

- Tumour with nearest hepatic resection margin (when this is close enough to the tumour to be included in the block).
- Other blocks of tumour with adjacent liver tissue (for microscopic vascular invasion).
- Liver capsule if there is a possibility of capsular invasion, i.e. where there is subjacent tumour and overlying adherent tissue or macroscopic capsular invasion. Where the capsule appears intact over subcapsular tumour, with a smooth shiny surface, histology is not required to confirm capsular integrity.
- Gall bladder bed where there is adjacent intrahepatic tumour.
- Any site macroscopically suggestive of vascular or bile duct invasion. Background liver (taken as far away as possible from the tumour).

The number of tumour blocks will depend on the tumour type, but should include samples from areas of differing macroscopic appearance in heterogeneous tumours.

A block of representative background liver should be taken, whether or not it looks abnormal macroscopically. The appearance should be included in the text description of the specimen, but is a core item in the dataset proforma only in the histology section, since microscopy provides the more reliable assessment of fibrosis/cirrhosis.

The following additional blocks are required as appropriate:

- where there is tumour tissue close to the hepatic hilum, the hilum should be sampled to include large vessels. Specifically label blocks of main left or right portal vein or bile duct, if present hepatic vein margin (if there is tumour nearby) extrahepatic biliary tree (when included)
- gall bladder – optional when this is macroscopically normal

The site of lymph nodes should be specified if known (hilar, hepatic artery, portal vein, cystic duct). More distant nodes (coeliac axis, periduodenal, periaortic) may be submitted separately by the surgeon. Large nodes that do not show macroscopic involvement should be serially sliced and embedded in their entirety since nodes at this site are often enlarged as a result of reactive changes and may harbour micrometastases, especially in cholangiocarcinoma.

Record the segments resected and the specimen weight after opening the gall bladder and rinsing out the bile.

The specimen dimensions (antero-posterior, medio-lateral and supero- inferior) should also be measured, particularly in cases where much of the specimen is occupied by tumour.

When included with the specimen, record the length of extrahepatic duct, number and site of lymph nodes, size and appearance of gall bladder.

The specimen should be sliced at right angles to the parenchymal resection plane, and preferably in the horizontal plane to facilitate correlation with preoperative cross-sectional imaging. Slices should be as thin as possible, and no more than 10 mm thick. The minimum size of tumours detectable by imaging is now less than 5 mm.

Record the number, site, maximum diameter and distance from hepatic margin of the tumour(s). For multiple tumours, the sites should be recorded in the text of the report in such a way that allows correlation with preoperative imaging. For example, this can conveniently be recorded by numbering the horizontal slices from the top, and specifying the slices and approximate segments of each tumour. 'Multiple tumours' encompasses satellitosis, multifocal tumours and intrahepatic metastases. The presence of satellite nodules should be noted.

The appearance of the background liver (normal, bile-stained, fibrotic/cirrhotic) should be recorded. It is good practice to keep a photographic record of the macroscopic features of the specimen for use during MDT meetings.

The specimen should be inspected carefully for macroscopically apparent vascular invasion, and any suspected vascular invasion should be sampled for histological confirmation. Involvement of the main left or right branch of the portal vein or any of the three main hepatic veins should be specifically recorded, as this information is relevant to TNM staging.

The type of intrahepatic tumour may not be known until histology, however the same core items are required for all:

- type of specimen

- specimen weight
- specimen dimensions (where orientation is known, provide antero-posterior, medio-lateral and superior-inferior dimensions)
- tumour number and size
- presence of satellite lesions (regarded as multiple tumours for TNM staging)
- distance from nearest hepatic resection margin(s)
- for HCC and intrahepatic CC: macroscopic involvement of vessels; specify if main left or right portal vein or a main hepatic vein, and record diameter of vessel involved
- integrity of liver capsule (including bare area on postero-superior aspect) and presence of adherent tissues (e.g. diaphragm) or other organs
- presence and number of lymph nodes received.

3. Block processing and routine tinctorial staining

Needle biopsy - Sectioning and staining - Initially one or two shallow levels stained with H&E should be examined. The pathologist can then determine whether tumour is present and what further investigations are required based on the morphology of the tumour in the biopsy and clinical circumstances.

4. Microscopic assessment

Provide comment on:

- Tumour type
- Tumour differentiation
- Minimum distance to resection margin (hepatic, and where appropriate bile duct or vascular) measured microscopically when less than 5 mm. Microscopic involvement (R1) is generally defined as a clearance of <1 mm.
- Invasion through liver capsule (Glisson's capsule)
- Vascular invasion including confirmation of macroscopic vessel invasion. ? Perineural invasion (cholangiocarcinoma). ? Effects of ablative or neoadjuvant therapy on tumour (if applicable).
- Background liver – presence and stage of fibrosis, and other chronic liver.
- Lymph node involvement (where appropriate); number of nodes with metastasis.

In addition to the location of the CC, the Japanese classification according to the growth pattern into mass-forming, periductal infiltrating and intraductal papillary has gained international recognition. In general, mass-forming CC arises peripherally in the liver. The periductal infiltrating pattern is characteristic of perihilar CC, which includes CC arising in right, left and common hepatic ducts.

Intrahepatic CCs form an expansile tumour mass with obvious borders, and usually arise peripherally in the liver. The maximum diameter is readily determined. The size of the tumour is not a staging criterion since prognosis is independent of size however, it is important for correlation with preoperative imaging.

Staging depends on the number of tumours (single or multiple) and vascular invasion. Unlike HCC, vascular invasion (any size of vessel, pT2a) is distinguished from multiple tumours (pT2b, with or without vascular invasion). Tumour perforating the visceral peritoneum or direct infiltration of adjacent organs constitutes pT3 disease. The periductal infiltrating pattern, when associated with a tumour mass, indicates late-stage disease with a poorer prognosis and is classified as pT4 in TNM7. If the tumour is entirely of periductal infiltrating pattern, consideration should be given as to whether it arises in a main right or left duct; if so, it should be staged as perihilar CC.

Intrahepatic CC typically has a microacinar glandular pattern with central sclerosis, and distinction from metastatic adenocarcinoma particularly from stomach or pancreas is based on the single or dominant intrahepatic mass and absence of a known extra-hepatic primary tumour. Currently, available immunohistochemistry is not contributory.

Most intrahepatic CCs are adenocarcinomas. Rare variants listed in the WHO classification include adenosquamous, squamous, mucinous, signet ring, clear cell, mucoepidermoid, lymphoepithelioma-like (EBV associated) and sarcomatous intrahepatic CCs.

Locally advanced intrahepatic mass-forming CCs invade through the liver capsule and directly into adjacent adherent organs. Perforation of the visceral peritoneum constitutes pT3 disease, and any roughened area of capsule over the tumour should be sampled for histology.

For right intrahepatic CC, the regional lymph nodes include the hilar (common bile duct, hepatic artery, portal vein and cystic duct), periduodenal and peripancreatic lymph nodes. For left intrahepatic CC, regional lymph nodes include hilar and gastrohepatic lymph nodes. For either type of intrahepatic CC, spread to the coeliac and/or periaortic and caval lymph nodes represent distant metastases (M1).

7. Written report

Needle biopsy - The report should include the following:

- the clinical information received with the biopsy
- a macroscopic description including biopsy size
- the presence or absence of tissue from the focal lesion, and of liver tissue (hepatocytes, bile ducts) as histological confirmation that the specimen is indeed from the liver
- a morphological description of the lesion
- the results of any additional stains carried out, including immunohistochemistry a comment on the background liver, if sufficient is included
- a definite diagnosis of the focal lesion where possible, or a discussion of the differential diagnosis. This would include a discussion of tumours compatible with or excluded by immunohistochemistry
- an appropriate SNOMED code

Supplementary Text 3 Delphi participants

Pathologist survey

Prof Jun Akiba (Kurume University Hospital, Kurume, Japan), Dr Mohammed Al-Masqari (The Royal Hospital, Muscat, Oman), Dr Halima Aliyu (Ahmadu Bello University and Ahmadu Bello University Teaching Hospital, Zaria, Nigeria), Prof Venancio Alves (University of Sao Paulo School of Medicine, Sao Paulo, Brazil), Dr Marife Bonifacio (St Luke's Medical Center, Quezon City and Global City, Metro Manila, Philippines), Prof Paula Borralho Nunes (Faculdade de Medicina da Universidade de Lisboa, Lisbon, Portugal), Prof Alastair Burt (Newcastle University, Newcastle upon Tyne, United Kingdom), Dr Anthony Chan (The Chinese University of Hong Kong, Hong Kong, China), Dr LM. Chinchilla-Tábora (University Hospital of Salamanca, Salamanca, Spain), Dr Caroline Cooper (Pathology Queensland, Brisbane, Australia), Dr Alyn Cratchley (Leeds Teaching Hospitals NHS Trust, Leeds, UK), Dr James M Crawford (Donald and Barbara Zucker School of Medicine at Hofstra/Northwell, NY, USA), Dr Sofía del Carmen (Hospital Universitario Marqués de Valdecilla, Santander, Spain), Dr Rouchelle dela Cruz (Makati Medical Center, Makati City, Philippines), Mrs Alba Diaz (Hospital Clínic, Barcelona, Spain), Prof David Driman (London Health Sciences Centre and Schulich School of Medicine and Dentistry, Western University, London, Canada), Dr Fraser Duthie (NHS Greater Glasgow and Clyde, Glasgow,

United Kingdom), Dr Isioma U. Egbuniwe (University of Nottingham/ Nottingham University Hospitals NHS Trust, Nottingham, United Kingdom), Prof Dr Matthias Evert (Institute for Pathology, University of Regensburg, Regensburg, Germany), Dr Kenneth Fleming (Emeritus Fellow, Green Templeton College, University of Oxford, UK), Dr Luiz Freitas (School of Medicine of The Federal University of Bahia, Salvador-Bahia, Brazil), Dr Alessandro Gambella (Department of Medical Sciences - University of Turin, Turin, Italy), Dr Maddi Garmendia (Osakidetza, Donostia - SAN Sebastian, Spain), Prof Eugenio Gaudio (Sapienza University, Rome, Italy), Dr Ryan Gill (University of California, San Francisco, USA), Prof Robert Goldin (Imperial College, London, UK), Dr M. Augusta Gomes Cipriano (Centro Hospitalar Universitário de Coimbra, Coimbra, Portugal), Dr Zachary Goodman (Inova Fairfax Hospital, Falls Church, Virginia, USA), Dr Judit Halász (Semmelweis University Department of Pathology, Forensic and Insurance Medicine, Budapest, Hungary), Prof Seung-Mo Hong (Asan Medical Center, University of Ulsan College of Medicine, Seoul, Republic of Korea), Prof Prodromos Hytiroglou (Aristotle University, Thessaloniki, Greece), Prof Yung-Ming Jeng (National Taiwan University Hospital, Taipei, Taiwan), Dr Haeryoung Kim (Seoul National University Hospital, Seoul, Republic of Korea), Prof Dr Mina Komuta (International University of Health and Welfare, School of Medicine, Narita Hospital, Narita, JAPAN), Associate Prof. Supinda Koonmee (Faculty of Medicine, Khon Kaen university, Khon Kaen, Thailand), Dr Maalini Krishnasamy (Hospital Selayang, Selayang, Malaysia), Dr Hanna Lapsar (Medical laboratory CSD, Kyiv, Ukraine), Dr Wei-Qiang Leow (Department of Anatomical Pathology, Singapore General Hospital, Singapore, Singapore), Dr Regina Lo (The University of Hong Kong, Hong Kong, Hong Kong, China), Dr Paulo Giovanni Mendoza (Cardinal Santos Mendical Center, San Juan Metro Manila, Philippines), Dr Rui Oliveira (Germano de Sousa - Centro de Diagnóstico Histopatológico CEDAP, Coimbra, Portugal), Prof Young Nyun Park (Yonsei university, Seoul, Korea), Dr Federica Pedica (IRCCS San Raffaele Scientific Institute, Milan, Italia), Dr Glenda Lyn Pua (St. Luke's Medical Center, Quezon City, Philippines), Dr Pavitratha Puspanathan (Hospital Pulau Pinang, Pulau Pinang, Malaysia), Prof Alberto Quaglia (Department of Cellular Pathology, Royal Free London and UCL Cancer Institute, London, United Kingdom), Dr Alphania Rahniayu (Dept. Anatomical Pathology, Faculty of Medicine, Universitas Airlangga, Surabaya, Indonesia), Prof Dr Tania Roskams (University of Leuven, Leuven, Belgium), Dr Puja Sakhuja (GB Pant Institute of Postgraduate Medical Education and Research, New Delhi, India), Prof Dr Christine Sempoux (Service of Clinical Pathology, Institute of Pathology, Lausanne University Hospital, University of Lausanne, Lausanne, Switzerland), Dr Aatur Singhi (University of Pittsburgh Medical Center, Pittsburgh, USA), Dr Marini Stephanie (Cipto Mangunkusumo Hospital, Jakarta, Indonesia), Dr Ashley Stueck (Dalhousie University, Halifax, NS, Canada), Prof Dina Tiniakos (Aretaieion Hospital, Medical School, National and Kapodistrian University of Athens, Athens, Greece), Dr Mihhail Vassiljev (North Estonian Medical Center Foundation, Tallinn, Estonia), Prof Dr Joanne Verheij (Amsterdam UMC, Cancer Center Amsterdam, location University of Amsterdam, Department of Pathology, Amsterdam, The Netherlands), Dr Mukul Vij (Dr Rela institute and medical center, Chennai, India), Associate Professor Lai Mun Wang (Changi General Hospital, Singapore, Singapore), Dr Judy Wyatt (Leeds Teaching Hospitals NHS Trust, Leeds, UK)

‘End-user’ survey

Dr Jorge Adeva (Hospital Universitario 12 de octubre, Madrid, Spain), Drr Mohamed Bouattour (Beaujon University Hospital, Clichy, France), Dr Chiara Braconi (University of Glasgow, Glasgow, United KIngdom), Dr Vincenzo Cardinale (Sapienza University of Rome, Rome, Italy), Prof Massimo Colombo (IRCCS San Raffaele Hospital, Milan, Italy), Dr Cristina Dopazo (Hospital Universitario Vall d’Hebron, Barcelona, Spain), Prof Julien Edeline (Centre Eugène Marquis, Rennes, France), Dr Alejandro Forner (University Barcelona, Barcelona, Spain), Dr Bas Groot Koerkamp (Erasmus MC, Rotterdam, Netherlands), Prof Juozas Kupcinskas (Lithuanian University of Health Sciences, Kaunas,

Lithuania), Dr Angela Lamarca (Fundacion Jimenez Diaz University Hospital, Madrid, Spain), Dr Vera Megdanova (University hospital Queen Yoana -ISUL, Sofia, Bulgaria), Dr Anu Planken (North-Estonian Medical Centre, Tallinn, Estonia), Dr Mariano Ponz Sarvise (Clínica Universidad de Navarra, Pamplona, Spain), Dr Francesca Ratti (Hepatobiliary Surgery Division San Raffaele Hospital, Milano, Italy), Professor Bruno Sangro (Clinica Universidad de Navarra, Pamplona, Spain), Prof Juan Valle (University of Manchester / The Christie, Manchester, United Kingdom), Dr Gianpaolo Vidili (University of Sassari, Sassari, Italy), Prof Dr Henning Wege (Medical Center Esslingen, Esslingen, Germany)

Supplementary Figures

Fig. S1

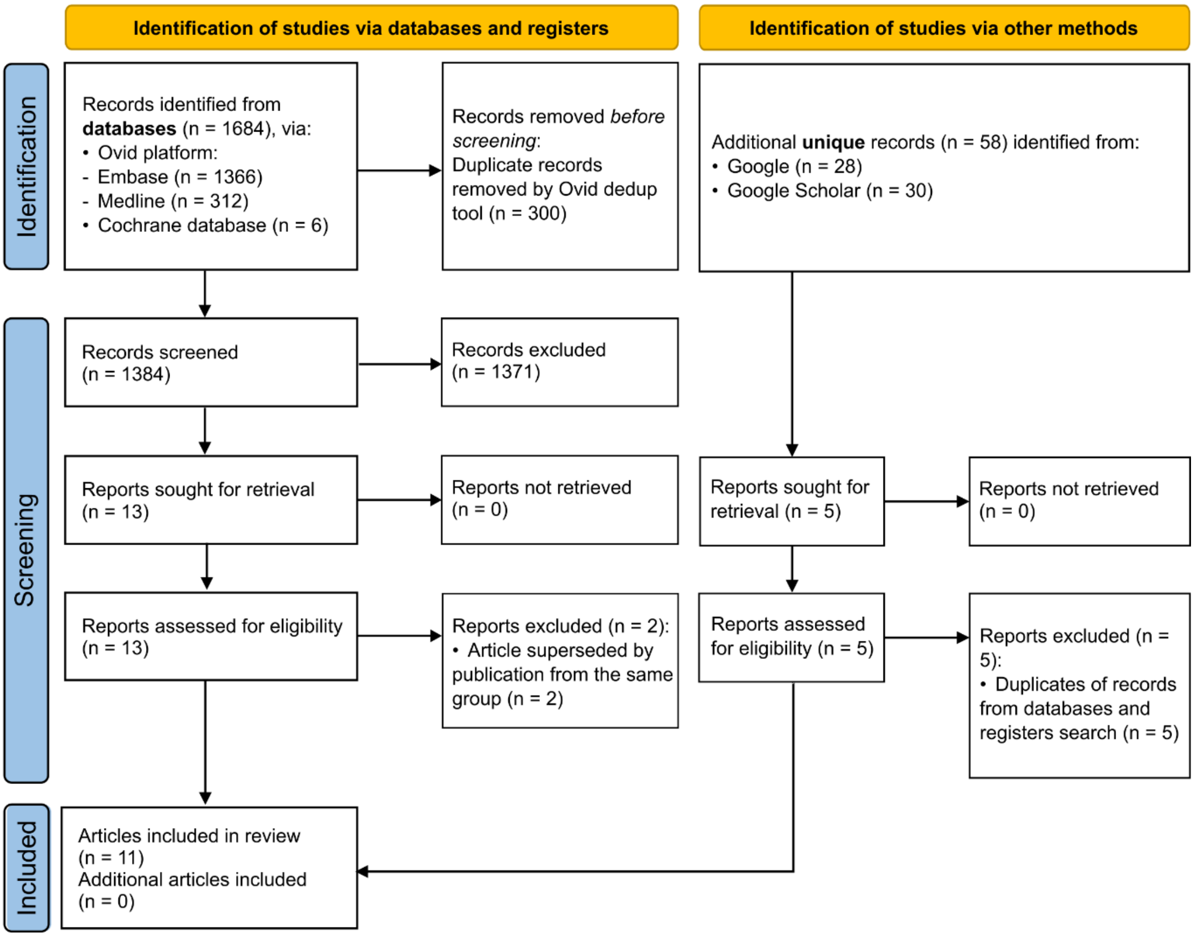

Fig. S1 PRISMA flow diagram of the selection process of guidance resources

Fig. S2

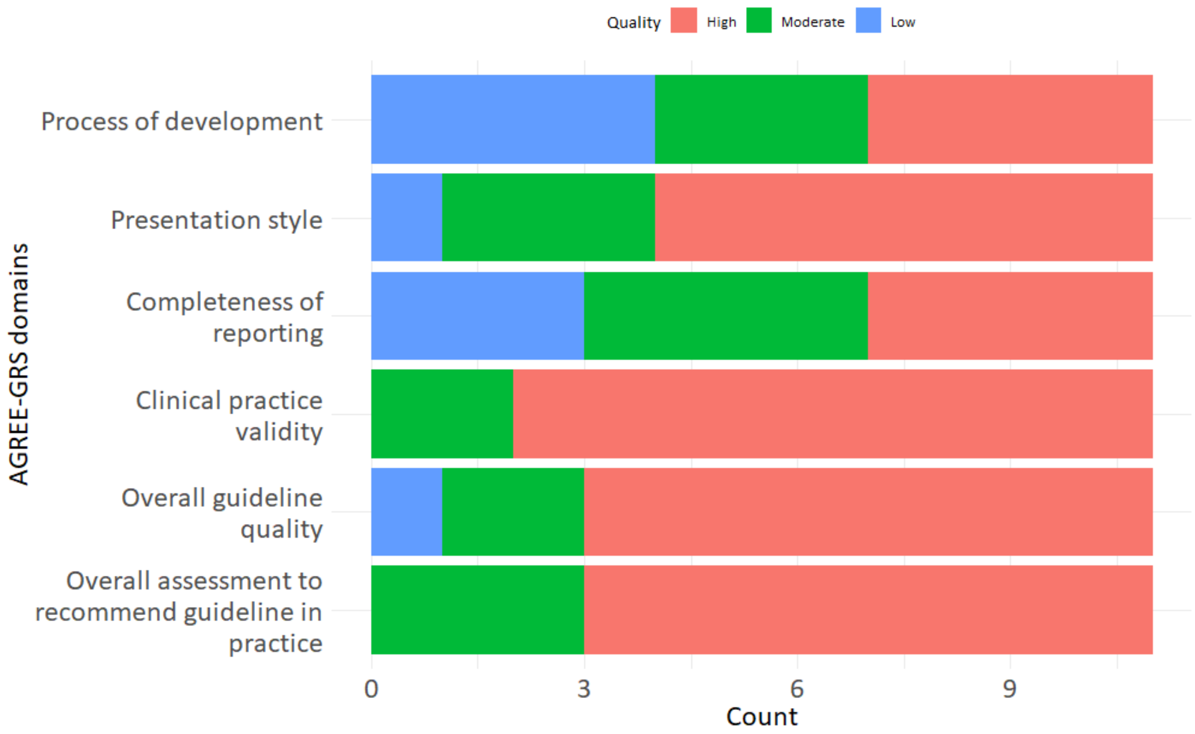

Fig. S2 Number of guidance resources showing low, moderate, and high quality across AGREE-GRS domains

Fig. S3

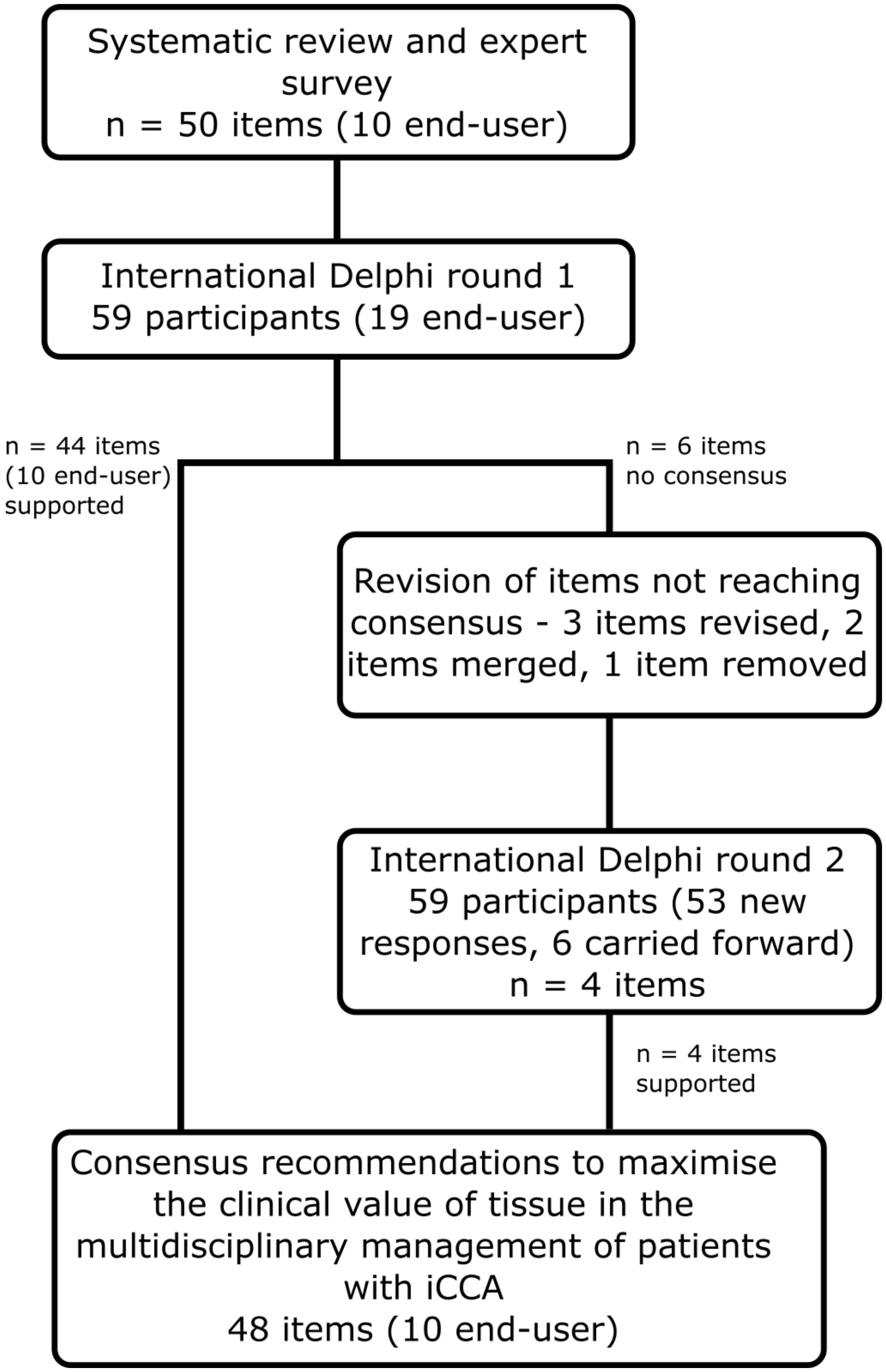

Fig. S3 iCCA recommendation development diagram

Supplementary Tables

Table S1 Systematic review sources and guidance mapping

Category	Number	Percentage	References
1. Specimen fixation	1	9.09	Cong[12]
2. Macroscopy/cut up/trimming of the gross specimen	7	63.64	Burt[13], Cong[12], Jiang[14], Khan[15], Miyazaki[16], Washington[17], Wyatt[18]
3. Block processing and routine tinctorial staining	2	18.18	Cong[12], Wyatt[18]
4. Microscopic assessment	10	90.91	Bridgewater[19], Burt[13], Cong[12], Geller[20], Jiang[14], Khan[15], Krampitz[21], Miyazaki[16], Washington[17], Wyatt[18]
5. Immunohistochemistry	7	63.64	Bridgewater[19], Burt[13], Cong[12], Fernandez Morro[22], Geller[20], Jiang[14], Khan[15]
6. Written report	7	63.64	Bridgewater[19], Burt[13], Jiang[14], Khan[15], Krampitz[21], Washington[17], Wyatt[18]

Table S2 Delphi participant aggregated demographics and experience

Participants	Pathologist, number (total = 59)	User, number (total = 19)
Years experience reporting/managing iCCA		
1 to 5 years	10	1
6 to 10 years	14	7
More than 10 years	35	11
Number of iCCA cases/year		
10 or fewer	12	5
11 to 24	22	6
25 to 49	16	5
50 to 100	7	1
Greater than 100	2	2
Location		
Africa	1	0
Asia	22	0
Europe	27	19
North America	6	0
Oceania	1	0
South America	2	0

Table S3 Delphi responses

Round 1				Round 2			
**Statement**	**% scored 1-3**	**% scored 7-9**	**Item status**	**Statement**	**% scored 1-3**	**% scored 7-9**	**Item status**
Specimen handling and fixation							
a. The bile duct margin in a resection should be assessed by intraoperative frozen section	12.07	53.45	none	a. Although more relevant in resections of perihilar rather than intrahepatic cholangiocarcinomas, assessment of the bile duct margin by intraoperative frozen section may be advantageous if the surgeon considers it to be close to the tumour.	0	91.07	in
b. Whole tumour resection specimens should be sent fresh (not in fixative) to the pathology department	15.79	61.4	none	b. If tumour sampling is routinely undertaken for research, biobanking, or molecular testing and the specimen will be received and handled by the pathology department without delay, the whole tumour resection specimen should be sent fresh (not in fixative) to the pathology department.	1.69	93.22	in
c. Where possible, tumour should be sampled fresh and cryopreserved for molecular analysis	7.02	77.19	in				
d. Tissues should be fixed in formalin for at least 24-hours before macroscopic examination and block taking (for resections) or routine processing (for biopsies)	13.56	77.97	in				
Macroscopy/cut up/trimming of the gross specimen							
a. Dissection - Resection surfaces other than the liver capsule should be painted/inked.	1.72	96.55	in				
b. Dissection - The specimen should be sliced into slices no more than 10 mm thick in the horizontal plane.	3.45	86.21	in				
c. Macroscopic description - The macroscopic growth pattern (mass-forming, periductal infiltrating, intraductal, mixed) should be described.	0	86.44	in				
d. Macroscopic description - The size of tumours should be recorded.	0	100	in				
e. Macroscopic description - The location of tumours should be recorded.	0	94.92	in				
f. Macroscopic description - The macroscopic status of any resection margin(s) and nearest distance to tumour should be recorded.	0	100	in				
g. Macroscopic description - The presence or absence of macroscopic vascular invasion should be recorded.	1.72	93.1	in				
h. Macroscopic description - The presence or absence of macroscopic invasion of named bile ducts should be recorded.	1.72	91.38	in				
i. Macroscopic description - The integrity of the liver capsule should be recorded.	0	96.61	in				
j. Macroscopic description - The macroscopic appearance of the non-lesional liver should be recorded.	0	98.31	in				
k. Block sampling - If the tumour is close enough, a single intact tissue block containing tumour and the nearest resection margin should be taken.	3.39	93.22	in				
l. Block sampling - Tumour with adjacent non-lesional liver should be sampled.	0	100	in				
m. Block sampling - Peripheral liver with the closest overlying liver capsule should be sampled if the tumour is subjacent, overlying tissue is adherent, or there is macroscopic invasion of the liver capsule.	0	96.61	in				
n. Block sampling - The gallbladder bed and wall should be sampled where the tumour is adjacent.	0	98.31	in				
o. Block sampling - Areas suspicious for macrovascular invasion should be sampled.	0	100	in				
p. Block sampling - Areas suspicious for bile duct invasion should be sampled.	0	96.61	in				
q. Block sampling - Any bile duct resection margin(s) should be sampled.	0	98.31	in				
r. Block sampling - Non-lesional liver distant from the tumour should be sampled.	0	96.61	in				
s. Block sampling - An intratumoural block suitable for molecular testing should be taken or identified.	1.69	88.14	in				
Block processing and routine tinctorial staining							
a. 5-micron or thinner H&E-stained sections should be prepared from each block.	0	96.55	in				
Microscopic assessment							
a. For a diagnosis of intrahepatic cholangiocarcinoma to be made, the tumour should be an adenocarcinoma.	11.86	61.02	none	a/b. Intrahepatic cholangiocarcinoma is usually adenocarcinoma with identifiable glands. However, poorly-differentiated solid cholangiocarcinomas and rarer morphological subtypes (e.g. adenosquamous, squamous, mucinous, signet-ring, clear cell, mucoepidermoid, lymphoepithelioma-like, and sarcomatous) are described.	3.77	90.57	in
b. Tumours with a variety of histological patterns can be diagnosed as intrahepatic cholangiocarcinoma i.e. no specific microscopic pattern is required.	17.24	65.52	none	Merged with above			
c. For a histological diagnosis of intrahepatic cholangiocarcinoma to be made, desmoplastic stroma must be present.	32.2	33.9	none	c. Desmoplastic stroma is often a prominent feature of intrahepatic cholangiocarcinoma. However, its absence does not preclude a diagnosis of cholangiocarcinoma as its distribution within a tumour may mean it is not present in needle biopsy material.	3.39	84.75	in
d. Biliary intraepithelial neoplasia (BilIN) adjacent to an adenocarcinoma with desmoplastic stroma is highly suggestive of cholangiocarcinoma.	3.39	83.05	in				
e. Large duct-type intrahepatic cholangiocarcinoma is composed of columnar cells.	3.39	83.05	in				
f. The cells of large duct-type intrahepatic cholangiocarcinoma are mucin-producing.	1.69	81.36	in				
g. The cells of large duct-type intrahepatic cholangiocarcinoma are usually arranged in large malignant duct formations or have papillary architecture.	3.39	84.75	in				
h. Small duct-type intrahepatic cholangiocarcinomas usually show no or minimal mucin production.	0	88.14	in				
i. Small duct-type intrahepatic cholangiocarcinomas have a predominant component of cuboidal to polygonal cells....75	0	96.55	in				
j. Small duct-type intrahepatic cholangiocarcinoma cells are predominantly arranged as small to intermediate-sized tubules and/or anastomosing glands.	1.69	96.61	in				
Immunohistochemistry							
a. No immunohistochemical pattern is specific for intrahepatic cholangiocarcinoma.	5.17	79.31	in				
b. Distinction of intrahepatic cholangiocarcinoma from a liver metastasis of a primary upper gastrointestinal, pancreatic, or extrahepatic biliary tumour often cannot be made based on morphology and immunohistochemical profile.	3.39	89.83	in				
c. Intrahepatic cholangiocarcinomas express typical pancreaticobiliary cytokeratins.	0	93.1	in				
d. Immunohistochemistry can always differentiate intrahepatic cholangiocarcinoma from hepatocellular carcinoma.	29.31	37.93	none	Removed			
e. Immunohistochemistry can help differentiate intrahepatic cholangiocarcinoma from hepatocellular carcinoma.	1.69	94.92	in				
Molecular testing							
a. Molecular testing, where available, including for FGFR abnormalities, IDH mutations, and any other abnormality with therapeutic significance should be undertaken.	0	80.7	in				
Written report and interpretation							
a. The AJCC/UICCA staging schema should be used for the reporting of resection specimens, and the stage included in the primary report.	0	96.61	in				
b. The results of any molecular analysis and immunohistochemical staining should be included in the primary report.	6.9	84.48	in				
c. The presence or absence of perineural invasion should be stated in the primary report.	0	94.92	in				
d. The presence or absence of lymphovascular invasion should be stated in the primary report.	0	98.31	in				
e. The presence or absence of dysplasia should be stated in the primary report.	1.69	91.53	in				
f. Small-duct or large-duct designation of intrahepatic cholangiocarcinomas should be stated in the primary report.	1.72	89.66	in				
g. A semiquantitative grading based on the proportion of glands should be stated in the primary report (well differentiated - more than 95% of tumour composed of glands; moderately-differentiated - 50% to 95% of tumour composed of glands; poorly-differentiated - up to 49% of tumour composed of glands).	6.78	77.97	in				
h. The status of any resection margin(s) should be stated in the primary report.	0	100	in				
i. The status of any lymph nodes should be stated in the primary report.	0	100	in				
j. Confident diagnosis requires the correlation of histomorphology and any immunohistochemical staining profile with available imaging and clinical information. Clinical history, endoscopic investigation, and imaging studies are all pivotal in reaching the correct conclusion.	0	94.92	in				

Supplementary References

1 Kendall T, Carpino G, Guido M. EURO-CHOLANGIO-NET diagnostic histopathology systematic review. Published Online First: 22 October 2021.

2 Brouwers MC, Kho ME, Browman GP, *et al.* The Global Rating Scale complements the AGREE II in advancing the quality of practice guidelines. *J Clin Epidemiol*. 2012;65:526–34.

3 Lim SJ, Gurusamy K, O'Connor D, *et al.* Recommendations for cellular and molecular pathology input into clinical trials: a systematic review and meta-aggregation. *The Journal of Pathology: Clinical Research*. 2021;7:191–202.

4 McArthur A, Klugarova J, Yan H, *et al.* Chapter 4: Systematic Reviews of text and opinion. In: Aromataris E, Munn Z, eds. *JBI Manual for Evidence Synthesis*. JBI 2020. https://doi.org/10.46658/JBIMES-20-05

5 McArthur A, Klugarova J, Yan H, *et al.* 4.3.4.5.2 Textual data extraction - Systematic Reviews of text and opinion. JBI Manual for Evidence Synthesis. 2020. https://wiki.joannabriggs.org/display/MANUAL/4.3.4.5.2+Textual+data+extraction (accessed 18 July 2020)

6 Oxford University Press. Oxford English Dictionary. https://www.oed.com/ (accessed 7 July 2020)

7 Carroll C, Booth A, Cooper K. A worked example of "best fit" framework synthesis: A systematic review of views concerning the taking of some potential chemopreventive agents. *BMC Medical Research Methodology*. 2011;11. doi: 10.1186/1471-2288-11-29

8 Carroll C, Booth A, Leaviss J, *et al.* "Best fit" framework synthesis: Refining the method. *BMC Medical Research Methodology*. 2013;13. doi: 10.1186/1471-2288-13-37

9 Brunton G, Oliver S, Thomas J. Innovations in framework synthesis as a systematic review method. *Research Synthesis Methods*. 2020;11:316–30.

10 McArthur A, Klugarova J, Yan H, *et al.* 4.3.4.5.3 Textual data synthesis - Systematic Reviews of text and opinion. JBI Manual for Evidence Synthesis. 2020. https://wiki.joannabriggs.org/display/MANUAL/4.3.4.5.3+Textual+data+synthesis (accessed 20 July 2020)

11 McArthur A, Klugarova J, Yan H, *et al.* 4.3.4.6.3 Findings of the review - Systematic Reviews of text and opinion. JBI Manual for Evidence Synthesis. 2020. https://wiki.joannabriggs.org/display/MANUAL/4.3.4.6.3+Findings+of+the+review (accessed 20 July 2020)

12 Cong W-M, Dong H, Zhu Y-Y, *et al.* Practice guidelines for the pathological diagnosis of primary liver cancer: 2015 update. *World J Gastroenterol*. 2016;22:9279–87.

13 Burt A, Alves V, Coulston A, *et al. Intrahepatic Cholangiocarcinoma, Perihilar Cholangiocarcinoma and Hepatocellular Carcinoma Histopathology Reporting Guide.* 2nd ed. Sydney, Australia: International Collaboration on Cancer Reporting 2020. http://www.iccr-cancer.org/datasets/published-datasets/digestive-tract/liver

14 Jiang K, Al-Diffhala S, Centeno BA. Primary Liver Cancers-Part 1: Histopathology, Differential Diagnoses, and Risk Stratification. *Cancer Control*. 2018;25:1073274817744625.

15 Khan SA, Davidson BR, Goldin RD, *et al.* Guidelines for the diagnosis and treatment of cholangiocarcinoma: an update. *Gut*. 2012;61:1657–69.

16 Miyazaki M, Yoshitomi H, Miyakawa S, *et al.* Clinical practice guidelines for the management of biliary tract cancers 2015: the 2nd English edition. *J Hepatobiliary Pancreat Sci*. 2015;22:249–73.

17 Washington MK, Berlin J, Branton PA, *et al.* Protocol for the Examination of Specimens From Patients With Carcinoma of the Intrahepatic Bile Ducts. *Archives of Pathology & Laboratory Medicine*. 2010;134:e14–8.

18 Wyatt J, Hubscher S, Goldin R. Dataset for histopathology reporting of liver resection specimens (including gall bladder) and liver biopsies for primary and metastatic carcinoma (2nd edition). 2012.

19 Bridgewater J, Galle PR, Khan SA, *et al.* Guidelines for the diagnosis and management of intrahepatic cholangiocarcinoma. *Journal of Hepatology*. 2014;60:1268–89.

20 Geller SA, Dhall D, Alsabeh R. Application of Immunohistochemistry to Liver and Gastrointestinal Neoplasms: Liver, Stomach, Colon, and Pancreas. *Archives of Pathology & Laboratory Medicine*. 2008;132:490–9.

21 Krampitz GW, Aloia TA. Staging of Biliary and Primary Liver Tumors: Current Recommendations and Workup. *Surg Oncol Clin N Am*. 2019;28:663–83.

22 Fernandez Moro C, Fernandez-Woodbridge A, Alistair D'souza M, *et al.* Immunohistochemical Typing of Adenocarcinomas of the Pancreatobiliary System Improves Diagnosis and Prognostic Stratification. *PLoS ONE*. 2016;11:e0166067.
